# A gut microbial signature for combination immune checkpoint blockade across cancer types

**DOI:** 10.1038/s41591-024-02823-z

**Published:** 2024-03-01

**Authors:** Ashray Gunjur, Yan Shao, Timothy Rozday, Oliver Klein, Andre Mu, Bastiaan W. Haak, Ben Markman, Damien Kee, Matteo S. Carlino, Craig Underhill, Sophia Frentzas, Michael Michael, Bo Gao, Jodie Palmer, Jonathan Cebon, Andreas Behren, David J. Adams, Trevor D. Lawley

**Affiliations:** 1 Host–Microbiota Interactions Laboratory, Wellcome Sanger Institute, Hinxton, UK; 2 Experimental Cancer Genetics, Wellcome Sanger Institute, Hinxton, UK; 3 Olivia Newton-John Cancer Research Institute, La Trobe University School of Cancer Medicine, Melbourne, Victoria Australia; 4 Department of Medical Oncology, Austin Health, Melbourne, Victoria Australia; 5 Central Clinical School, Monash University, Melbourne, Victoria Australia; 6 European Molecular Biology Laboratory, European Bioinformatics Institute, Hinxton, UK; 7 Center for Experimental and Molecular Medicine, Amsterdam UMC, Amsterdam, Netherlands; 8 Department of Medical Oncology, Monash Health, Melbourne, Victoria Australia; 9 Department of Medical Oncology, Alfred Health, Melbourne, Victoria Australia; 10 School of Clinical Sciences, Monash University, Melbourne, Victoria Australia; 11 Department of Medical Oncology, Peter MacCallum Cancer Centre, Melbourne, Victoria Australia; 12 Rare Cancer Laboratory, Walter and Eliza Hall Institute of Medical Research, Melbourne, Victoria Australia; 13 Department of Medical Oncology, Blacktown and Westmead Hospitals, Sydney, New South Wales Australia; 14 Melanoma Institute of Australia, University of Sydney, Sydney, New South Wales Australia; 15 Border Medical Oncology and Haematology Research Unit, Albury–Wodonga Regional Cancer Centre, Albury–Wodonga, New South Wales Australia; 16 Rural Medical School, University of New South Wales, Albury, New South Wales Australia; 17 Sir Peter MacCallum Department of Oncology, University of Melbourne, Melbourne, Victoria Australia

**Keywords:** Translational research, Microbiome, Cancer immunotherapy, Metagenomics

## Abstract

Immune checkpoint blockade (ICB) targeting programmed cell death protein 1 (PD-1) and cytotoxic T lymphocyte protein 4 (CTLA-4) can induce remarkable, yet unpredictable, responses across a variety of cancers. Studies suggest that there is a relationship between a cancer patient’s gut microbiota composition and clinical response to ICB; however, defining microbiome-based biomarkers that generalize across cohorts has been challenging. This may relate to previous efforts quantifying microbiota to species (or higher taxonomic rank) abundances, whereas microbial functions are often strain specific. Here, we performed deep shotgun metagenomic sequencing of baseline fecal samples from a unique, richly annotated phase 2 trial cohort of patients with diverse rare cancers treated with combination ICB (*n* = 106 discovery cohort). We demonstrate that strain-resolved microbial abundances improve machine learning predictions of ICB response and 12-month progression-free survival relative to models built using species-rank quantifications or comprehensive pretreatment clinical factors. Through a meta-analysis of gut metagenomes from a further six comparable studies (*n* = 364 validation cohort), we found cross-cancer (and cross-country) validity of strain–response signatures, but only when the training and test cohorts used concordant ICB regimens (anti-PD-1 monotherapy or combination anti-PD-1 plus anti-CTLA-4). This suggests that future development of gut microbiome diagnostics or therapeutics should be tailored according to ICB treatment regimen rather than according to cancer type.

## Abstract (web summary)

Efforts to identify microbial signatures of response to immune checkpoint blockade suggest that strain-level associations may be cancer type agnostic but cancer therapy specific.

## Main

The past decade has seen an ‘immuno-oncology revolution’ largely driven by the rapid uptake of immune checkpoint blockade (ICB) agents targeting cytotoxic T lymphocyte protein 4 (CTLA-4), programmed cell death protein 1 (PD-1) or programmed death ligand 1 (PD-L1, the ligand of PD-1). Combination ICB (CICB) targeting both PD-1 and CTLA-4 has demonstrated synergistic antitumor activity preclinically^1^ and is now an approved standard of care for patients with diverse cancers, including melanoma^2^, clear-cell renal cell carcinoma^3^, non-small cell lung cancer (NSCLC)^4^, mesothelioma^5^ and hepatocellular carcinoma^6^. However, this success is tempered by the unpredictable nature of responses (seen in only 20–60% of patients across these cancer indications^7^) and the more frequent severe immune-related adverse effects experienced with CICB when compared to anti-PD-1 or anti-PD-L1 monotherapy^8^. Thus, despite the promise it offers, the judicious use of CICB is paramount. Additionally, predictive biomarkers for tumor response and/or toxicity would be highly valuable to guide patient management.

Currently approved tumor-agnostic biomarkers for PD-1 blockade include tumor mutational burden and mismatch repair deficiency^9^; however, both have limitations and rely on available, contemporaneous tumor tissue. A promising ‘tumor-extrinsic’ avenue for predicting ICB response and/or toxicity a priori is assessing a patient’s baseline gut microbiome composition, referring to the community of microbiota (predominantly bacteria) resident within the gastrointestinal tract. Culture-free methods to taxonomically profile fecal microbiomes have progressed from low-resolution 16S rRNA gene sequencing to high-resolution shotgun metagenomics, with studies of clinical cohorts finding associations between baseline *Akkermansia muciniphila* (lung cancer)^10–13^ and *Faecalibacterium prausnitzii* (melanoma)^14–16^ fecal abundances and tumor responses among anti-PD-1 recipients. Unfortunately, previous meta-analyses across metagenomic studies have found limited reproducibility of these candidate microbial biomarkers for ICB response^17–20^. Although this poor reproducibility may be partly attributable to methodological or geographic differences between studies, we hypothesize that species-level taxonomic biomarkers may lack the precision necessary to capture the specific microbial traits associated with ICB response or nonresponse. For example, there is growing awareness of the diversity of intraspecies (strain) variation among commensal bacteria (such as *A. muciniphila* and *F. prausnitzii*), with diverging functional potentials and differing associations with host phenotypes^21,22^.

Here, we performed deep shotgun metagenomic sequencing of baseline fecal samples from patients on the CA209-538 clinical trial of ipilimumab (anti-CTLA-4) and nivolumab (anti-PD-1) for 106 patients with diverse rare cancers (our discovery cohort). Using a bespoke, genome-resolved metagenomics approach, we discovered baseline subspecies (strain-level) gut microbial abundance signatures of response that reproduce between cancer subtypes and externally to published CICB cohorts despite marked cohort heterogeneity. Notably, we found that the predictiveness of signatures trained on CICB cohorts does not extend to anti-PD-1 monotherapy cohorts. This suggests that, although tumor agnostic, different microbiota–host relationships are relevant to distinct ICB regimens.

## Results

### Clinical characteristics of the CA209-538 cohort

**Fig. 1:**
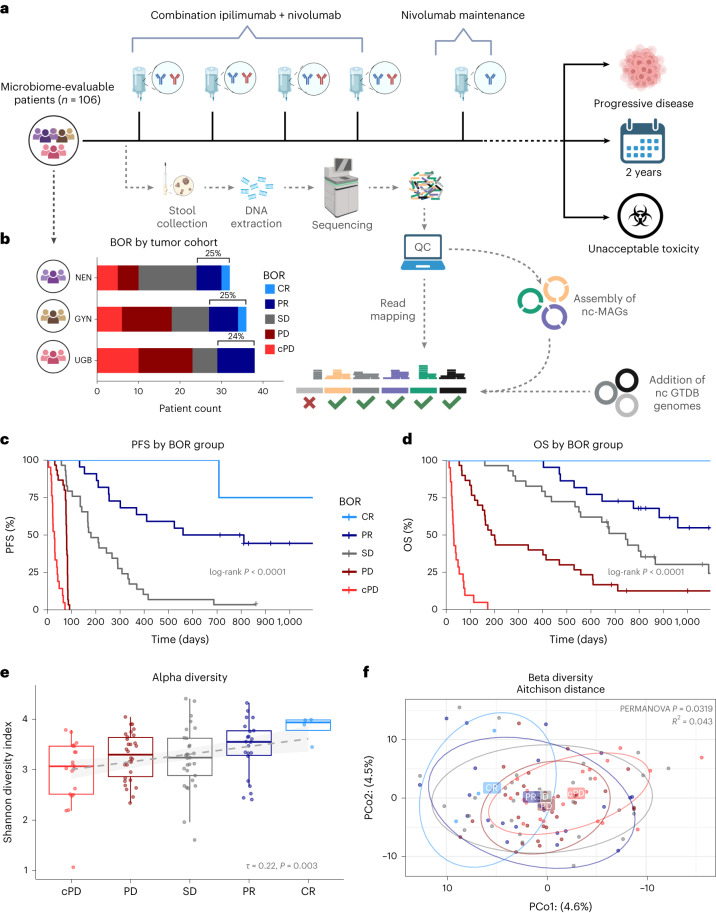
Clinical and gut microbiome compositional differences between responders and nonresponders. **a**, CA209-538 study and microbiome analysis schema (created using BioRender.com). Pretreatment fecal samples were collected from *n* = 106 trial participants and subjected to DNA extraction, shotgun metagenomic sequencing, and analysis using a genome-resolved metagenomics pipeline, involving quality control (QC), de novo assembly of near-complete MAGs (nc-MAGs) and precise read mapping. Further to the standard filters, reads mapping to genomes with <50% coverage breadth were removed. **b**, Bar plot of patient RECIST 1.1 BOR by histology cohort for microbiome-evaluable patients. The percentages of patients with an objective response (PR or CR) are indicated. **c**, Kaplan–Meier curve of PFS stratified by BOR category (cPD *n* = 21, PD *n* = 30, SD *n* = 29, PR *n* = 22, CR *n* = 4). Log-rank test *P* = 2.1 × 10^−42^. **d**, Kaplan–Meier curve of OS stratified by BOR category (cPD *n* = 21, PD *n* = 30, SD *n* = 29, PR *n* = 22, CR *n* = 4). Log-rank test *P* = 1.2 × 10^−34^. **e**, Boxplots of microbiome alpha diversity, as measured by the Shannon diversity index, across BOR categories (cPD *n* = 21, PD *n* = 30, SD *n* = 29, PR *n* = 22, CR *n* = 4). Boxplot center line indicates the median; box limits indicate the upper and lower quartiles; and whiskers indicate 1.5× the interquartile range. The linear model (line of best fit) for the Shannon diversity index and BOR (with shaded 95% confidence interval) is superimposed (in gray). Kendall *τ* and *P* values for the association between the Shannon diversity index and BOR are indicated. **f**, Principal coordinate 1 (PCo1) versus 2 (PCo2) using the Aitchison distance of strain abundances, colored by patient BOR category. Ellipses depict 0.8 of each group’s multivariate *t* distribution. PERMANOVA *P* value and *R*^2^ using 9,999 permutations are indicated.

The CA209-538 clinical trial, titled *‘*A phase 2 trial of ipilimumab and nivolumab for the treatment of rare cancers*’*, is a prospective, multicenter clinical trial (NCT02923934) that enrolled 120 patients with histologically confirmed advanced rare solid-organ cancers across five Australian hospital networks (Methods). Notably, patients had diverse tumor histologies grouped into three prespecified cohorts: upper gastrointestinal and biliary cancers (UGB), neuroendocrine neoplasms (NEN) and rare gynecological tumors (GYN). Most patients (*n* = 108) had received prior systemic anticancer therapies (median of one line (range 0–6 lines)). All participants were treated on trial with combination nivolumab and ipilimumab for up to four doses (induction), followed by nivolumab maintenance for up to 2 years or until progressive disease (PD) or unacceptable toxicity (Fig. 1a). The prespecified secondary endpoint of the trial was to develop ‘tumor-agnostic’ biomarkers for CICB response by leveraging the unique clinical trial design of CA209-538, which included patients with diverse cancers, but with highly standardized clinical and experimental procedures. Therefore, a pretreatment fecal sample was collected from most (*n* = 106) participants (Table 1). No major clinical differences were observed between microbiome-evaluable patients and those who were not sampled (Supplementary Table 1).

**Table 1:** Baseline clinical characteristics by patient BOR category

Characteristics	Best response					*P*
	cPD (*n* = 21)	PD (*n* = 30)	SD (*n* = 29)	PR (*n* = 22)	CR (*n* = 4)	
Histology cohort						
GYN	6 (28.6%)	12 (40.0%)	9 (31.0%)	7 (31.8%)	2 (50.0%)	0.18
NEN	5 (23.8%)	5 (16.7%)	14 (48.3%)	6 (27.3%)	2 (50.0%)	
UGB	10 (47.6%)	13 (43.3%)	6 (20.7%)	9 (40.9%)	0 (0%)	
No. of prior systemic therapies						
Mean (s.d.)	1.43 (0.811)	1.60 (1.22)	1.97 (1.61)	2.00 (1.23)	1.00 (0.82)	0.32
Median (min, max)	1 (0, 3)	1 (0, 5)	2 (0, 5)	2 (1, 5)	1 (0, 2)	
Measurable tumor (mm)						
Mean (s.d.)	115 (82.3)	72.6 (48.4)	84.1 (53.8)	90.0 (78.2)	70.0 (19.1)	0.49
Median (min, max)	108 (17.0, 344)	58.0 (24.0, 219)	77.0 (17.0, 220)	64.0 (11.0, 325)	77.5 (42.0, 83.0)	
Age (years)						
Mean (s.d.)	59.1 (13.9)	56.5 (15.1)	59.9 (14.3)	56.3 (12.5)	65.3 (9.29)	0.85
Median (min, max)	65.0 (20.0, 75.0)	62.5 (26.0, 75.0)	60.0 (22.0, 82.0)	53.5 (38.0, 74.0)	64.0 (57.0, 76.0)	
Sex						
Female	9 (42.9%)	22 (73.3%)	17 (58.6%)	17 (77.3%)	4 (100%)	0.048
Male	12 (57.1%)	8 (26.7%)	12 (41.4%)	5 (22.7%)	0 (0%)	
Site						
AUS	2 (9.5%)	8 (26.7%)	5 (17.2%)	7 (31.8%)	0 (0%)	0.34
BLA	2 (9.5%)	7 (23.3%)	4 (13.8%)	3 (13.6%)	0 (0%)	
BMO	4 (19.0%)	1 (3.3%)	3 (10.3%)	1 (4.5%)	0 (0%)	
MON	8 (38.1%)	6 (20.0%)	11 (37.9%)	3 (13.6%)	2 (50.0%)	
PMC	5 (23.8%)	8 (26.7%)	6 (20.7%)	8 (36.4%)	2 (50.0%)	
Season						
Autumn	9 (42.9%)	13 (43.3%)	12 (41.4%)	11 (50.0%)	1 (25.0%)	0.8
Spring	3 (14.3%)	2 (6.7%)	7 (24.1%)	2 (9.1%)	1 (25.0%)	
Summer	5 (23.8%)	10 (33.3%)	5 (17.2%)	5 (22.7%)	2 (50.0%)	
Winter	4 (19.0%)	5 (16.7%)	5 (17.2%)	4 (18.2%)	0 (0%)	
BMI (kg m^−2^)						
Mean (s.d.)	26.1 (5.74)	27.2 (5.30)	28.6 (6.23)	25.7 (5.05)	25.6 (3.49)	0.97
Median (min, max)	25.1 (19.1, 38.2)	28.2 (18.6, 37.0)	28.2 (18.9, 48.2)	25.0 (18.8, 35.3)	24.6 (22.8, 30.5)	
PPIs (<8 weeks)						
Yes	14 (66.7%)	9 (30.0%)	8 (27.6%)	7 (31.8%)	3 (75.0%)	0.017
No	7 (33.3%)	21 (70.0%)	21 (72.4%)	15 (68.2%)	1 (25.0%)	
Antibiotics (<8 weeks)						
Yes	3 (14.3%)	1 (3.3%)	3 (10.3%)	2 (9.1%)	0 (0%)	0.65
No	18 (85.7%)	29 (96.7%)	26 (89.7%)	20 (90.9%)	4 (100%)	
Platelets (×10^9^ l^−1^)						
Mean (s.d.)	297 (134)	279 (81.1)	224 (97.8)	287 (118)	283 (50.1)	0.32
Median (min, max)	302 (87.0, 603)	273 (133, 575)	189 (62.0, 431)	276 (144, 559)	300 (211, 321)	
Albumin (g l^−1^)						
Mean (s.d.)	30.9 (5.66)	35.0 (5.34)	36.2 (4.34)	35.7 (3.47)	37.0 (2.16)	0.0056
Median (min, max)	32.0 (20.0, 38.0)	36.5 (20.0, 44.0)	37.0 (24.0, 44.0)	36.0 (29.0, 41.0)	36.5 (35.0, 40.0)	
NLR						
Mean (s.d.)	10.7 (15.8)	3.33 (1.99)	3.24 (2.18)	3.66 (2.57)	2.74 (0.144)	0.0033
Median (min, max)	5.27 (2.22, 66.0)	2.92 (0.970, 10.7)	2.72 (1.00, 10.0)	3.27 (0.960, 9.80)	2.70 (2.62, 2.95)	
LDH (U l^−1^)						
Mean (s.d.)	380 (208)	277 (155)	264 (143)	480 (696)	283 (59.2)	0.89
Median (min, max)	296 (149, 945)	215 (162, 898)	219 (128, 912)	302 (140, 3,440)	295 (202, 339)	

Clinical characteristics (metadata) are reported stratified by BOR category for microbiome-evaluable participants (*n* = 106). Numerical metadata are summarized with both means and median values, and statistical associations with BOR (an ordinal variable increasing from cPD to CR) were computed using the Kendall *τ* test. Categorical metadata were analyzed using frequency tables, with statistical associations with BOR computed using the chi-squared test.

AUS, Austin Hospital (Melbourne); BLA, Blacktown Hospital (Sydney); BMO, Border Medical Oncology (Albury); MON, Monash Hospital (Melbourne); PMC, Peter MacCallum Cancer Centre (Melbourne); BMI, body mass index; PPIs, proton-pump inhibitors; LDH, lactate dehydrogenase.

**Extended Data Fig. 1:**
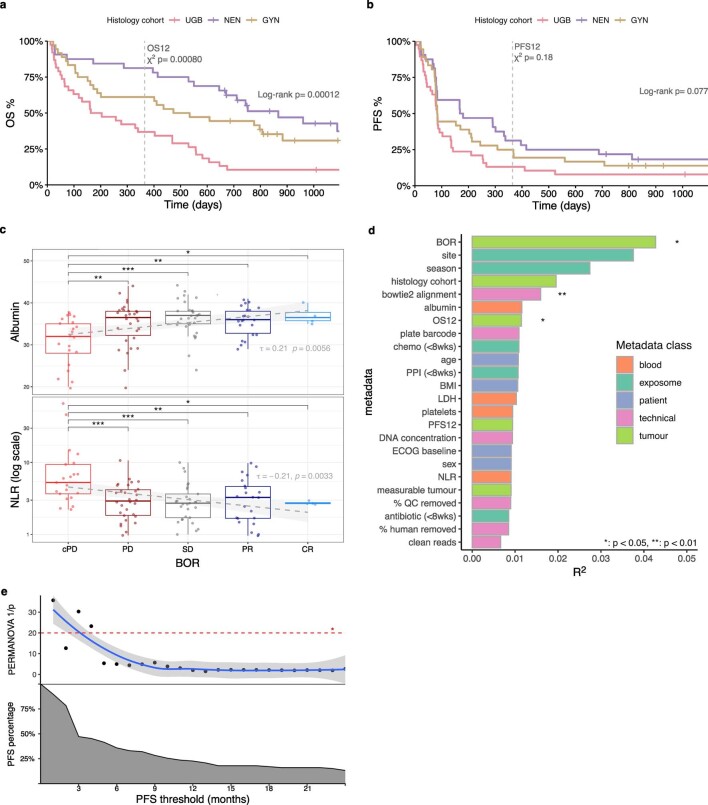
Clinical and gut microbiome characteristics of the CA209-538 clinical trial cohort. **a**, Kaplan-Meier curve of progression-free survival stratified by histology (UGB n = 38, NEN n = 32, GYN n = 36). Log-rank test p-value for OS duration across groups printed. Chi-squared test p-value shown for proportion of OS12 per group printed. **b**, Kaplan-Meier curve of progression-free survival stratified by histology (UGB n = 38, NEN n = 32, GYN n = 36). Log-rank test p-value for PFS duration across groups printed. Chi-squared test of independence p-value shown for proportion of PFS12 per group printed. **c**, Boxplots of patient baseline blood albumin (g/L) and NLR levels (log-transformed) by BOR category (cPD n = 21, PD n = 30, SD n = 29, PR n = 22, CR n = 4). Boxplot centre line= median; box limits= upper and lower quartiles; whiskers= 1.5x interquartile range. Linear model line-of-best-fit for respective variables (albumin and NLR) versus BOR (with shaded 95% confidence interval) superimposed (in grey). Kendall *τ* and p-value for association between respective variables (albumin and NLR) and BOR printed. Pairwise Mann-Whitney U test p-values for cPD vs other groups summarized (*: p < 0.05, **: p < 0.01, ***: p < 0.001). Exact p-values as follows: Albumin: cPD vs PD p = 0.0076, cPD vs SD p = 0.00079, cPD vs PR p = 0.0039, cPD vs CR p = 0.034; NLR: cPD vs PD p = 0.00093, cPD vs SD p = 0.00042, cPD vs PR p = 0.0075, cPD vs CR p = 0.025. **d**, Proportion of explained variance (R^2^) of microbial composition by each available clinical and technical metadata variable. Calculated using PERMANOVA on inter-sample Aitchison distance (9999 permutations). Metadata variables coloured by category (blood, exposome, patient, technical or tumour). PERMANOVA p-values summarized (*: p < 0.05). Exact p-values available in Supplementary Table 6 (‘ca209-538_permanova’). **e**, Analysis of baseline microbial variance by moving PFS cut-off (1-monthly intervals, from 1-24 months). Top panel show microbial variance between groups formed by cut-off (inverse PERMANOVA p-value, 999 permutations) using Aitchison distance. Bottom panel shows proportion of progression-free-survivors at respective threshold (that is the proportion in each group). Dashed line with * indicates p = 0.05 threshold. Exact p-values available in Supplementary Tables 7 (‘moving_pfs_permanova’). Acronyms: UGB = upper gastrointestinal & biliary, NEN = neuro-endocrine neoplasms, GYN = gynaecological, PFS = progression-free survival, OS = overall survival, BOR = best overall response, CR = complete response, PR = partial response, SD = stable disease, PD = progressive disease, cPD = clinical progressive disease, chemo = chemotherapy, PPI = proton-pump inhibitor, BMI = body-mass index, LDH = lactate dehydrogenase, NLR = neutrophil:lymphocyte ratio, ECOG = eastern cooperative oncology group, PERMANOVA = permutational multivariate analysis of variance.

The clinical efficacy and safety outcomes for subgroups from CA209-538 have been published previously^23–26^. As expected, overall survival (OS) significantly differed by histology (Extended Data Fig. 1a); however, progression-free survival (PFS) was more consistent (Extended Data Fig. 1b). Notably, the percentage of patients with an objective response (complete response (CR) or partial response (PR)) was remarkably stable across histological cohorts (24–25%) (Fig. 1b), with the Response Evaluation Criteria in Solid Tumors (RECIST) 1.1 best overall response (BOR) being strongly associated with PFS and OS (Fig. 1c,d). Using univariable statistical testing, we found a strong positive monotonic association between albumin and BOR (Kendall *P* = 0.0056) and a negative monotonic association between neutrophil-to-lymphocyte ratio (NLR) and BOR (Kendall *P* = 0.0033) (Extended Data Fig. 1c). This was particularly driven by patients with rapid clinical progression (clinical PD (cPD)) having significantly lower albumin and higher NLR, both responses to inflammation shown to be strongly prognostic across cancer types and treatment settings^27,28^.

### Microbiome profiling of baseline fecal samples

To understand the composition of patient gut microbiomes, we performed deep shotgun metagenomic sequencing of the 106 available baseline fecal samples (median 20.4 million paired-end reads per sample). For precise taxonomic quantification, we used a genome-resolved approach of first assembling a study-specific strain reference database using metagenome-assembled genomes (MAGs), supplemented with relevant Genome Taxonomy Database (GTDB) species reference genomes (SRGs) (Methods). Ultimately, this database included 1,397 strain genomes covering 904 known species and additionally included 34 ‘new’ strains that could be taxonomically classified only to the genus level. The Bowtie 2 alignment rates to our tailored strain reference library were high (median 88.4%), with a median of 10.2 million mapped paired-end reads (50%) passing stringent quality control and used for precise strain quantification (Supplementary Fig. 1 and Methods).

We first evaluated whether there were gross compositional differences based on the patients’ BOR. Notably, we found a positive monotonic association between BOR and the fecal Shannon diversity index, a common alpha diversity metric (Fig. 1e). Associations between alpha diversity and cancer patient outcomes have been found in the setting of patients receiving hematopoietic cell transplant^29^ or cervical cancer chemoradiation^30^ but not in anti-PD-1 recipients with metastatic melanoma^16,18^; thus, such associations may be treatment regimen specific. We then assessed intersample beta diversity using the Aitchison distance and also found gross microbial compositional differences by BOR group (permutational multivariate analysis of variance (PERMANOVA) *P* = 0.0319) (Fig. 1f). Indeed, among the 23 pretreatment clinical and technical metadata tested, BOR group was the metadata variable explaining the most microbial variance (Extended Data Fig. 1d). By contrast, patient PFS at 12 months (PFS12) or OS at 12 months was associated with little microbial variance. A PERMANOVA of baseline microbial variance versus a moving PFS threshold revealed a peak association at <4 months (Extended Data Fig. 1e), indicating that, in our cohort, patients with rapid progression had the most distinct gross baseline microbial compositions.

### Strain–response signatures are valid across cancer types

**Extended Data Fig. 2:**
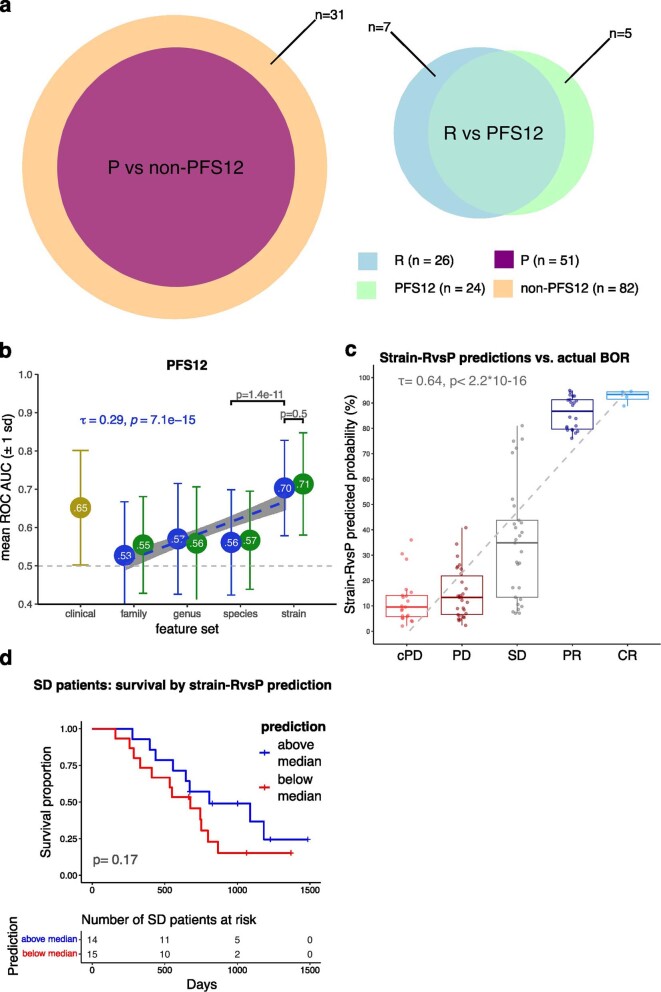
Sensitivity analyses of gut microbial strain-efficacy classifiers. **a**, Comparison of the RvsP and PFS12 binary endpoints. Venn diagrams show the overlap between the ‘negative and ‘positive’ outcome populations (P/non-PFS12 and R/PFS12 respectively). Size of circles (area) in proportion to population size, with set differences labelled. **b**, AUC scores for the best iteration of PFS12 classifiers for each feature-set combination during 20-repeated 5-fold cross-validation (100 folds each): clinical (yellow), microbiome (blue) and combined (green), at different taxonomic resolutions. Mean (circle) and standard deviation (error bars) over the 100 folds. Linear model line-of-best-fit for AUC score and taxonomic rank of microbiome-only feature sets (with shaded 95% confidence interval) superimposed. Kendall *τ* and p-value for association between AUC score and taxonomic rank of microbiome-only feature sets printed. Mann-Whitney U p-value for comparison of AUCs of specific pairwise feature-sets (depicted by callipers) printed. **c**, Patient’s predicted RvsP (using strain-RvsP RF classifiers trained on the full evaluable cohort) vs. actual BOR outcome (cPD n = 21, PD n = 30, SD n = 29, PR n = 22, CR n = 4). Boxplot centre line= median; box limits= upper and lower quartiles; whiskers= 1.5x interquartile range. Kendall rank correlation *τ* and p-value for association between predicted RvsP and actual BOR printed. **d**, Kaplan-Meier overall survival curves for those patients with a best overall response (BOR) of stable disease (n = 29), stratified by those with above median (blue) and below median (red) strain-RvsP RF classifier predictions. Bottom panel shows number of patients at risk at each marked interval. P-value by log-rank test printed. Acronyms: P= progressors (RECIST progressive disease (PD) or clinical progressive disease (cPD)), R= responders (RECIST complete response (CR) or partial response (PR)), GYN= gynaecological, NEN= neuro-endocrine neoplasm, UGB= upper gastrointestinal & biliary, ROC= receiver operating characteristic, AUC= area under curve, OS= overall survival, SD= stable disease, RvsP= response versus progression, cPD= clinical progressive disease, PD= progressive disease, SD= stable disease, PR= partial response, CR = complete response.

Given the gross compositional differences, we hypothesized that specific strains may allow for prediction of CICB efficacy in our cohort. We assessed objective response versus progression (RvsP), defined as a RECIST BOR of CR or PR versus PD or cPD, as our primary endpoint. In doing so, we excluded patients with a BOR of stable disease (SD) (*n* = 29), given its ambiguity in a pan-cancer cohort, in which it may represent disease control or simply indolent cancer behavior. As a sensitivity analysis, we also evaluated PFS12, with responders and those with PFS12 largely overlapping given the durability of CICB efficacy (Extended Data Fig. 2a).

**Fig. 2:**
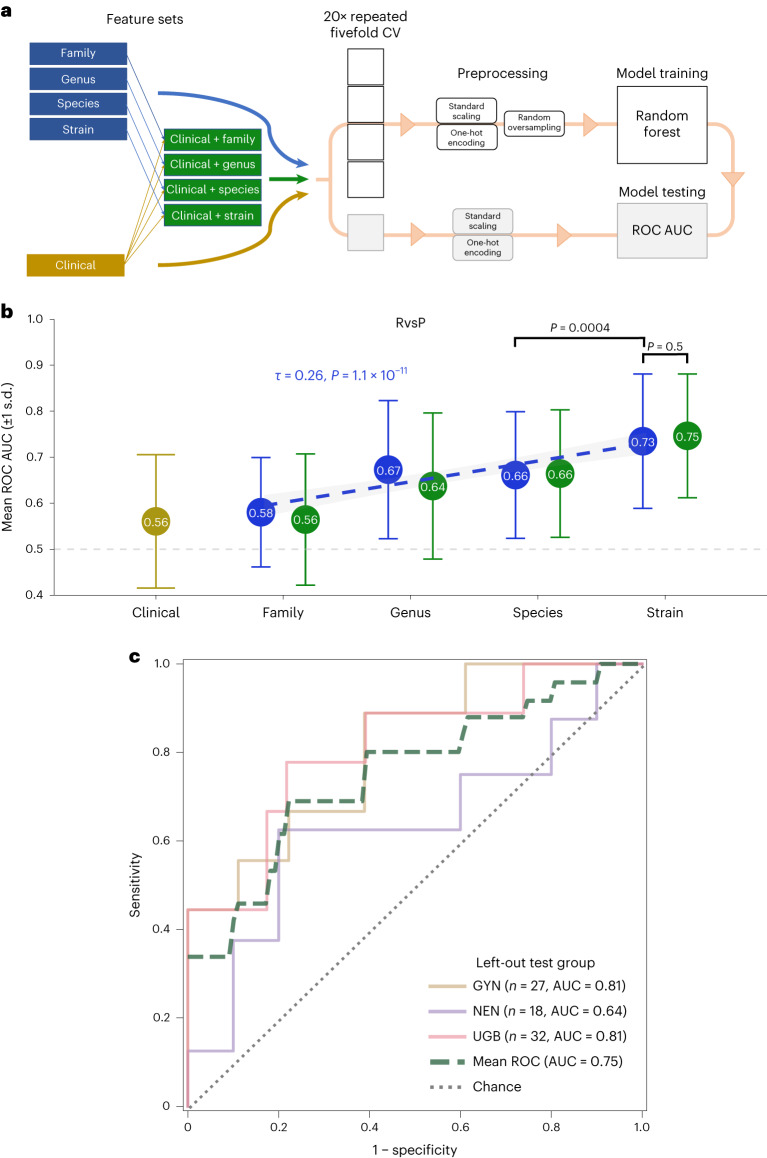
Strain-resolution gut microbial signatures outperform clinical predictors and cross-validate across tumor histology types. **a**, Schematic of the supervised ML framework. Input features (clinical, microbiome or combined) and the target variables (RvsP or PFS12) were split into five folds (four training folds, one testing fold). The process was repeated 20 times per iteration, with the AUC score used to select the best hyperparameters. CV, cross-validation. **b**, AUC scores for the best iteration of RvsP classifiers for each feature set combination during 20 times repeated fivefold cross-validation (100 folds each): clinical (yellow), microbiome (blue) and combined (green), at different taxonomic resolutions. Data represent the mean (circle) and s.d. (error bars) over the 100 folds. The linear model (line of best fit) for the AUC score and taxonomic rank of microbiome-only feature sets (with shaded 95% confidence interval) is superimposed. Kendall *τ* and *P* values for the association between the AUC score and taxonomic rank of microbiome-only feature sets are indicated. The Mann–Whitney *U* test *P* value for comparing the AUCs of specific pairwise feature sets (depicted by calipers) is also indicated. **c**, ROC curves for the strain–RvsP classifiers retrained using leave-one-histology-cohort-out cross-validation. Model training and testing were repeated 100 times, with predictions averaged to account for model stochasticity.

We used a supervised machine learning (ML) workflow (Fig. 2a). As input features (predictors), we tested the 15 potentially relevant clinical factors (Methods) and the microbial factors (centered log ratio (CLR)-transformed strain abundances) separately and combined to assess their relative and synergistic performance, respectively. In addition to strain-level rank, we tested microbial abundances aggregated to higher taxonomic ranks (species, genus and family levels) to determine the influence of taxonomic resolution on predictive performance. For each feature set, we performed a thorough random hyperparameter search across 1,000 iterations of a 20 times repeated fivefold cross-validation (Methods). For predictions, we used a random forest (RF) classifier, previously shown to generally outperform other classical ML algorithms for microbiome–host predictions^31^.

Interestingly, we found that clinical factors alone were poorly predictive of RvsP (mean receiver operating characteristic (ROC) area under the curve (AUC) = 0.56) (Fig. 2b). This was despite the previously observed relationship between low blood albumin, high NLR and cPD, suggesting that these factors are more useful for delineating patients with the worst prognosis rather than distinguishing responders and nonresponders. Furthermore, it affirms the current difficulty of predicting clinical activity using routinely available factors and emphasizes the need for further technical innovation. In contrast, clinical factors were more predictive of PFS12 (AUC = 0.65; Extended Data Fig. 2b), inferring that these are more prognostic markers than predictors of antitumor activity.

When microbiome features were used, there was a positive monotonic association between the mean AUC score and taxonomic resolution for both endpoints (increasing from family to strain level) (Kendall *P* = 1.1 × 10^−11^ for RvsP, *P* = 7.1 × 10^−15^ for PFS12). In particular, strain-resolved abundances provided the best predictive performance (AUC = 0.73 for RvsP, AUC = 0.70 for PFS12), significantly outperforming the more common species-level abundances. Consistent with their poor standalone performance, clinical factors failed to augment microbiome predictors. Overall, these data suggest that microbial abundances, especially at strain-level resolution, are more valuable in predicting tumor response or landmark PFS than higher taxonomic aggregations or clinical features.

We subsequently focused on strain–RvsP classifiers, given their superior performance and larger incremental benefit over routine clinical factors. We were particularly interested in assessing the concordance of strain–RvsP predictions from the entire cohort (*n* = 77 evaluable) with actual patient BOR outcomes. Notably, despite being trained on binary RvsP, the predicted probabilities of patients were correctly ranked by their actual BOR category (Kendall *P* < 2.2 × 10^−16^), including (on average) central predictions for the SD group that were ‘unseen’ during model training (*n* = 29) (Extended Data Fig. 2c). Intrigued, we assessed whether RvsP predictions could distinguish a ‘better’ or ‘worse’ SD group. Indeed, we found a nonsignificant improvement in the OS of patients with SD with an above-median RvsP prediction, although this analysis was likely underpowered (log-rank *P* = 0.17; Extended Data Fig. 2d).

Finally, a key priority was to identify whether microbial signatures are tumor agnostic; that is, whether they generalize from one distinct tumor type to another. As our study naturally has three distinct cancer cohorts (GYN, NEN and UGB), we performed a leave-one-group-out cross-validation (training strain–RvsP classifiers using two groups and then testing on the left-out group). Notably, the mean AUC of the left-out group was consistently superior to that of a random model (overall mean AUC = 0.75) (Fig. 2c). Although the small sample size limits its interpretability, the particularly good performance for the UGB and GYN groups may reflect the specific relevance of the gut microbiome in these cancers.

Our ML analysis of our discovery cohort demonstrates that strain-level gut microbial predictors of CICB response may be relatively robust across diverse cancer types and are superior to ML predictors built using routine clinically available data. Furthermore, predictions trained on binary RvsP appear to capture the RECIST BOR biologically and may have utility for predicting the durability of SD.

### *Faecalibacterium* strains are positively implicated

**Extended Data Fig. 3:**
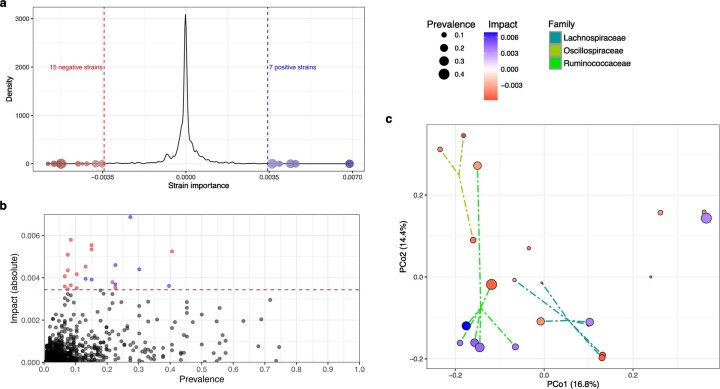
Identification and metabolic-potential profiling of the top 22 response predictive strains. **a**, Kernel density plot of impact (feature importance) of strains in the strain-RvsP classifier. The top 22 strains with absolute impact within half maximal value shown (coloured by importance, and size by prevalence). **b**, Strain impact (absolute) versus prevalence in the CA209-538 cohort. Top 22 strains coloured (blue and red for positive and negative associations with response, respectively), with importance threshold depicted (red dashed line). **c**, Plot of principal coordinate 1 vs 2 using Jaccard dissimilarity of metabolic pathway presence/absence for top 22 strain genomes. Points (individual strains) coloured by impact on RvsP, and size by prevalence. Acronyms: PCo= principle coordinate.

We next sought to understand which features (strain abundances) were most important in driving the strain–RvsP model predictions. To do this, we used the SHapley Additive exPlanations (SHAP) ‘TreeExplainer’ algorithm^32^ (Methods). We first noted that, although most strains contributed little to predictions, a few were disproportionately important (Extended Data Fig. 3a). Twenty-two strains were within half as impactful as the most important strain (a strain of *Faecalibacterium* sp900539885, an uncultured species), which we opted to focus on subsequently. Interestingly, these strains were neither rare (<5% prevalent) nor core (>50% prevalent) taxa within our cohort (Extended Data Fig. 3b).

**Fig. 3:**
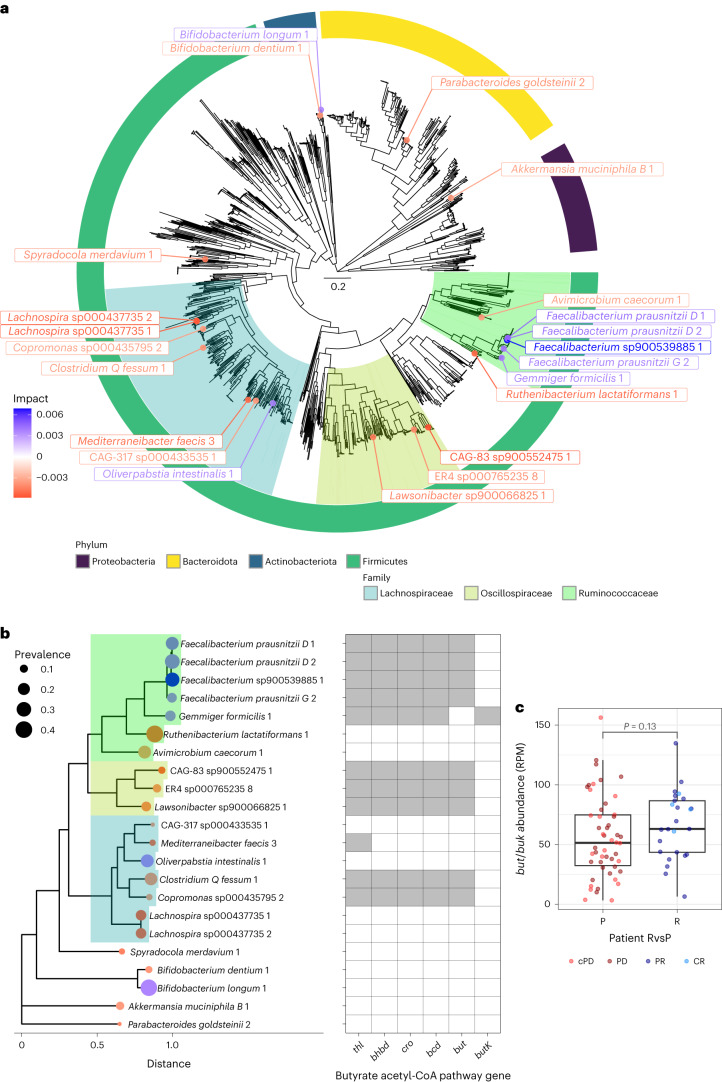
Firmicutes bacteria dominate the gut microbiome strain–response signature. **a**, Phylogenetic tree of bacterial strains in our custom reference library (*n* = 1,391 strains, excluding *n* = 6 archaea), highlighting the top 22 strains (labels are colored by impact (that is, feature importance) on RvsP predictions). Four main phyla are shown by the colored ring, with the Ruminococcaceae, Oscillospiraceae and Lachnospiraceae families highlighted. The scale for phylogenetic distance is shown in the center of the tree. **b**, Phylogenetic tree of the top 22 strains, with the tips colored by strain impact and sized by strain prevalence. The adjacent heat map depicts the presence or absence of genes within the primary butyrate-producing (acetyl-CoA) pathway. Full enzyme (encoding gene) names: acetyl-CoA acetyltransferase (*thl*), β-hydroxybutyryl-CoA dehydrogenase (*bhbd*), crotonase (*cro*), butyryl-CoA dehydrogenase (*bcd*), and the alternative terminal enzymes butyryl-CoA:acetate CoA transferase (*but*) and butyrate kinase (*buk*). **c**, Boxplots of the sample-wise abundance of butyrate acetyl-CoA terminal enzymes (*but* + *buk*), split by patient response (progression (P) *n* = 51, response (R) *n* = 26). Boxplot center line indicates the median; box limits indicate the upper and lower quartiles; and whiskers indicate 1.5× the interquartile range. Abundance is normalized as reads per million (RPM). *P* value by the Mann–Whitney *U* test is indicated.

To visualize the phylogenetic relationships of these ‘top 22’ strains in the context of all study-specific bacterial strains, we constructed an approximately maximum-likelihood phylogenetic tree using the GTDB toolkit (GTDB-tk) (Methods and Fig. 3a). This demonstrated that 20 of the 22 strains were gram positives, with most (18 of 20) belonging to the Firmicutes (Bacillota) phylum. The most ‘beneficial’ strains (that is, higher strain abundances shifted predictions toward ‘response’) clustered in one clade of the Ruminococcaceae family, with four being strains within the *Faecalibacterium* genus. Until recently, the National Center for Biotechnology Information taxonomy database recognized only one species within the genus *Faecalibacterium* (*F. prausnitzii*)^33^, and its fecal abundance has been associated with good general health^34^ and response to anti-PD-1 monotherapy in patients with melanoma^16^ or hepatobiliary cancers^15^. However, more recent analyses have revealed considerable phylogenetic and functional diversity within the *F. prausnitzii* species complex^22^. In keeping with this, at the 98% genomic identity threshold, our custom strain reference library included *n* = 35 distinct *Faecalibacterium* strains (from *n* = 13 distinct species), with the most important (and prevalent) clustering near the *F. prausnitzii D* phylogenetic clade (Supplementary Fig. 2).

Conversely, 15 of the 22 strains appeared to have a negative association with response in our discovery cohort. As before, most were Firmicutes, with 6, 3 and 2 (of the 15) strains belonging to the Lachnospiraceae, Oscillospiraceae and Ruminococcaceae families, respectively. Notably, eight of these strains belonged to thus far uncultivated (and thus unnamed) species. The remaining four ‘negative’ strains belonged to the species *Bifidobacterium dentium*, *A. muciniphila* *B* and *Spyradocola merdavium*. It should be noted that *A. muciniphila B* is a distinct species from *A. muciniphila*; although the latter was positively implicated in anti-PD-1 efficacy in NSCLC^13^ (also positive in our study but not within the top 22 strains), recent analyses have revealed that it is phylogenetically and phenotypically distinct from *A. muciniphila B* (known as *Akkermansia* SGB9228 by MetaPhlAn4 taxonomy)^21^. The juxtaposition of *Bifidobacterium longum* 1 and *B. dentium* 1 as positive and negative, respectively, also highlights how closely related taxa can have discordant relationships with host phenotypes. Indeed, while the species *B. longum* has been linked to positive health outcomes, such as protection from inflammatory bowel disease^35^, protection from childhood malnutrition^36^, and anti-PD-1 responses^37^, *B. dentium* is a known oral opportunistic pathogen linked to tooth decay^38^.

We next aimed to interrogate the genomes of the top 22 strains to understand functional potentials that may underpin their strong (positive and negative) response associations. We first evaluated them for virulence factors and found that they harbored none, suggesting that even the negative strains are not prototypical ‘pathogens’. To look more broadly at strain functional potential, we queried the presence or absence of metabolic pathways using the tool gapseq (Methods). As expected, we observed clustering of metabolic potential by phylogeny; however, the two negative Ruminococcaceae (strains of the *Ruthenibacterium lactatiformans* and *Avimicrobium caecorum* species) were quite distinct from the five ‘positive’ strains (Extended Data Fig. 3c).

We hypothesized that specific metabolic functions may distinguish these negative and positive Ruminococcaceae. One metabolite of particular interest was butyrate, given that it has been implicated in anticancer cytotoxic T cell activation preclinically^39–41^, and fecal butyrate has been positively associated with ICB efficacy in clinical cohorts^42,43^. Additionally, although butyrate-producing potential has previously been broadly ascribed to Ruminococacceae, more recent analyses have revealed marked strain-level variation within this family^44^. Indeed, the acetyl-CoA butyrate pathway (which dominates among Firmicutes bacteria) was complete in all (five of five) positive but no negative (none of two) top 22 Ruminococacceae (Fig. 3b). In contrast, taking a ‘strain-agnostic’ approach of quantifying the abundance of the acetyl-CoA butyrate terminal enzymes (*but* + *buk*) in metagenomic samples did not reveal a significant enrichment in responders (Fig. 3c), highlighting the need for strain-aware approaches to develop context-specific functional hypotheses.

### Microbial signatures may be ICB regimen specific

**Fig. 4:**
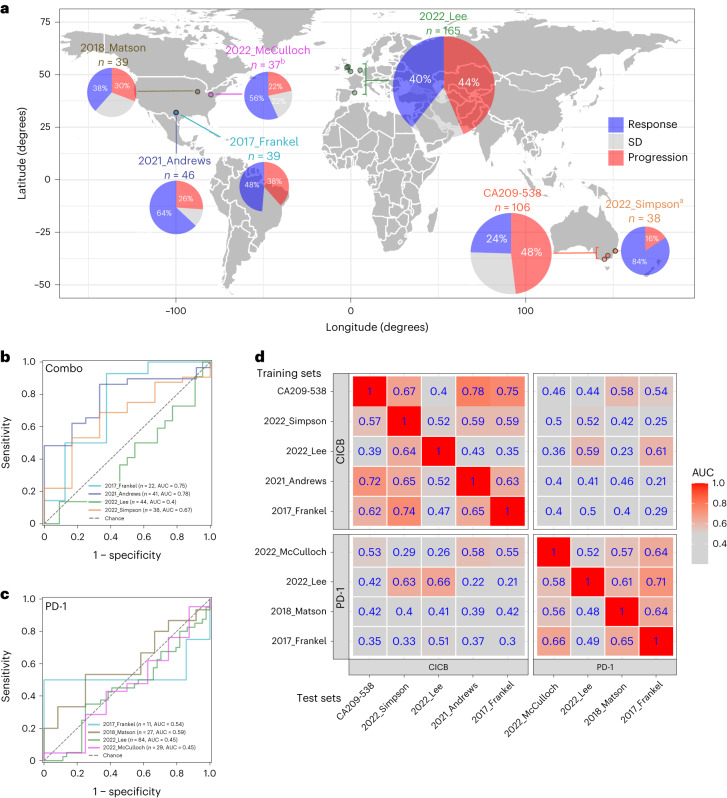
Meta-analysis reveals that gut microbiome strain–response signatures are ICB regimen specific. **a**, World map showing the studies included in our meta-analysis. Bordered circles depict the coordinates of recruiting sites (cities). Pie charts depict the proportion of patients with tumor response, progression or SD. The area of the pie charts depicts the sample size. ^a^2022_Simpson studied neoadjuvant ipilimumab + nivolumab for stage III melanoma and thus used pathological response criteria (International Neoadjuvant Melanoma Consortium criteria); all other studies used the RECIST 1.1 criteria. ^b^For this study, only the subset of patients (*n* = 37) with stool collected within 15 days of the start of ICB therapy was included in the meta-analysis. **b**, ROC curve of strain–RvsP classifiers trained on the discovery cohort (CA209-538) and tested on external CICB cohorts separately. **c**, ROC curve of strain–RvsP classifiers trained on the discovery cohort (CA209-538) and tested on external anti-PD-1 monotherapy cohorts separately. **d**, Heat map denoting the AUC scores for strain–RvsP classifiers trained on one dataset (column) and tested on another (rows). Panels are faceted by ICB regimen (CICB or anti-PD-1 monotherapy).

**Extended Data Fig. 4:**
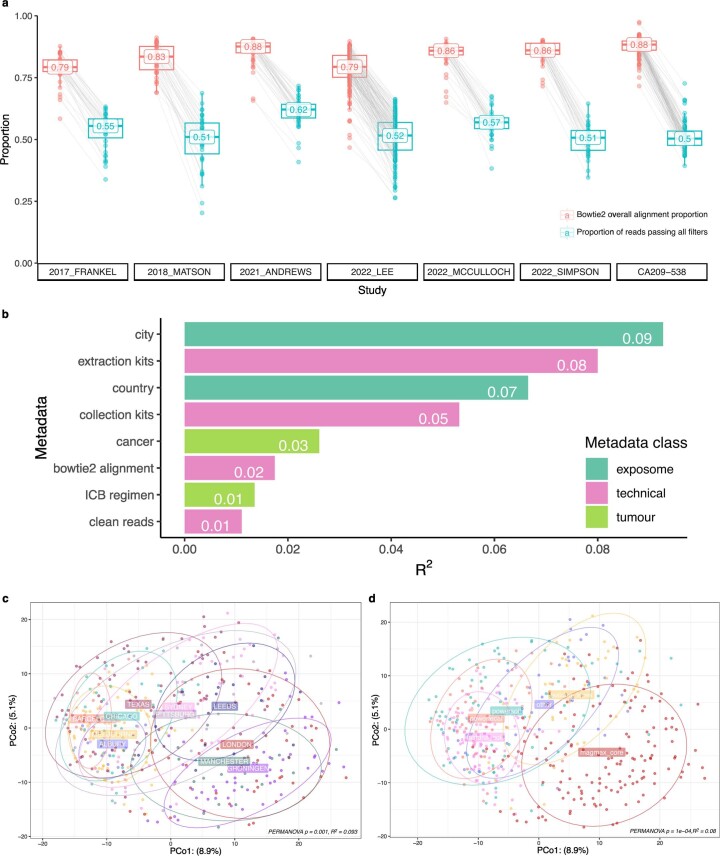
Heterogeneity of baseline gut microbial compositions across meta-analysis cohorts. **a**, Proportion of quality-controlled paired-end reads aligned by Bowtie 2 (red), and ultimately used for abundance estimation after stringent filtering (cyan). Organised by study (2017_FRANKEL n = 39, 2018_MATSON n = 39, 2021_ANDREWS n = 46, LEE n = 165, 2022_MCCULLOCH n = 37, 2022_ 2022_SIMPSON n = 38, CA209-538 n = 106). Boxplot central line= median, box limits= upper and lower quartiles, and whiskers= 1.5x interquartile range. Median printed within each boxplot. **b**, Proportion of explained variance (R^2^) of microbial composition by metadata variables (grouped into ‘exposome’, ‘technical’ and ‘tumour’ categories. R^2^ values (printed on bar) calculated using PERMANOVA (9999 permutations). **c**, PCA plot of samples by CLR-transformed abundances (Aitchison’s distance), with points coloured by sample city (the variable explaining the most variance). Ellipses depict 0.8 of each group’s multivariate t-distribution. PERMANOVA p-value and R^2^ using 9999 permutations printed. **d**, PCA plot of samples by CLR-transformed abundances (Aitchison’s distance), with points coloured by extraction kit (the variable explaining the second-most variance). Ellipses depict 0.8 of each group’s multivariate t-distribution. PERMANOVA p-value and R^2^ using 9999 permutations printed. Acronyms: ICB= immune checkpoint blockade, PCo= principle coordinate.

To evaluate the external generalizability of our strain–RvsP signature, we reanalyzed all comparable shotgun metagenomic cohorts (Methods and Supplementary Fig. 3). We included cohorts that analyzed baseline (±15 days of ICB commencement) fecal samples, performed Illumina paired-end shotgun metagenomic sequencing, and provided either RECIST BOR (five studies) or pathological response (one study) metadata. Including our discovery cohort (CA209-538 cohort), the seven studies recruited participants from 11 cities across five countries (United States, United Kingdom, Netherlands, Spain and Australia) (Fig. 4a) and represent *n* = 470 total patients (*n* = 383 after excluding patients with a BOR of SD). Quality-controlled reads were mapped to the same reference library to estimate abundances for the same 1,397 strains. Although we were mindful that the reference library derived from the CA209-538 cohort might not represent all bacterial strains in external studies, we were reassured by both the high overall Bowtie 2 alignment rates (median 79.2–87.6% across external studies) and the high proportion of quality-controlled reads used for abundance estimation after stringent filtering (median 50.7–62.1% across external studies) (Extended Data Fig. 4a).

A summary of the key characteristics of the included studies is provided in Table 2. Given that all external studies evaluated patients with melanoma, known to be particularly amenable to ICB, it is not surprising that their objective response rates trended higher than those in our study that evaluated patients with diverse rare cancer types (38–84% versus 25%; Fig. 4a). This highlights that tumor type is an important variable in determining ICB response but does not preclude the existence of universal gut microbiota that may enhance or detract from an individual’s likelihood of showing an antitumor ICB response.

**Table 2:** Characteristics of studies included in the meta-analysis

Characteristics	Study						
	CA209-538 (*n* = 106)	2022_Simpson^43^ (*n* = 38)	2021_McCulloch^19^ (*n* = 37)^a^	2022_Lee^18^ (*n* = 165)	2021_Andrews^59^ (*n* = 46)	2018_Matson^37^ (*n* = 39)	2017_Frankel^60^ (*n* = 39)
Country	Australia	Australia	USA	UK, Netherlands, Spain	USA	USA	USA
Cancer type (%)	UGB (36%), GYN (34%), NEN (30%)	MEL (100%)	MEL (100%)	MEL (100%)	MEL (100%)	MEL (100%)	MEL (100%)
ICB regimen (%)	CICB (100%)	CICB (100%)	Anti-PD-1 (100%)	CICB (33%), anti-PD-1 (61%), anti-CTLA-4 (7%)	CICB (100%)	Anti-PD-1 (100%)	CICB (62%), anti-PD-1 (36%), anti-CTLA-4 (3%)
Response criteria	RECIST 1.1	Pathological (INMC)	RECIST 1.1	RECIST 1.1	RECIST 1.1	RECIST 1.1	RECIST 1.1
Response							
CR	3.8%	PathR: 84%	5.4%	13%	11%	5.1%	13%
PR	21%		51%	26%	52%	33%	36%
SD	27%		22%	17%	11%	31%	13%
PD	28%	Non-pathR: 16%	22%	42%	26%	31%	39%
cPD	20%		0%	1.8%	0%	0%	0%
Stool collection kit	OMR-200	EasySampler	EasySampler	LO—TF kits, MA—plain tube, NL—plain tube, LD—OMR-200, BL—OMR-200	OMR-200	EasySampler	NR
DNA extraction kit	FastDNA soil	FastDNA feces	PowerSoil	LO—TF MagMAX, MA—TF MagMAX, LD—TF MagMAX, NL—TF MagMAX, BL—PowerFecal	PowerSoil	PowerFecal	Other
Sequencer (bases per read)	NovaSeq (2 × 151)	NovaSeq (2 × 151)	NovaSeq (2 × 151)	NovaSeq (2 × 151)	NextSeq (2 × 151)	NextSeq (2 × 151)	HiSeq (2 × 100)
Clean PE reads (millions)							
Minimum	9.10	5.05	2.72	4.38	12.8	19.3	18.1
Median	20.4	22.5	30.5	20.7	40.0	35.6	45.4
Maximum	53.8	34.5	72.2	104	69.8	77.7	59.9

The clinical and technical characteristics of the studies included in the meta-analysis are summarized. Published studies are denoted by ‘year_author’.

USA, United States of America; UK, United Kingdom; MEL, melanoma; INMC, International Neoadjuvant Melanoma Consortium; LO, London; MA, Manchester; NL, Netherlands; LD, Leeds; BL, Barcelona; TF, Thermo Fisher Scientific; PathR, pathologic response; NR, not reported; PE, paired-end.

^a^Of the original ‘Pittsburgh early cohort’ (*n* = 63), *n* = 37 had their analyzed stool sample collected between day −15 and day 15 of starting ICB and were therefore deemed eligible.

A PERMANOVA of individual metadata variables revealed that the leading sources of microbial variance across the meta-cohort were study site (city) (9.3%) and DNA extraction kit (8.0%) (Extended Data Fig. 4b). However, these two factors were also strongly associated with one another (chi-squared test *P* < 2.2 × 10^−16^), with distinct studies recruiting participants from specific cities but also using distinct DNA extraction kits (Extended Data Fig. 4c,d). Although it would be desirable to ‘correct’ for DNA extraction kit (which has a well-described influence on downstream microbial quantifications^45^), this would likely also mitigate the true biological variance caused by patient geography^46^ (which is important when evaluating the cross-country validity of a biomarker). Furthermore, a recent reanalysis of an intratumoral microbiome meta-analysis raised concerns that statistical batch correction may artificially inflate cross-cohort predictions due to data leakage^47^. Therefore, to evaluate the performance of our strain–RvsP classifier as robustly as possible, we opted not to adjust abundances beyond CLR transformation.

Given their distinct mechanisms of action, we were particularly interested in differentially evaluating performance on CICB and anti-PD-1 monotherapy cohorts. Of the six external studies, two comprised only anti-PD-1 recipients, two comprised only CICB recipients and two comprised both and were split based on regimen, creating eight external validation cohorts (four CICB, four anti-PD-1). Notably, there was a marked difference in the performance of the CA209-538 strain–RvsP signature between these groups, with overall modest external generalizability to CICB cohorts (mean AUC = 0.65; Fig. 4b) but no generalizability to anti-PD-1 cohorts (mean AUC = 0.51; Fig. 4c).

Intrigued, we sought to use our meta-cohort to evaluate whether this difference could also be seen more generally. We thus trained and tested strain–RvsP RF classifiers using all strain abundances and every pairwise combination of cohorts (nine cohorts, keeping 2017_Frankel and 2022_Lee split by ICB regimen) and evaluated AUCs. Consistent with our previous observation, we found that the predictive performance was better when training and testing on ‘concordant’ cohorts—that is, when the training and test cohorts received the same ICB regimen—rather than ‘discordant’ cohorts (Fig. 4d). Importantly, this was also true for strain–RvsP signatures trained on anti-PD-1 monotherapy cohorts. Taken together, the results showed a significant improvement in the cross-study strain–RvsP predictive performance in concordant rather than discordant regimen cohorts (Mann–Whitney *U* test *P* = 2.8 × 10^−7^).

## Discussion

In this study, we used strain-resolved metagenomic classification to discover a signature of 22 gut microbial strains associated with response to combination ipilimumab (anti-CTLA-4) plus nivolumab (anti-PD-1) in a phase 2 trial cohort of Australian patients with diverse rare cancers (*n* = 106). To our knowledge, this represents the largest gut microbiome study of patients treated with CICB published to date. Using supervised ML, we demonstrate the value that precise, strain-level gut microbial quantifications provide in predicting clinical response or PFS12, exceeding the value of routinely available clinical information or that of higher taxonomic rank abundances. Furthermore, we show the external generalizability of strain-level response signatures across cancer histology types and countries, both within the trial (comparing across the predetermined histology cohorts) and externally (to metastatic melanoma cohorts from other industrialized countries). This was despite a strong heterogeneity in microbiome composition across cohorts, likely influenced by divergent fecal collection and DNA extraction methods. Finally, we observed a striking difference in the cross-study performance of response classifiers trained and tested on concordant versus discordant ICB cohorts, implying that different microbial relationships likely underlie these distinct treatment regimens.

Given the success of combination anti-PD-1 and anti-CTLA-4 ICB across diverse cancers, there is great interest in defining tumor-agnostic pretreatment biomarkers, including through using gut microbial abundance signatures. A recent review by Thomas et al.^20^ defined cross-cancer ICB response (‘Gut OncoMicrobiome Signature’) implemented using species-level abundances. This study differs, first, in using strain-level signatures and, second, by deliberately splitting cohorts into those receiving anti-PD-1 monotherapy and those receiving anti-PD-1 plus anti-CTLA-4 CICB. Of note, although Thomas et al. found good left-out performance for the exclusively anti-PD-1-treated NSCLC and renal cell carcinoma cohorts, performance was poor among left-out melanoma cohorts, potentially due to patients receiving monotherapy and those receiving CICB being admixed.

Although the external performance of the CA209-538 strain–response signature fell short of what is required for clinical use, its performance was remarkably better in CICB (AUC = 0.67, 0.40, 0.78 and 0.75) than anti-PD-1 (AUC = 0.46, 0.44, 0.58 and 0.54) melanoma cohorts from other industrialized countries. Consistent with this, strain–response signatures trained on external cohorts were also superior when tested on concordant rather than discordant regimen cohorts. Thus, we believe that this work makes a strong case for distinct microbial consortia underpinning response or nonresponse to each regimen. This is biologically plausible, given that we know that CICB has a distinct mechanism of action compared to anti-PD-1 monotherapy^48^ and distinct baseline tumor immune microenvironment signatures^49^. Furthermore, the addition of anti-CTLA-4 has a profound effect on gut barrier permeability^50,51^, potentially changing the influence of the gut microbiome on ICB response. Nevertheless, the poor generalizability of the CA209-538 strain–RvsP signature to anti-PD-1 cohorts is still intriguing, given the similarity in key positive strains and those species or genera previously associated with response. For example, *Faecalibacterium* has been linked to the efficacy of anti-PD-1 monotherapy in patients with melanoma^16^ or hepatobiliary^15^ cancers, and *B. longum* has been linked to anti-PD-1 efficacy in patients with melanoma^37^ and NSCLC^52^. Therefore, we postulate that the distinction may lie in the negative taxa, with many of the top negative strains in our signature being members of the Lachnospiraceae family (previously broadly associated with anti-PD-1 response in melanoma cohorts^19^). This is also conceptually consistent with the observation of more discrepancies in the pretreatment tumor immunotranscriptomic landscape of anti-PD-1 and CICB nonresponders compared to responders^49^.

This work has several limitations that should be addressed in the future. First, despite our relatively large discovery cohort and meta-analysis, the individual cohort and total sample sizes are still small, limiting the statistical power of our signature. Future meta-analyses will benefit from larger, more geographically diverse cohorts, ideally with standardized, best-practice approaches to fecal collection and DNA extraction methods^45^. Moreover, although we used a state-of-the-art bioinformatics pipeline to generate and quality control MAGs to represent study-specific strains (many of which are new or uncultivated), they still potentially harbor errors (such as fragmentation, assembly breaks and contamination)^53^. Although not possible due to the collection medium used in this study, our group has previously demonstrated large-scale fecal strain-culturing methods^54^, which, when coupled with whole-genome sequencing, have allowed us to build comprehensive, context-specific genome reference libraries that improve the accuracy of reference-based metagenomic taxonomic classification^55^. Finally, such patient-specific culturing is necessary to perform in vitro and in vivo testing of microbial strains or consortia to derive precise mechanistic insights into their associations with response or nonresponse to ICB and to confirm the direction of causality.

Until then, we believe that this work provides a number of readily implementable insights to help future research and development in this field. First, it highlights the added value of strain resolution in developing gut microbial ICB biomarkers. There is now ample evidence that intraspecies variation of gut microbiota can substantially change their effect on hosts, first described for enteric pathogens (for example, *Escherichia coli*^56^) but more recently demonstrated for immunomodulatory commensals^57,58^, providing further conceptual support for this notion. Second, it suggests that strain signatures may be generalizable across cancer types and geographic locations, supporting investment in developing ‘pan-cancer’ gut microbial diagnostics and/or therapeutic ICB adjuncts. Lastly, the distinct performance of CICB and anti-PD-1 gut microbial signatures suggests that we should disaggregate these regimens in future analyses to define the relationships between gut microbiota and ICB more precisely in a regimen-specific fashion and, eventually, to use this information in personalizing the care of cancer patients.

## Methods

### CA209-538: clinical trial procedures

CA209-538, titled *‘*A phase 2 trial of ipilimumab and nivolumab for the treatment of rare cancers*’*, is an investigator-initiated, prospective, multicenter, single-arm clinical trial (NCT02923934). The study was approved by the Austin Health (Melbourne, Australia) Human Research Ethics Committee (approval: HREC/16/Austin/152).

Between October 2017 and February 2020, 120 adult patients with rare cancers were recruited across five sites in southeastern Australia (Austin Health, Peter MacCallum Cancer Centre, Monash Health, Blacktown Hospital and Albury Wodonga Health/Border Medical Oncology). Patients were recruited into three prespecified ‘histology cohorts’ of approximately equal sizes: (1) UGB, comprising cholangiocarcinomas, gallbladder cancers, duodenal cancers and gastrointestinal stromal tumors; (2) NEN, including neuroendocrine tumors or carcinoma of any primary organ (except small cell lung carcinoma) or adrenocortical carcinoma; and (3) GYN, comprising diverse histologies including carcinosarcoma, low-grade serous carcinoma and clear-cell carcinoma of gynecological organs.

Patients were eligible if they had a histologically confirmed diagnosis of a target rare cancer (UGB, NEN or GYN cancers) that was advanced or metastatic, an Eastern Cooperative Oncology Group (ECOG) performance status of 0–1, a measurable tumor lesion per RECIST 1.1 criteria^61^ and screening blood laboratory values largely within normal limits. Prior systemic therapy or radiotherapy was permitted if completed at least 4 or 2 weeks, respectively, of the first administration of the study drugs and all related adverse events had stabilized or returned to baseline. The exclusion criteria included active central nervous system metastases (brain or leptomeningeal); prior CICB (monotherapy was permitted); prior malignancy active in the previous 3 years; active, known or suspected autoimmune conditions; and requirement for systemic corticosteroids >10 mg prednisolone daily or equivalent. Participants provided fully informed written consent, including for the collection and analysis of biospecimens (including fecal samples) and sharing of anonymized data as part of research collaborations. The data cutoff was May 7, 2022, providing a minimum of 26 months of follow-up for all participants.

All patients were intended to be treated with CICB in the form of nivolumab 3 mg kg^−1^ and ipilimumab 1 mg kg^−1^ three weekly for four doses (induction), followed by nivolumab monotherapy maintenance (3 mg kg^−1^ two weekly or 480 mg four weekly after a protocol amendment) for up to 2 years or until PD or unacceptable toxicity. The trial’s prespecified primary endpoint was to determine the clinical efficacy of CICB in patients with rare cancers using the RECIST 1.1 BOR^61^. In brief, BOR was determined at data cutoff and defined as the investigator-assessed RECIST 1.1 best response designation at any on-trial time point until the date of objectively determined progression per RECIST 1.1 or the date of subsequent anticancer therapy commencement. For participants without documented progression or subsequent therapy, all available response designations contributed to their BOR assessment. The trial’s minimum duration criterion for the determination of SD was 9 weeks.

For the assessment of radiographic response, all patients were intended to undergo whole-body cross-sectional imaging with computed tomography or magnetic resonance imaging at baseline (within 28 days before registration), 12 weeks, 18 weeks and then 12 weekly thereafter (±1 week). Patients with rapid disease-related clinical deterioration who were thus unable to undergo restaging imaging at the first restaging time point were deemed to have cPD. PFS and OS were determined from the date of first treatment; the efficacy and safety outcomes for various trial subcohorts have been reported previously^23–26^. Given the accumulating evidence of ‘pseudoprogression’ in a minority of ICB recipients^62^, under the trial protocol, ICB therapy could extend beyond RECIST 1.1-defined PD if there was investigator-assessed clinical benefit and good participant tolerance of the study drugs until there was evidence of a further 10% or greater increase in target lesion dimensions or further new disease sites.

#### Other clinical metadata

Detailed information on tumor characteristics, demographic factors, blood laboratory values and concomitant medications was collected by the site investigators into an electronic case report form. For this analysis, we included the following 15 clinical metadata variables, as we hypothesized their potential relevance to treatment response and/or gut microbial compositions based on our literature review: patient age (years, at time of trial commencement), sex, body mass index, ECOG performance status, histology cohort (based on the pathology report), extent of measurable tumor (based on the sum of RECIST target lesion diameters calculated using the computed tomography scan at trial screening), study site, season of fecal sample collection, antibiotic use, proton-pump inhibitor use, chemotherapy use, blood NLR, platelet count, albumin levels and lactate dehydrogenase levels (Supplementary Table 3). Only one participant had received prior ICB monotherapy (a NEN cohort patient treated with anti-PD-1 therapy ceased 20 months before trial treatment); given that only one patient was involved, this was not included as a clinical variable. Antibiotic, proton-pump inhibitor and chemotherapy use was defined as their recorded use within the 8 weeks before cycle 1 of study treatment, given the evidence of antibiotic perturbations of gut microbial compositions lasting this duration^63^. The different antibiotics used were amoxicillin, amoxicillin plus clavulanic acid, ampicillin, azithromycin, cefalexin, cefazolin, ceftriaxone, clindamycin, co-trimoxazole, doxycycline, flucloxacillin, gentamicin, norfloxacin, penicillin, piperacillin plus tazobactam and metronidazole. As only 9 of the 106 microbiome-evaluable patients had used any antibiotics in this 8-week period, they were not further subcategorized based on class or antimicrobial coverage. The different proton-pump inhibitors used were esomeprazole, pantoprazole, rabeprazole and omeprazole.

#### Fecal sample collection

The collection of fecal samples was added to the study protocol in version 5 (July 24, 2017). Participants were trained and provided OMR-200 ‘OMNigene GUT kits’ (DNA Genotek) to collect a fecal sample immediately before treatment (from day −7 to day 0 relative to cycle 1 of trial treatment). OMR-200 kits are designed to stabilize DNA and have been shown to enhance DNA quantifications and stability across storage temperatures relative to nonpreservative alternatives^64^. Fecal samples were express-shipped to the Olivia Newton-John Cancer Research Institute, where they were then frozen at −80 °C for long-term storage. DNA was extracted using the FastDNA kit (MP Biomedicals), including a negative control using ultrapure water. DNA samples were shipped to the Wellcome Sanger Institute on dry ice for shotgun metagenomic sequencing.

### Fecal shotgun metagenomic sequencing and analysis

#### DNA sequencing and quality control

DNA samples were quantified using a Qubit fluorometer, and whole metagenome libraries were deeply sequenced on a single run of the NovaSeq 6000 S4 platform (2 × 150-bp paired-end reads), generating a median of 20,477,028 raw paired-end reads per sample (interquartile range 19,244,530–22,056,539 paired-end reads). Raw sequencing data were first human decontaminated by the Wellcome Sanger Institute core sequencing team by removing read pairs in which one or both aligned to the GRCh37 human genome assembly using bwa (v0.7.17; ‘aln’ then ‘sampe’ commands)^65^. These data were further quality controlled using the metaWRAP (v1.2)^66^ ‘reads_qc’ pipeline, which first trimmed low-quality bases using trim-galore (v0.6.7)^67^ (default parameters) and then performed a second pass of human decontamination with BMTagger (v3.101)^68^ using the GRCh38 human genome assembly. Finally, a median of 20,359,318 clean paired-end reads per sample (interquartile range 19,014,843–21,771,873) were available for further analysis.

#### MAG assembly

Quality-controlled paired-end reads were first assembled individually with SPAdes (v3.14) using option ‘-meta’ (refs. ^69,70^). Unassembled reads were then recovered by mapping raw reads back to metaSPAdes-assembled contigs using bwa ‘mem’ (v0.7.17)^65^, followed by reassembly with MEGAHIT (v1.2.4)^71^ using default parameters. Subsequently, the sample-wise metaSPAdes and MEGAHIT assemblies were combined and sorted, with short contigs (<1,500 bp) removed. The resulting assemblies were then independently binned with MetaBAT 2 (v2.13)^72^, MaxBin2 (v2.2.4)^73^ and CONCOCT (v0.4)^74^ using default parameters and a minimum contig length threshold of 1,500 bp (option ‘--minContig 1500’). The depth of contig coverage required for the binning was inferred by mapping the raw reads back to their assemblies with bwa-mem and then calculating the corresponding read depths for each contig with samtools (v1.5)^75^ (‘samtools view -Sbu’ followed by ‘samtools sort’), together with the ‘jgi_summarize_bam_contig_depths’ function in MetaBAT 2.

Thereafter, individual bin sets produced by the three binning programs were consolidated into a refined bin set consisting of the best version of each bin based on the most optimal genome completion and contamination metrics among all seven versions of hybridized bin sets (MetaBAT 2, MaxBin2, CONCOCT, MetaBAT 2 + MaxBin2, MetaBAT 2 + CONCOCT, MaxBin2 + CONCOCT, MetaBAT 2 + MaxBin2 + CONCOCT), as estimated by CheckM (v1.1.2)^76^ using the metaWRAP (v1.2) ‘bin_refinement’ pipeline^66^. Finally, the final bin sets were further improved by performing reassembly with SPAdes in ‘--careful’ mode after both strict and permissive mapping of raw reads and keeping the bin sets with the best CheckM metrics. In total, 4,277 MAGs with ≥50% completion and ≤5% contamination were generated. These were then further quality controlled, now for ≥90% completeness and ≤5% contamination using CheckM2 (v0.1.3)^77^ and for strain-level contamination using GUNC (v1.0.5)^78^ to finally identify 2,209 quality-controlled nc-MAGs consistent with the MIMAG (minimum information about a MAG) criteria^79^. Finally, study-specific MAGs were taxonomically classified (using GTDB r207 taxonomy) with GTDB-tk (v2.1)^80^, pplacer (v1.1)^81^ and fastANI (v1.3)^82^.

#### Generation of a custom, MAG-informed reference database

As the recovery of MAGs may be challenging for some (for example, low abundance or difficult to assemble) strains, we sought to supplement our study-specific strain genome reference database with SRGs from GTDB r207 (62,291 bacterial and 3,412 archaeal genomes) to create a ‘hybrid’ reference library. To identify a relevant shortlist of GTDB SRGs, we first mapped quality-controlled reads from our study to the full GTDB r207 SRG database with Bowtie 2 (v2.3.5)^83^ and inStrain (v1.3.0)^84^ (using default settings in ‘--database’ mode). After further filtering of reads mapped to <0.5 SRG breadth, we determined that *n* = 1,076 SRGs were present. We combined these SRGs with the study-specific nc-MAGs (total 3,285) and used dRep (v2.0.0)^85^ to dereplicate the combined genome set to 98% identity using the settings ‘-comp 90 -con 5 --S_algorithm fastANI --S_ani 0.98 --cov_thresh 0.50 --multiround_primary_clustering --greedy_secondary_clustering’. An absolute nucleotide identity (ANI) threshold of 98% was chosen as a compromise between offering subspecies (strain-level) resolution for read classification while still mitigating ‘read stealing’ due to overly similar reference genomes (as detailed in the inStrain documentation). Ultimately, *n* = 1,397 genomes were selected using dRep and formed our ‘hybrid’ custom strain reference database. Of these, just over half were study-specific nc-MAGs (714, 51%), whereas the remainder were either near-complete isolate (423, 30%) genomes or nc-MAG (260, 19%) SRGs. Using GTDB-tk, we could classify 1,363 of the 1,397 genomes to 904 separate GTDB r207 species clusters (898 bacteria, 6 archaea), with the remaining 34 (32 bacteria, 2 archaea) representing completely new species. For the 904 ‘known’ species, 705 species had 1 strain, whereas 199 species had 2–21 strains each. The species with *n* = 21 distinct strains by 98% ANI delimitation was *Ruminococcus D bicirculans* (Supplementary Table 16).

#### Read mapping to a custom strain database

We first used Bowtie 2 to generate a mapping index and then to align reads to our custom reference database. We then used the inStrain profile, now with settings ‘--min_read_ani 0.95 --min_genome_coverage 1’, to perform more precise quality control of the mapped reads. InStrain uses information on paired-end read orientation, mapQ score, insert size and ANI value to filter read mappings stringently, resulting in high-confidence quantifications.

To enhance our confidence about read mappings further, we removed reads mapped with <0.5 genome breadth coverage, as low genome breadth might indicate mapping to mobile genetic elements or mismapping. For our discovery cohort, a median of 50% (range 39–73%) of quality-controlled reads were ultimately used for abundance estimation of strains within each sample (Supplementary Fig. 1).

We finally used Decontam (v1.16.0)^86^ to screen for potential contaminants. Reassuringly, after the above steps, no bacteria were identified in our negative control sample for the discovery cohort. Based on the ‘frequency’ method (inverse correlation between the abundance of strains and the DNA concentration of submitted samples), one strain was identified as a potential contaminant in over 10% of samples from our discovery cohort (CA209-538 cohort) and was thus removed (*Pseudomonas E* sp002874965; Supplementary Fig. 4).

#### Downstream analysis of taxonomic abundances

Most downstream microbiome analyses were performed in the R (v4.1.0) environment, using ‘phyloseq’ (v1.12.0)^87^, ‘microbiome’ (v1.12.0)^88^ and ‘vegan’ (v2.6.4). Specifically, alpha diversity was computed using the Shannon diversity index on strain relative abundances (each sample’s sum abundances transformed to a sum of 1). As we found no association between the Shannon diversity index and clean paired-end reads in our discovery cohort (Pearson *R* = 0.068, *P* = 0.49), we did not perform rarefaction. Beta diversity was calculated using the strain Aitchison distance, a measure of Euclidean distance of CLR-transformed abundances, computed using log(*a*/gma), where *a* is the species relative abundance and gma is the sample geometric mean relative abundance (with a small pseudocount of one-half the minimum nonzero abundance added to all values to account for zeros). As CLR abundances may better account for the inherent compositionality of microbial abundance data^89^, CLR-transformed feature abundances were exclusively used for the supervised ML analyses.

#### Generation and visualization of phylogenetic trees

For whole bacterial kingdom genome sets, approximately maximum-likelihood phylogenetic trees were constructed using GTDB-tk (v2.1.0)^80^ (aligning 120 ubiquitous bacterial genes) and FastTree (v2.1.0)^90^ using the WAG model (Fig. 3a,b). For the tree of *Faecalibacterium* genomes, pairwise whole-genome ANI distances were computed using FastANI^82^ (many-to-many mode), which was converted into a distance matrix and then to a Newick-format tree using rapidNJ (v2.3.3)^91^ (Supplementary Fig. 2). Trees were visualized using the R package ggtree (v3.2.1)^92^.

#### Functional annotation

To evaluate the presence of virulence factor genes, we used abricate (v1.0.1)^93^ to screen relevant strain genomes against the VFDB (Virulence Factor Database)^94^. To profile strain metabolic potential broadly, we used gapseq (v1.2)^95^ using the ‘gapseq find’ command with default settings. Briefly, this involved performing a homology search of genomes (using TBLASTN (10.1186/1471-2105-10-421)) for 28,768 reactions from 2,910 metabolic pathways (curated from MetaCyC and manually). Metabolic pathways were deemed present if ≥80% complete (lowered to ≥67% if ‘key’ reactions were present).

To evaluate butyrate production potential specifically, we used a previously validated multilevel approach involving hidden Markov models (HMMs)^44,96^. Briefly, we used a published database of 1,716 genomes and 19,284 genes to build HMM profiles (using HMMER v3.2.1; http://hmmer.org/) for the six genes encoding the acetyl-CoA butyrate-producing pathway (responsible for butyrate production through carbohydrate degradation). These genes are acetyl-CoA acetyltransferase (*thl*), β-hydroxybutyryl-CoA dehydrogenase (*bhbd*), crotonase (*cro*), butyryl-CoA dehydrogenase (*bcd*), and the alternative terminal enzymes butyryl-CoA:acetate CoA transferase (*but*) and butyrate kinase (*butk*). We then used these models to screen the strain genomes for the presence of these respective genes. As an orthogonal approach, we also mapped cleaned sample paired-end reads to the above genes’ sequences using Bowtie 2 and then used inStrain ‘quick profile’ to count mappings to estimate their sample-wise gene abundance (normalized per million reads) agnostic of source strain. The output is available in Supplementary Tables 13 (strain_top22_acetylcoa_pwy) and 14 (sample_acetylcoa_pwy).

### Supervised ML analysis

Supervised ML analyses were performed in the Python 3 environment using the packages sklearn (v1.1.1)^97^, imblearn (v0.9.1)^98^ and their dependencies. The supervised ML pipeline involved a preprocessing step before model training and testing, performed separately for each training and testing instance to ensure no data leakage. This involved standard-scaling numerical features (computed using the formula *z* = (*x* *−* *u*)/*s*, where *x* is the feature value (for example, the CLR-transformed strain abundances), *u* is the mean of the fold samples and *s* is the s.d. of the fold samples) and one-hot encoding categorical features. Subsequently, only before classifier training (but not testing), classes of the target variable (RvsP or PFS12) were balanced with random oversampling with replacement.

We chose to use RF as our classifier, given its, on average, superior performance using microbial feature sets in previous benchmarking studies^31^. RF uses bootstrapped data to create an ensemble of decision trees (each trained on a subset of features), with the ultimate classification based on consensus; thus, it is feature scale invariant and able to capture nonlinearities and is also interpretable using TreeSHAP (described subsequently).

Our hyperparameter tuning procedure involved a random hyperparameter search over a broad array of options with 1,000 separate combinations tested, aiming to maximize the ROC AUC averaged over 20 times repeated fivefold cross-validation (that is, 100 separate models trained and tested (splits), for each 1,000 iterations, for each feature and classifier combination).

ROC AUC is a popular classifier performance metric that evaluates the discriminative performance across all potential decision thresholds, thus allowing for a head-to-head comparison of differently calibrated classifiers^99^. Ultimately, the best hyperparameter combination (based on mean AUC) was selected and referred to as the ‘tuned’ pipeline. The optimal hyperparameters and AUC scores for all 100 splits for all full feature sets are listed in Supplementary Table 9 (hyperparam_tuning_all).

To evaluate model performance, we used cross-validation (for example, leave-one-histotype-out cross-validation) or completely separate training and test cohorts (for example, training a model using one study cohort and then testing the fitted model on another cohort). Whenever evaluating model performance, training and testing procedures were repeated 100 times, and the resultant predictions were averaged to account for the stochasticity of our RF pipeline. As with hyperparameter tuning, ROC AUC was our metric of choice for gauging model performance.

Feature importances were evaluated with the ‘shap’ package using the TreeExplainer() function. Based on the foundation of game theory, TreeExplainer computes the influence of each feature (strain abundance) in determining the RF classifier’s local (per-sample) prediction. Therefore, we computed global feature importances (cohort-wide average of the absolute TreeExplainer scores) and imputed the importance ‘direction’ (that is, positive or negative influence on response prediction) by constructing a simple linear model between the feature values and SHAP values. We repeated this procedure 1,000 times to account robustly for the RF pipeline’s stochasticity. The global feature importance and s.d. values of all features are listed in Supplementary Table 12 (strain_importance).

### Literature review and meta-analysis of relevant published datasets

We sought to identify all published clinical datasets that met the following criteria:

1. Evaluated baseline fecal microbiota from patients with cancer who were about to commence only ICB (anti-PD-1, anti-CTLA-4 or CICB) therapy. ‘Baseline’ samples were defined as those collected between day −15 and day 15 relative to the start of ICB to ensure that the profile reflected the patient’s gut microbial context immediately before treatment and that the gut microbial profile had not been already affected by ICB therapy (for example, anti-CTLA-4 appears to modify gut barrier integrity^51^ and thus could feasibly change microbial compositions).
2. Used short-read, paired-end shotgun metagenomic sequencing (to allow us to standardize and maintain stringency in our bioinformatic pipeline and quality control steps).
3. Reported tumor response. To be pragmatic, we accepted radiographic (using RECIST 1.1) or pathological response. However, we excluded studies that reported only PFS12 or where response was binned with SD.

To find all such datasets, we performed a structured PubMed database search combining the following three search strings that used both MeSH (Medical Subject Headings) terms and title and/or abstract keywords:

‘neoplasms’[MeSH Major Topic] OR ‘cancer’[Title/Abstract] OR ‘malignancy’[Title/Abstract] OR ‘tumor’[Title/Abstract]

OR

‘immune checkpoint inhibitors’[MeSH Terms] OR ‘pembrolizumab’[Title/Abstract] OR ‘nivolumab’[Title/Abstract] OR ‘atezolizumab’[Title/Abstract] OR ‘avelumab’[Title/Abstract] OR ‘durvalumab’[Title/Abstract] OR ‘cemiplimab’[Title/Abstract] OR ‘dostarlimab’[Title/Abstract] OR ‘ipilimumab’[Title/Abstract] OR ‘tremilimumab’[Title/Abstract] OR ‘immunotherapy’[Title/Abstract] OR ‘immune checkpoint’[Title/Abstract]

OR

‘microbiota’[MeSH Terms] OR ‘metagenome’[MeSH Terms] OR ‘metagenomics’[MeSH Terms] OR ‘microbiome’[Title/Abstract] OR ‘microbiota’[Title/Abstract]

In total, this search yielded 1,181 records up to December 31, 2022. Titles and abstracts were manually reviewed to identify a total of 28 unique studies meeting eligibility criterion 1. A manual bibliography search yielded a further three studies meeting eligibility criterion 1 (Supplementary Table 17 (lit_review)). Of these, 19 studies used shotgun metagenomics, and 13 studies made these raw data available. Three studies were excluded as the shotgun metagenomic data were single end (Ion Torrent). Finally, of the remaining ten studies, four were excluded as they did not report response, yielding six studies that could be included in our meta-analysis (see Supplementary Fig. 3 for a PRISMA-style flowchart). Metadata for each cohort were curated from the corresponding publication tables or relevant sequencing repositories (for example, Sequence Read Archive, European Nucleotide Archive).

Shotgun metagenomic sequencing data for the six evaluable cohorts were downloaded and analyzed using a uniform bioinformatic procedure (as described earlier), including FASTQ file quality control and human DNA decontamination, and then read mapping to an identical custom strain database (generated from CA209-538 MAGs) using identical settings of Bowtie 2 and inStrain. Despite a wide range in the number of quality-controlled paired-end reads per sample, in general, all were deeply sequenced (Table 2). Subsequent downstream analysis of gut microbial profiles and supervised ML analyses were performed using identical methods to those previously described.

### Statistical analysis

Statistical tests are cited in the text. In general, nonparametric statistical tests were preferred (all were two-sided). To determine associations between an ordinal and a numeric variable (for example, BOR versus a numeric metadata variable), we used the Kendall *τ* test. For associations between a binary and a numeric variable, the Mann–Whitney *U* (also known as the Wilcoxon rank-sum) test was used. For associations between a nonordinal categorical variable and a numeric variable, the Kruskal–Wallis test was used. The threshold for significance was set as a two-tailed *P* value of <0.05. Data were processed and visualized using the R packages ‘tidyverse’ (v2.0.0)^100^, ‘ggpubr’ (v0.6.0), ‘survival’ (v3.5.5)^101^, ‘survminer’ (v0.4.9) and ‘table1’ (v1.4.3) and the Python packages ‘numpy’ (v1.23.3)^102^, ‘pandas’ (v1.4.3) and ‘matplotlib’ (v3.5.1)^103^. For all boxplots, the center line indicates the median, box limits indicate the upper and lower quartiles, and whiskers indicate 1.5× the interquartile range.

### Reporting summary

Further information on research design is available in the Nature Portfolio Reporting Summary linked to this article.

## Online content

Any methods, additional references, Nature Portfolio reporting summaries, source data, extended data, supplementary information, acknowledgements, peer review information; details of author contributions and competing interests; and statements of data and code availability are available at 10.1038/s41591-024-02823-z.

### Supplementary information

Supplementary Information Supplementary Figs. 1–4 and CA209-538 clinical trial protocol version 8.

Reporting Summary

Supplementary Tables Supplementary Tables 1–19.

## Data Availability

All CA209-538 fecal shotgun metagenomic sequencing data (after first-pass human decontamination) have been deposited to the European Nucleotide Archive (study accession no. ERP134027). The 1,397 quality-controlled (near-complete) study-specific genomes used as the custom reference database have been deposited to Zenodo (10.5281/zenodo.10450122). CA209-538 clinical metadata and strain abundance data necessary to replicate our analyses are provided in the Supplementary Tables. The six publicly available shotgun metagenomics datasets were downloaded using the following accession numbers: EGAS00001006982 (2022_Simpson), PRJEB43119 (2022_Lee), PRJNA762360 (2022_McCulloch), EGAD00001006734 (2021_Andrews), PRJNA399742 (2018_Matson) and PRJNA397906 (2017_Frankel). Permission to access the 2021_Andrews raw sequencing dataset was kindly provided by J. Wargo and The University of Texas M.D. Anderson Cancer Center. Permission to access the 2022_Simpson raw sequencing data was kindly provided by G. Long and the Melanoma Institute of Australia. Associated sample-level clinical metadata for external datasets were collected from their relevant publications, the relevant sequencing repository or an associated GitHub repository.

## Code availability

No unique software or computational code was created for this study. The relevant code to replicate our supervised machine learning analyses of CA209-538 data, using the data in Supplementary Table 8 (metadata_and_clr_abundances), is available at https://github.com/agunjur/cancer_microbiome_CICB/.
