# Supplementary material for: A gut microbial signature for combination immune checkpoint blockade across cancer types
Source: Nat Med. 2024 Mar 1;30(3):797–809. doi: 10.1038/s41591-024-02823-z (PMC10957475; doi:10.1038/s41591-024-02823-z)
Supplement: Supplementary file 1 — Supplementary Figs. 1–4 and CA209-538 clinical trial protocol version 8. [file 41591_2024_2823_MOESM1_ESM.pdf]

nature medicine

Article https://doi.org/10.1038/s41591-024-02823-z

A gut microbial signature for combination immune checkpoint blockade across cancer types

In the format provided by the authors and unedited

Supplementary Figure 1

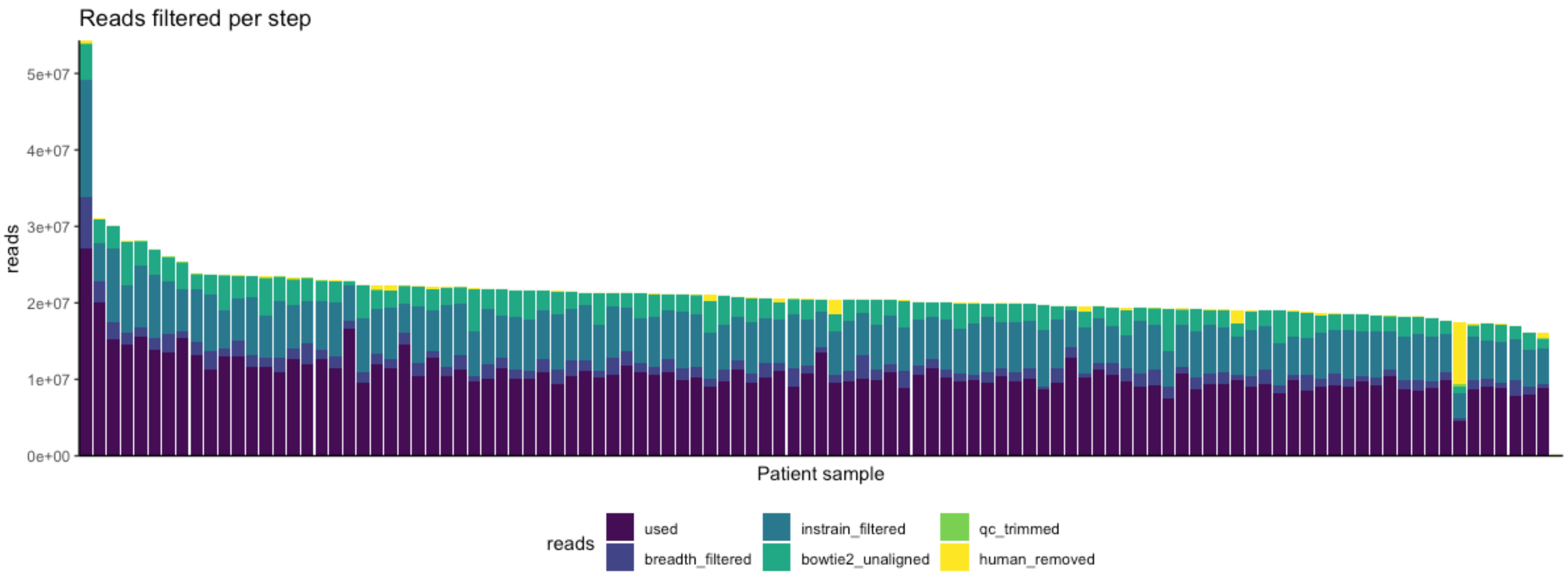

Supplementary Figure 1 caption: Bar chart depicting proportion of paired-end reads for each CA209-538 cohort sample filtered by each quality-control step, and ultimately ‘used’ (for taxonomic abundance estimation).

Step 1 = ‘human_removed’ = reads removed by first-pass human decontamination.

Step 2 = ‘qc_trimmed’ = reads removed by metaWRAP ‘reads_qc’ quality control.

Step 3 = ‘bowtie2_unaligned’ = reads unaligned by bowtie2, mapping to the custom strain genome library.

Step 4 = ‘inStrain_filtered’ = reads filtered by inStrain.

Step 5 = ‘breadth_filtered’ = reads removed due to aligning to a microbial genome with < 0.5 genome coverage.

Supplementary Figure 2

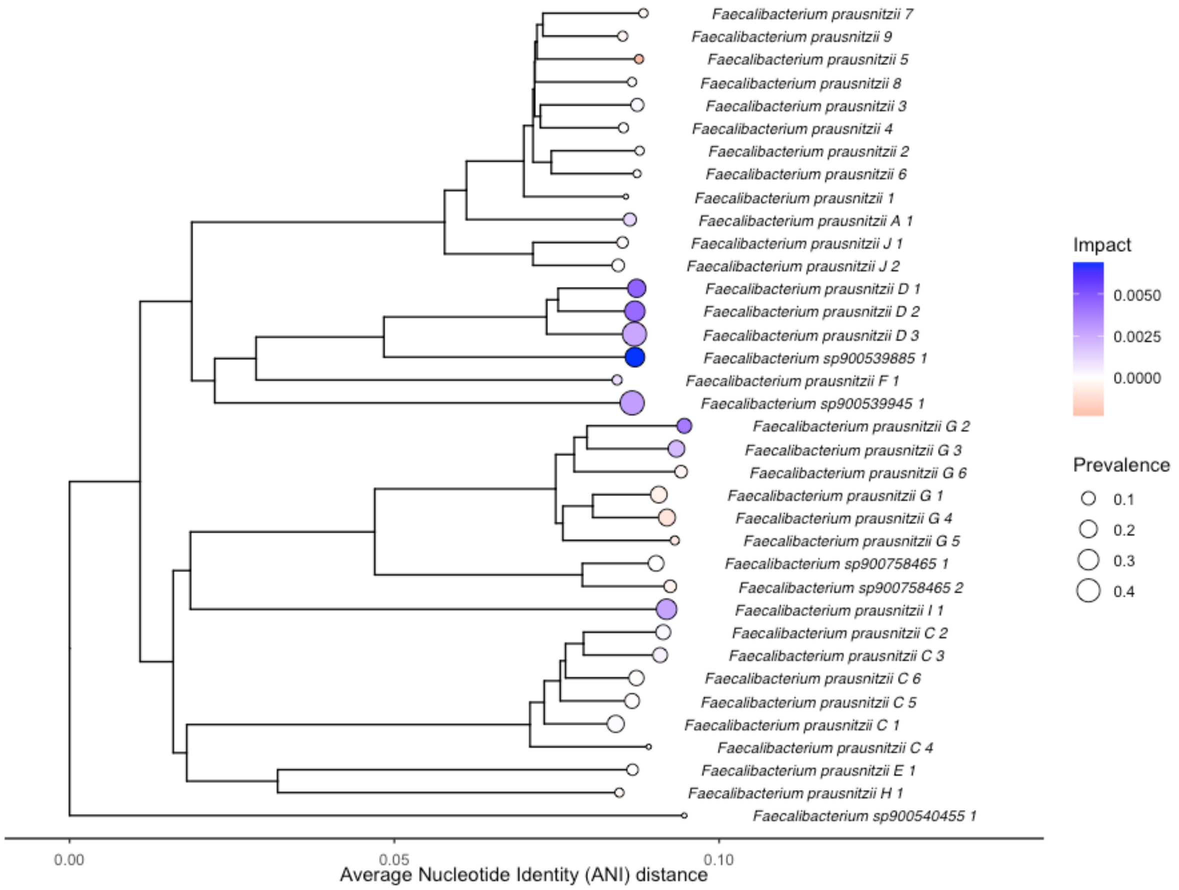

Supplementary Figure 2 caption: Phylogenetic tree depicting all Faecalibacterium (n=35) strain genomes, midpoint rooted. Tree constructed using whole-genome average nucleotide identity (ANI) distances and FastTree. Tips coloured by Strain Impact (i.e. impact on RvsP predictions), and tip size by strain prevalence in the CA209-538 cohort. This highlights a particularly 'positive' clade of *Faecalibacterium* (representing the closely related GTDB species *Faecalibacterium prausnitzii D* and *Faecalibacterium sp900539885*).

Supplementary Figure 3

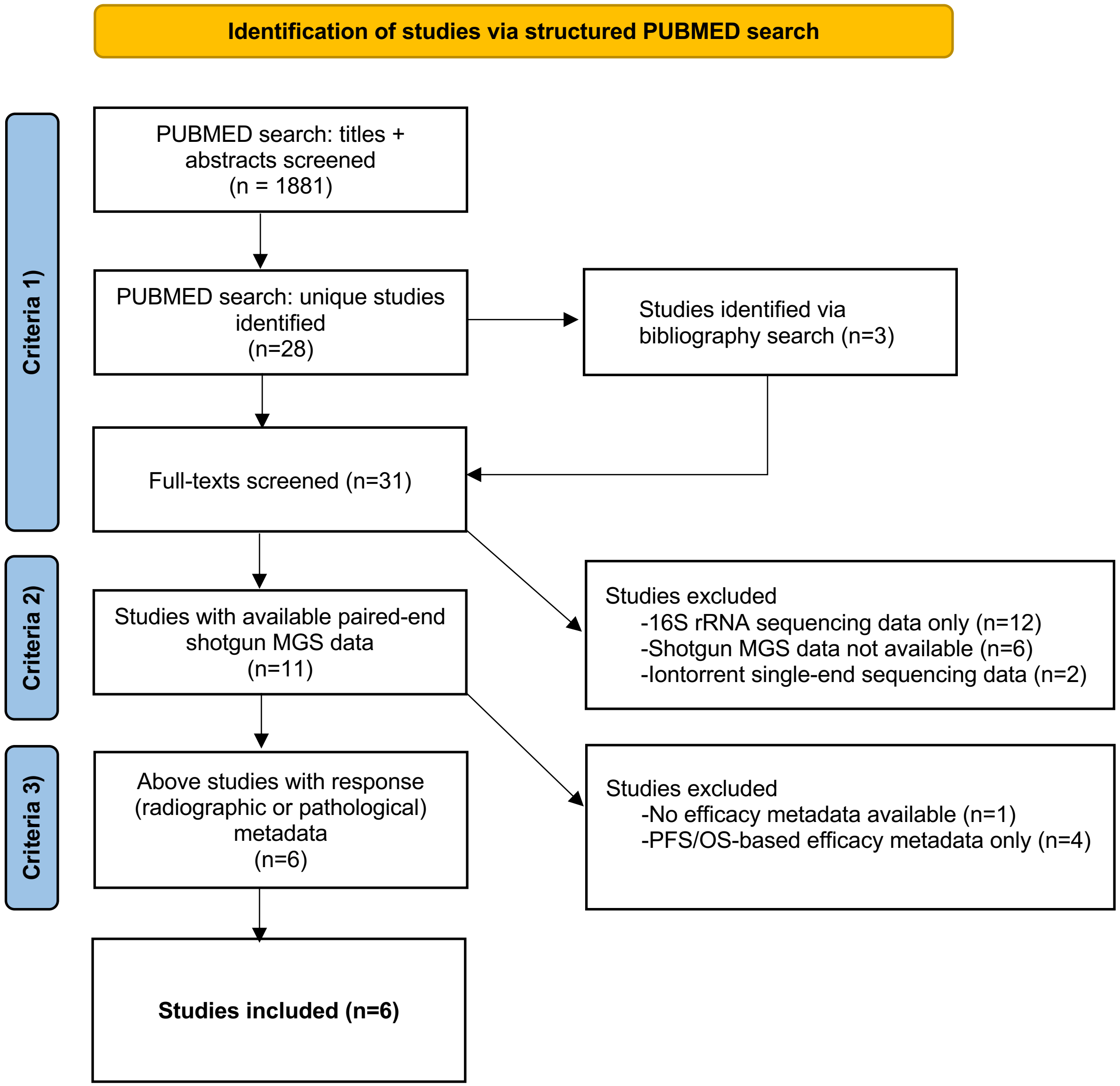

Supplementary Figure 3 caption: PRISMA flow diagram for structured-search literature review. Full details of all the Full-texts screened are available in Supplementary table 17 (lit_review).

Supplementary Figure 4

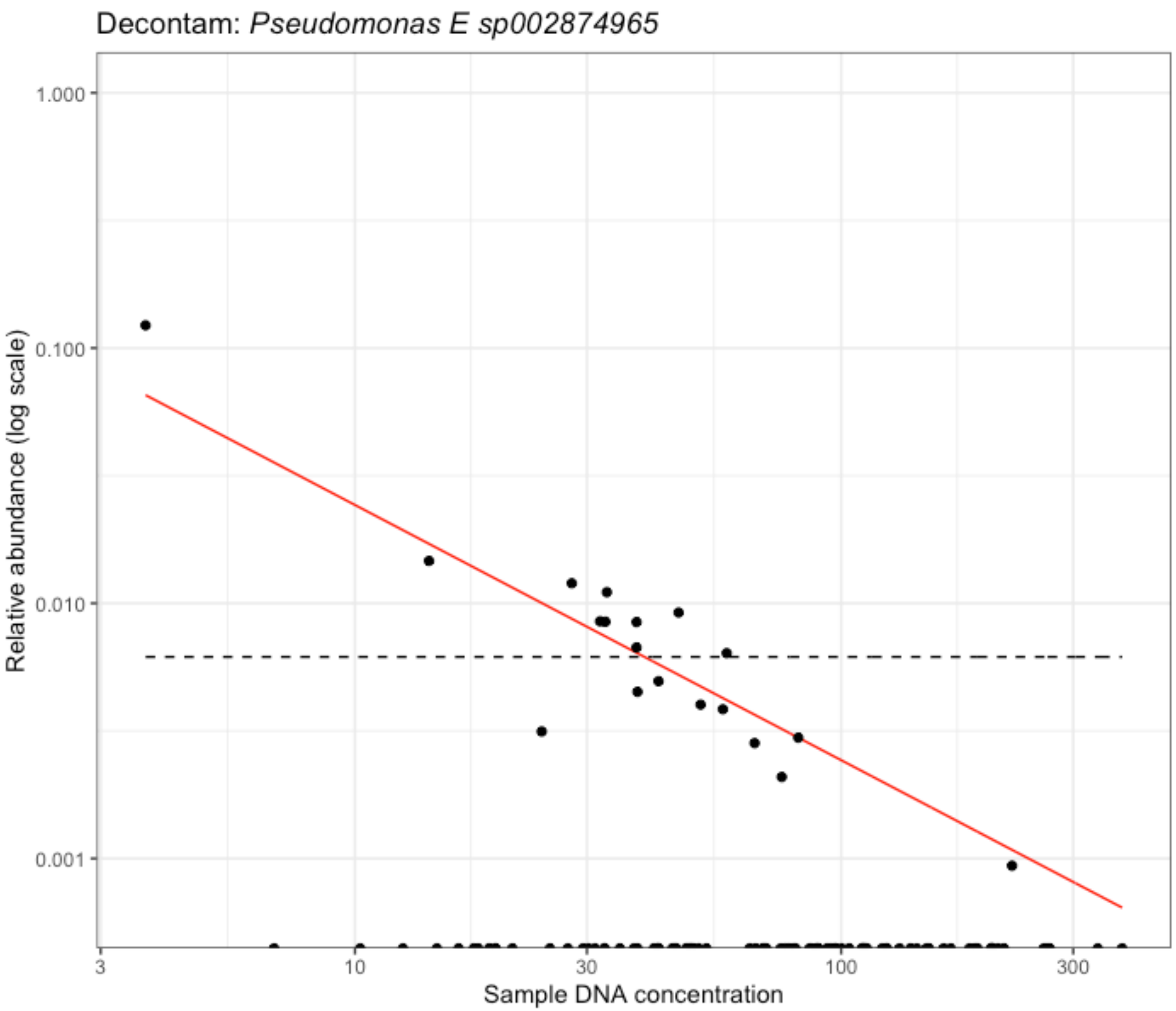

Supplementary Figure 5 caption: Plot of relative abundance vs submitted sample DNA concentration of taxon "Pseudomonas E sp002874965", present in n=19/106 CA209-538 samples. The inverse linear relationship between taxon log relative abundance and submitted sample DNA concentration, and environmental nature of *Pseudomonas* taxa, made this taxon a likely contaminant. Thus, it was removed from downstream analysis.

CA209-538 / ONJ2016-001 clinical trial protocol version 8

A Phase II Clinical Trial Evaluating Ipilimumab & Nivolumab in Combination for the Treatment of Rare Gastrointestinal, Neuro-Endocrine and Gynaecological Cancers

Date of Protocol: 18 March 2020

Protocol Version: 8

Protocol Number: CA209-538

Local Sponsor Protocol Number: ONJ2016-001

Investigator Name & Contact Information:

Coordinating Investigators:

Prof Jonathan Cebon
Olivia Newton-John Cancer Research Institute, Level 5 ONJCWC, Austin Health, 145 Studley Rd, Heidelberg, 3084, Victoria, Australia

Dr Oliver Klein
Olivia Newton-John Cancer Research Institute, Level 5 ONJCWC, Austin Health, 145 Studley Rd, Heidelberg, 3084, Victoria, Australia

Other Site Principal Investigators:

- Assoc Prof Clare Scott
  Peter Macallum Cancer Centre (PMCC)
- Dr Damien Kee
  PMCC & Austin Health
- Dr Andrew Strickland
  Monash Health
- Dr Craig Underhill
  Border Medical Oncology
- Associate Professor Matteo Carlino
  Blacktown Hospital

Local Sponsor: Olivia Newton-John Cancer Research Institute
Email: trials@onjcri.org.au

Trial Sites:

- Austin Health
- Peter Macallum Cancer Centre
- Monash Health
- Border Medical Oncology, Albury Wodonga Regional Cancer Centre
- Blacktown Hospital

SUMMARY OF CHANGES TO PROTOCOL CA209-538/ONJ2016-001

Version	Date	Summary of changes	Reason for change
3		Original	
4	16/05/2017	Addition of sites (Monash Health, Peter Mac); administrative amendments to language.	Trial site expansion to provide access to more participants; correction to wording to allow for consistency of endpoints.
5	24/07/2017	Addition of collection of stool samples,	To determine gut bacterial composition.
6	23/08/2018	Section 1. Addition of regional study site plus additional metropolitan site.<br><br>Section 1; table 3.1-1 Addition of 60 participants – total of 120<br><br>Section 2.5: Addition of Medical Monitor contact details<br><br>Decrease in study schema assessments post Cycle 5.<br><br>All sections – error corrections	Allow better access to the trial for regional patients (decreased QOL impact)<br>Unprecedented interest in trial, and funding provided by Dept Health.<br><br>Lead appointee (or delegate), role defined<br><br>Participants reaching cycle 5 and beyond will have fewer mandatory assessments to decrease the impact of participation, any deemed clinically relevant will still be recorded in their medical record under the direction of their treating clinician.<br><br>Correction of general typographical errors
7	7th March 2019	Error in text<br>3.1 Schedule of imaging inconsistent with schedule of assessments<br>5.2 Schedule of weight & ECOG inconsistent with schedule of assessments	
8	18 Mar 2020	Revision to Site and PI details on cover page<br><br>Permission of Q4W Nivolumab dosing in exceptional circumstances<br><br>Removal of need to re-consent participants in cases of treatment beyond progression<br><br>Clarification that progression of the cancer under study will not be classified as an SAE	Correction of errors<br><br>Due to COVID-19 pandemic<br><br>Correction of error<br><br>In line with other ONJCRI protocols

Contents

SUMMARY OF CHANGES TO PROTOCOL CA209-538/ONJ2016-001 III

1. TRIAL SUMMARY 1

2. INTRODUCTION AND STUDY RATIONALE 1

1.1 Research Hypothesis 2

1.1.1 Primary Objectives 2

1.1.2 Secondary Objectives 2

1.1.3 Exploratory Objectives 2

1.2 Product Development Background 2

1.2.1 Nivolumab Mechanism of Action 2

1.2.2 Ipilimumab Mechanism of Action 4

1.2.3 Rare Cancers: Background 4

1.2.4 Summary of Results from the Ipilimumab and Nivolumab programs 4

1.2.5 Rationale for the Study Design 10

1.3 Overall Risk/Benefit Assessment 12

2 ETHICAL CONSIDERATIONS 13

2.1 Good Clinical Practice 13

2.2 Human Research Ethics Committee 13

2.3 Informed Consent 14

2.4 Confidentiality and privacy 14

2.5 Ongoing Safety Monitoring 15

3 INVESTIGATIONAL PLAN 15

3.1 Study Design and Duration 15

3.2 Post Study Access to Therapy 19

3.3 Study Population 19

3.3.1 Inclusion Criteria 19

3.3.2 Exclusion Criteria 21

3.3.3 Women of Childbearing Potential 22

3.4 Concomitant Treatments 22

3.4.1 Prohibited and/or Restricted Treatments 22

3.4.2 Other Restrictions and Precautions 23

3.4.3 Surgical Resection Following initial Response 23

3.4.4 Permitted Therapy 23

3.5	Discontinuation of Participants from Treatment	23
3.6	Post Treatment Study Follow up	24
3.6.1	*Withdrawal of Consent*	*24*
3.6.2	*Lost to Follow-Up*	*24*
4	TREATMENTS	25
4.1	Study Treatments	26
4.1.1	*Investigational Product*	*27*
4.1.2	*Non-investigational Product*	*27*
4.1.3	*Handling and Dispensing*	*27*
4.2	Method of Assigning Participant Identification	28
4.3	Selection and Timing of Dose for Each Participant	29
4.3.1	*Antiemetic Premedications*	*31*
4.3.2	*Dose Delay Criteria*	*31*
4.3.3	*Dose Modifications*	*33*
4.3.4	*Criteria to Resume Treatment*	*33*
4.3.5	*Discontinuation Criteria.*	*33*
4.3.6	*Treatment of Nivolumab or Ipilimumab Related Infusion Reactions*	*34*
4.3.7	*Treatment Beyond Disease Progression*	*35*
4.4	Treatment Compliance	36
4.5	Destruction and Return of Study Drug	36
4.5.1	*Destruction of Study Drug*	*36*
4.5.2	*Return of Study Drug*	*37*
5	STUDY ASSESSMENTS AND PROCEDURES	38
5.1	Flow Chart/Time and Events Schedule	38
5.2	Study Materials	51
5.3	Safety Assessments	51
5.3.1	*Imaging Assessment for the Study*	*52*
5.4	Efficacy Assessments	52
5.4.1	*Primary Efficacy Assessment*	*53*
5.4.2	*Secondary Efficacy Assessment*	*53*
5.4.3	*Assessment of Overall Tumor Burden and Measurable Disease*	*53*
5.4.4	*Specifications by Method of Measurement*	*54*
5.4.5	*Baseline Documentation of "Target" and "Non-target Lesions"*	*55*
5.4.6	*Tumor Response Evaluation*	*56*
5.4.7	*New Lesions*	*57*

5.4.8 Response Criteria (RECIST 1.1)......57
5.4.9 Best Overall Response......58
5.4.10 Duration of Objective Response......58
5.5 Biomarker Assessments......58
5.5.1 Tumor Tissue Specimens......60
5.5.2 Exploratory Serum Biomarkers......60
5.5.3 Peripheral Blood Mononuclear Cells (PBMCs)......61
5.5.4 Peripheral Blood RNA......61
5.5.5 Whole Blood for SNP Assessment......61
5.5.6 Collection of fecal samples......61
5.6 Results of Central Assessments......62
6 ADVERSE EVENTs......62
6.1 Serious Adverse Events......62
6.1.1 Serious Adverse Event Collection and Reporting......63
6.2 Non-serious Adverse Events......64
6.2.1 Non-serious Adverse Event Collection and Reporting......64
6.3 Laboratory Test Result Abnormalities......64
6.4 Pregnancy......65
6.5 Overdose......65
6.6 Potential Drug Induced Liver Injury (DILI)......65
6.7 Other Safety Considerations......66
7 STATISTICAL CONSIDERATIONS......66
7.1 Sample Size Determination......66
7.1.1 Initial cohort......66
7.1.2 Expansion cohort......66
7.2 Populations for Analyses......66
7.3 Endpoints......67
7.3.1 Primary Endpoint......67
7.3.2 Secondary Endpoint Exploratory Endpoint(s)......67
7.4 Analyses......68
7.4.1 Demographics and Baseline Characteristics......68
7.4.2 Safety Analyses......68
7.4.3 Biomarker Analyses......68
7.5 Interim Analyses......68
8 STUDY MANAGEMENT......68

8.1 Compliance 68

8.1.1 Compliance with the Protocol and Protocol Revisions 68

8.1.2 Monitoring 69

8.1.3 Investigational Site Training 69

8.2 Records 69

8.2.1 Records Retention 69

8.2.2 Study Drug Records 69

8.2.3 Case Report Forms 70

8.3 Clinical Study Report and Publications 70

9 GLOSSARY OF TERMS 71

10 LIST OF ABBREVIATIONS 72

11 REFERENCES 79

1. TRIAL SUMMARY

Trial Phase	*Phase II*
Clinical Indication	Cohort A: Rare Gastrointestinal, Cohort B: Rare –Endocrine, Cohort C: Rare Gynaecological Cancers
Trial Blinding	Open-label study
Treatment	Ipilimumab and Nivolumab
Number of trial participants	60 (20 patients per cohort) plus 60 patients in an expansion cohort.
Trial sites	Austin Health, Peter MacCallum Cancer Centre, Monash Health, Border Medical Oncology, Blacktown Hospital
Estimated enrollment period	*24 months*
Estimated duration of treatment	*24 months*
Duration of Follow up	28 months, and overall survival follow up for up to 5 years

2. INTRODUCTION AND STUDY RATIONALE

This is a phase 2 clinical trial of nivolumab combined with ipilimumab in participants with rare cancers. This study will allow an evaluation of the clinical benefit rate (CR+PR+SD > 3 months), provided by nivolumab combined with ipilimumab. If the safety profile is acceptable and clinically efficacy is seen, this study would support the use nivolumab combined with ipilimumab in participants with these cancers.

Study Rationale

Clinically advanced rare cancers pose a significant clinical challenge because evidence based treatments are seldom available for patients suffering from these malignancies. Despite little evidence that shows clinical benefit, these patients are often treated with chemotherapeutic agents that are used in patients with more common malignancies that arise from the same anatomical site. Furthermore, because of small numbers, they are often excluded from clinical trials with newer agents. The Rarecare project has defined a rare malignancy as a cancer with an incidence of less than 6/100000/year (1). It is estimated that 42,000 people are diagnosed with a form of rare or less common cancer in Australia every year (www.canceraustralia.gov.au). The cancer specific survival of patients diagnosed with a rare malignancy is significantly lower than with common cancers highlighting the need to improve management and treatment of these patients (2). Given the recent success of cancer immunotherapy with checkpoint regulators such as ipilimumab and nivolumab in a whole range of different cancer types (3), it can be postulated that these agents could be beneficial in rare cancers and improve the overall outlook of patients with these conditions(4). It is proposed here that patient cohorts which fall within three distinct tumour streams will be examined, with all patients receiving ipilimumab and nivolumab as combination immunotherapy. The three tumour streams are defined as: upper GI malignancies (comprising intra-hepatic/ extra-hepatic cholangiocarcinomas, gall bladder cancers and duodenal cancers).); neuroendocrine tumours (Inc. Pancreatic, bronchial and intestinal carcinoid tumours) and rare gynaecological tumours (including but will not be limited to: vaginal or vulval carcinomas, clear cell carcinoma of the ovary, low grade serous ovarian cancer, mixed mullarian tumours (carcinosarcoma), sarcomas of the female genital tract and granulosa cell tumours). Although overall response rates for

individual tumour types will not be established due to sample size (target 20 patients per tumour stream, n=60 total), descriptive information of individual patient responses will guide immune-directed therapies to responsive rare tumour types and may be broadened to tumour streams. Expansion to allow for an additional n=60 patients across all cohorts is likely to significantly improve the power of analysis of overall response rates. Some subtypes of cancers within these groups may demonstrate poor response throughout the trial. The clinical team will discuss ongoing enrolment of these subtypes during monthly clinical meetings to ensure the best outcome for the participants.

1.1 Research Hypothesis

Clinical efficacy of the ipilimumab/nivolumab treatment will be observed in patients with rare cancers.

A common predictive biomarker or immune signature can be identified in responding patients, irrespective of tumour type.

1.1.1 Primary Objectives

To determine the clinical efficacy of the combination treatment of ipilimumab with nivolumab in rare cancers

Primary endpoint: clinical benefit rate for whole population (CR+PR+SD>3months)

1.1.2 Secondary Objectives

Secondary objective of this study is:

To identify whether a common predictive biomarker or immune signature can be identified in responding patients that can occur irrespective of tumor type.

1.1.3 Exploratory Objectives

Exploratory endpoints:

1) Characterisation of immune responses to nivolumab/ipilimumab and predictive biomarker analysis unrestricted by tumor histological type.
2) Characterisation of inflammatory changes in tumours including the trafficking of lymphocytes using functional imaging.

Descriptive endpoint:

Progression free survival and overall survival for each rare tumour type

1.2 Product Development Background

1.2.1 Nivolumab Mechanism of Action

Cancer immunotherapy rests on the premise that tumours can be recognized as foreign rather than as self and can be effectively attacked by an activated immune system. An effective immune response in this setting is thought to rely on immune surveillance of tumour antigens expressed on cancer cells that ultimately results in an adaptive immune response and cancer cell death. This functions by aborting the emergence of tumours as they arise and/or causing tumour shrinkage where it is present. Meanwhile, tumour progression may depend upon acquisition of traits that allow cancer cells to evade immune surveillance and an effective immune response (5). This evasion may occur by exploiting any of the checkpoints that control the regulatory immune response, including display of antigens and control of co-stimulatory pathways that affect the proliferation of cells involved in immunity. Current immunotherapy

efforts attempt to break the apparent tolerance of the immune system to tumour cells and antigens by either introducing cancer antigens by therapeutic vaccination or by modulating regulatory checkpoints of the immune system - either directly by stimulation of immune cells by antibodies directed to receptors on T and B cells or indirectly by cytokine manipulation. T-cell stimulation is a complex process involving the integration of numerous positive, as well as negative, costimulatory signals in addition to antigen recognition by the T-cell receptor (TCR).(6) Collectively, these signals govern the balance between T-cell activation and tolerance to antigens.

Programmed death receptor-1 (PD-1, CD279), a 55 kD type I transmembrane protein, is a member of the CD28 family of T-cell costimulary receptors that also includes CD28, CTLA-4, ICOS, and BTLA(7). PD-1 contains an intracellular membrane proximal immunoreceptor tyrosine inhibitory motif (ITIM) and a membrane distal immunoreceptor tyrosine-based switch motif (ITSM). Two ligands specific for PD-1 have been identified: PD-L1 (B7-H1/CD274) and PD-L2 (B7-DC/CD273). PD-L1 and PD-L2 have been shown to down-regulate T-cell activation upon binding to PD-1 in both murine and human systems(8, 9). PD-1 delivers a negative signal by the recruitment of a protein tyrosine phosphatase SHP-2 to the phosphorylated tyrosine residue in the ITSM in its cytoplasmic region (10, 11). PD-1 is primarily expressed on activated T cells, B cells and myeloid cells(12).

Further evidence for a negative regulatory role of PD-1 comes from studies of PD-1-deficient mice. PD-1-deficient mice develop various autoimmune phenotypes, including dilated cardiomyopathy, a lupus-like syndrome with arthritis and nephritis, and accelerated diabetes mellitus (13-15). The emergence of these autoimmune phenotypes is dependent upon the genetic background of the mouse strain and many of these phenotypes emerge at different times and show variable penetrance. In addition to the phenotypes of null mutations, PD-1 inhibition by antibody-mediated blockade in several murine models has been found to play a role in the development of autoimmune diseases such as encephalomyelitis, graft-versus-host disease, and type I diabetes(16, 17). Taken together, these results suggest that PD-1 blockade has the potential to activate anti-self T-cell responses, but these responses are variable and dependent upon various host genetic factors. Thus, PD-1 deficiency or inhibition is not accompanied by a universal loss of tolerance to self antigens.

Preclinical animal models of tumours have shown that blockade by PD-1 by monoclonal antibodies (mAbs) can enhance the anti-tumour immune response and result in tumour rejection. Antitumor activity by PD-1 blockade functions in PD-L1+ tumours as well as in tumours that are negative for the expression of PD-L1(18-23). This suggests that host mechanisms (i.e., expression of PD-L1 in antigen-presenting cells) limit the antitumor response. Consequently, both PD-L1 positive and negative tumours may be targeted using this approach. In humans, constitutive PD-L1 expression is normally limited to macrophage-lineage cells, although expression of PD-L1 can be induced on other hematologic cells as well, including activated T cells. However, aberrant expression of PD-L1 by tumour cells has been reported in a number of human malignancies(24-29). PD-L1 expressed by tumour cells has been shown to enhance apoptosis of activated tumour-specific T cells in vitro(12). Moreover, the expression of PD-L1 may protect the tumour cells from the induction of apoptosis by effector T cells(30). Retrospective analyses of several human tumour types suggest that tumour over-expression (as measured by IHC) of PD-L1 may permit immune evasion by tumours. In

renal cell carcinoma, high surface expression levels of PD-L1 on tumour cells are related to tumour aggressiveness(31). Participants with high tumour and/or lymphocyte PD-L1 levels are 4.5 times more likely to die from their cancer than participants exhibiting low levels of PD-L1 expression. In addition, in multivariate analysis, high expression of PD-L1 is correlated to have a worse overall survival rate compared to low expression levels of PD-L1(32).

Nivolumab is a fully human, IgG4 (kappa) isotype, mAb that binds PD-1. Blockade of the PD-1 pathway by nivolumab was studied using the mixed lymphocyte reaction (MLR). PD-1 blockade resulted in a reproducible enhancement of both proliferation and IFN-γ release in the MLR.(6) The effect of nivolumab on antigen-specific recall response was investigated using a CMV-restimulation assay with human peripheral blood mononuclear cells (PBMCs), and was evaluated by ELISA.

These data indicated that nivolumab, versus an isotype-matched control antibody, augmented IFN-γ secretion from CMV-specific memory T-cells in a dose-dependent manner. PD-1 blockade by nivolumab is therefore considered a promising immunotherapeutic option.

1.2.2 Ipilimumab Mechanism of Action

CTLA-4 is a key regulator of T cell activity. Ipilimumab is a CTLA-4 immune checkpoint inhibitor that blocks T-cell inhibitory signals induced by the CTLA-4 pathway, increasing the number of tumor reactive T effector cells which mobilize to mount a direct T-cell immune attack against tumor cells. CTLA-4 blockade can also reduce T regulatory cell function, which may lead to an increase in anti-tumor immune response.

1.2.3 Rare Cancers: Background

The three tumour streams that will be studies in this protocol are: (i) upper GI malignancies (comprising intra-hepatic/extra-hepatic cholangiocarcinomas,gall bladder cancers and duodenal cancers).); (ii) neuroendocrine tumours (inc. Pancreatic, bronchial and intestinal carcinoid tumours) and (iii) rare gynaecological tumours (including but will not be limited to: vaginal or vulval carcinomas, clear cell carcinoma of the ovary, low grade serous ovarian cancer, mixed mullarian tumours (carcinosarcoma), sarcomas of the female genital tract and granulosa cell tumours).

The role of immunotherapy is being defind in more common cancer types, however because of their rarity, the efficacy of immunotherapy for these cancers is poorly defined.

This protocol provides an important opportunity to establish whether the combination of nivolumab & ipilimumab has efficacy in these cancers.

1.2.4 Summary of Results from the Ipilimumab and Nivolumab programs

1.2.4.1 Preclinical Summary of Nivolumab combined with Ipilimumab

Preclinical data indicate that the combination of PD-1 and CTLA-4 receptor blockade may improve antitumor activity. In vitro combinations of nivolumab plus ipilimumab increase IFN-γ production 2- to 7-fold over either agent alone in a mixed lymphocyte reaction. Increased antitumor activity of the combination was also observed in 3 of 5 syngeneic murine cancer models. In a murine melanoma vaccine model, blockade with either CTLA-4 or PD-1 antibodies increased the proportion of CTLA-4 and PD-1-expressing CD4/CD8 tumour

infiltrating T effector cells, and dual blockade increased tumour infiltration of T effector cells and decreased intratumoral T regulatory cells, as compared to either agent alone(33).

Preclinically, a 4-week toxicity study of nivolumab in combination with ipilimumab conducted in cynomolgus monkeys demonstrated that the combination of nivolumab and ipilimumab resulted in dose-dependent gastrointestinal (GI) toxicity. Histologic findings included inflammatory changes in the large intestine, which increased in incidence and severity in a dose-dependent manner. GI toxicity/colitis was not observed in cynomolgus monkeys administered nivolumab alone, but was observed in monkeys receiving ipilimumab. Nivolumab in combination with ipilimumab was also associated with lymphoid hypocellularity of the cortex and/or medulla of the thymus and with acinar cell degranulation in the pancreas. Additional findings included interstitial mononuclear cell infiltrates in the kidneys, portal mononuclear cell infiltrates in the liver and myeloid hypercellularity in the bone marrow. Nivolumab in combination with ipilimumab at the high-dose level (i.e., 50 mg/kg and 10 mg/kg, respectively) was associated with the death of 1 animal, attributed to acute gastric dilatation without histopathological evidence of colitis upon pathology evaluation of the GI tract.

1.2.4.2 Summary of Safety

Ipilimumab Monotherapy

In MDX010-20, the ipilimumab monotherapy arm was administered 3 mg/kg ipilimumab every 3 weeks for four doses. In this arm, there were 79% drug related adverse events, with 21% being Grade 3/4 and 3/131 (2%) Grade 5. The most frequent adverse events of interest were rash (30%), pruritus (33%), diarrhea (33%), colitis (8%), endocrine disorders (9%), AST/ALT increased (2%), and hepatitis (1%). Any grade immune related adverse events were 60% and the Grade 3/4 immune related adverse events for the same cohort was 13% with the most frequent adverse events being diarrhea (5%), colitis (5%), rash (2%), and endocrine disorders (3%).

Additional details on the safety profile of ipilimumab, including results from other clinical studies, are also available in the ipilimumab IB.

Nivolumab Monotherapy

One study has contributed most to the clinical experience with nivolumab monotherapy in participants with melanoma and other solid malignancies. CA209003 is an ongoing Phase 1 open label, multiple dose escalation study in 304 Participants with select previously treated advanced solid tumours, including melanoma, RCC, NSCLC, colorectal cancer, and hormone-refractory prostate cancer. Participants received nivolumab at doses of 0.1, 0.3, 1, 3 or 10 mg/kg intravenously every 2 weeks, up to a maximum of 2 years of total therapy. As of 03-Jul-2012, a total of 107 melanoma participants were treated with nivolumab in the dose range of 0.1-10 mg/kg.

No maximal tolerated dose was identified in CA209003. The incidence, severity and relationship of AEs were generally similar across dose levels and tumour types. Nivolumab related AEs of any grade occurred in 72.4% of participants. The most frequent nivolumab related AEs occurring in ≥ 5% of participants included: fatigue (25.7%), rash (13.5%), diarrhea (11.8%), pruritus (10.2%), nausea (7.9%), decreased appetite (7.9%), hemoglobin decreased

(5.9%) and pyrexia (5.3%). The majority of events were low grade, with grade 3-4 drug related AEs observed in 14.8% of participants. The most common Grade 3-4 drug-related AEs occurring in ≥ 1% of participants were: fatigue (1.6%), lymphopenia (1.3%), abdominal pain (1%), diarrhea (1%), hypophosphatemia (1%) and pneumonitis (1%). At least one SAE was reported for 150 (49.3%) of the 304 participants at all dose levels. Grade 3-4 SAEs were reported for 23 participants (7.6%). Drug-related SAEs occurred in 11.5% of participants. Grade 3-4 drug-related SAEs reported in at least 2 participants included: diarrhea (3 participants, 1.0%), pneumonitis (3 participants, 1.0%), pneumonia (2 participants, 0.7%) and lipase increased (2 participants, 0.7%). Additional select treatment-related AEs have occurred with low frequency (< 5%) but are considered clinically meaningful, as they require greater vigilance for early recognition and prompt intervention. These AEs include: ALT increased (4.3%), AST increased (3.6%), pneumonitis (3.3%), hypothyroidism (3.0%), hyperthyroidism (1.3%), renal failure (1.0%), adrenal insufficiency (0.7%) and colitis (0.7%). Grade 3-4 events of pneumonitis were reported in 3 participants (1.0%) as described above (1 event was Grade 4). Grade 3 events of colitis, ALT increased, and AST increased were reported in 2 participants (0.7%) each. Grade 3 events of adrenal insufficiency, hyperthyroidism, and hypothyroidism were reported in 1 participant (0.3%) each. Treatment-related AEs leading to discontinuation were reported in 18 (5.9%) of the 304 treated participants on CA209003. The only events reported in more than 1 participant were pneumonitis (4 participants; 1.3%) and hepatitis (2 participants; 0.7%). There were 3 (1%) drug related deaths; each occurred after development of pneumonitis.

Preliminary new non-clinical safety findings of adverse pregnancy outcomes and infant losses in the absence of overt maternal toxicity have been reported.(34) The findings of increased late stage pregnancy loss and early infant deaths/euthanasia in nivolumab exposed pregnant monkeys suggest a potential risk to human pregnancy if there is continued treatment with nivolumab during pregnancy.

Additional details on the safety profile of nivolumab, including results from other clinical studies, are also available in the BMS-936558 (nivolumab) IB.

Nivolumab Combined with Ipilimumab

In the Phase 1 study CA209004, ascending doses of nivolumab have been studied concomitantly with ascending doses of ipilimumab in participants with unresectable or metastatic melanoma. In each arm in this multi-arm study, ipilimumab was administered once every 3 weeks for 4 doses with nivolumab administered once every 3 weeks for 8 doses. Starting at week 24, ipilimumab and nivolumab were administered once every 12 weeks for 8 doses. The three initial dose-escalation cohorts consisted of Cohort 1 (nivolumab 0.3 mg/kg plus ipilimumab 3 mg/kg; n=14), Cohort 2 (nivolumab 1.0 mg/kg plus ipilimumab 3 mg/kg; n=17) and Cohort 3 (nivolumab 3.0 mg/kg plus ipilimumab 3 mg/kg; n=6). Later, the study was amended to include Cohort 2a which evaluated nivolumab 3 mg/kg plus ipilimumab 1 mg/kg (n-16).

The following DLTs were observed in Cohort 1 - Grade 3 elevated AST/ALT (1 participant); in Cohort 2 - Grade 3 uveitis (1 participant) and Grade 3 elevated AST/ALT (1 participant) and in Cohort 3 - Grade 4 elevated lipase (2 participants) and Grade 3 elevated lipase (1

participant). Based on these data, Cohort 2 was identified as the maximum tolerated dose (MTD) and Cohort 3 exceeded the MTD.

As of 15-Feb-2013, a total of 53 melanoma participants were treated with nivolumab combined with ipilimumab in CA209004 across cohorts 1, 2, 2a, and 3. At least one AE regardless of causality has been reported in 98% of participants treated. The most common (reported at > 10% incidence) treatment related AEs (any Grade %; Grade 3-4 %: 93; 53) are rash (55; 4), pruritus (47; 0), vitiligo (11; 0), fatigue (38; 0), pyrexia (21, 0), diarrhea (34; 6), nausea (21, 0), vomiting (11, 2), ALT increased (21; 11), AST increased (21; 13), lipase increased (19; 13), amylase increased (15, 6), headache (11, 0), and cough (13, 0).

The majority of AEs leading to discontinuation (regardless of causality) were Grade 3 or 4 (reported in 11 of 53 participants, 21%). Grade 3 events included lipase increased, ALT increased, AST increased, troponin I increased, colitis, diverticular perforation, pancreatitis, tachycardia, renal failure acute, choroiditis, autoimmune disorder, and pneumonitis. One participant each discontinued due to Grade 4 events of blood creatinine increased and AST increased. No drug-related deaths were reported.

Adverse Event Management Algorithms

Because of the potential for clinically meaningful nivolumab or ipilimumab related AEs requiring early recognition and prompt intervention, management algorithms have been developed for suspected pulmonary toxicity, GI, hepatotoxicity, endocrinopathy, skin toxicity, neurological toxicity and renal toxicity. Prompt interventions are recommended according to the management algorithms and in addition include ophthalmologic evaluations for any visual symptoms in order to evaluate for nivolumab or ipilimumab related uveitis.

The recommendations are to follow the BMS-936558 (nivolumab) adverse event algorithms and not the ipilimumab IB algorithms.

The algorithms recommended for utilization in CA209-538 are contained in Appendix 4.

As of 03-Apr-2013, three participants out of approximately 1200 patients on nivolumab clinical trials have developed opportunistic infections (2 cases of Aspergillus pneumonia, and 1 case of Pneumocystis jiroveci pneumonia) after receiving prolonged treatment with high dose steroids for nivolumab-related adverse events. Details of these cases are available in the Investigator Brochure. Because of the potential for opportunistic infections with prolonged high dose corticosteroids administration, the following recommendations should be considered for participants with inflammatory events expected to require more than 4 weeks of corticosteroids or other immunosuppressants to manage the adverse event:

- Antimicrobial/antifungal prophylaxis per institutional guidelines to prevent opportunistic infections such as Pneumocystis jiroveci and fungal infections.
- Early consultation with an infectious disease specialist should be considered. Depending on the presentation, consultation with a pulmonologist for bronchoscopy or a gastroenterologist for endoscopy may also be appropriate.
- In patients that develop recurrent adverse events in the setting of ongoing or prior immunosuppressant use, an opportunistic infection should be considered in the differential diagnosis.

Additional details on the safety of nivolumab, including results from other clinical studies, are available in the IB.

1.2.4.3 Summary of Clinical Activity

Ipilimumab Monotherapy

In melanoma, a completed Phase 3 study (MDX010-20) has demonstrated a clinically meaningful and statistically significant survival benefit in pre-treated advanced melanoma. The study compared the overall survival (OS) of ipilimumab plus a melanoma-specific vaccine (gp100) to that of gp100 alone. A second comparison defined the OS of ipilimumab alone vs gp100 alone. Both comparisons demonstrated statistically significant improvements in OS (p = 0.0004 and 0.0026, respectively). The 1-year survival for the two ipilimumab-containing groups, respectively, was 44% and 46% respectively, compared to 25% for the gp100 control group. The 2-year survival was 22%, 24% and 14% respectively. The median survival was 10, 10.1, and 6.4 months, for ipilimumab plus gp100, ipilimumab monotherapy, and gp100 monotherapy, respectively.

Nivolumab Monotherapy

In CA209003, the clinical activity of nivolumab was demonstrated in a variety of tumour types, including melanoma, RCC, and NSCLC. Clinical activity was noted across a range of doses (0.1 mg/kg, 0.3 mg/kg, 1 mg/kg, 3 mg/kg, 10 mg/kg).

In CA209003, as of the clinical cut-off date of 03-Jul-2012, a total of 304 participants with melanoma, RCC, and NSCLC have been evaluated for clinical activity. A response of either CR or PR, as determined by investigator assessed tumour evaluations based on modified RECIST 1.1, has been reported at all dose levels. No responses (CR or PR) have been reported in participants with colorectal carcinoma or castrate-resistant prostate cancer.

Among 106 patients with advanced melanoma who received nivolumab and were evaluable for response, the preliminary objective response rates were 6/17 (35%), 5/18 (28%), 11/34 (32%), 7/17 (41%), and 4/20 (20%) for melanoma participants treated at 0.1, 0.3, 1, 3, and 10 mg/kg, respectively. Duration of response range from 3.6 to 11.2, 1.8 to 9.2, 1.9 to 24.9, 9.2 to 22.4, and 17.0 to 25.7 months in the melanoma participants treated at 0.1, 0.3, 1, 3, and 10 mg/kg, respectively. Stable disease ≥ 24 weeks occurred in an additional 1/18 (6%), 4/34 (12%), 1/17(6%) melanoma participants at 0.3, 1, and 3 mg/kg, respectively. Finally, the PFS-24 week was 41%, 33%, 48%, 55%, and 30% in melanoma participants treated at 0.1, 0.3, 1, 3, and 10 mg/kg, respectively.

Nivolumab Combined with Ipilimumab

As of the 15-Feb-2013 clinical cut-off in CA209004, of the 52 participants evaluable for response, 21 participants (40%) had an objective response by modified World Health Organization (mWHO) criteria. In an additional 2 participants (4%) there was an unconfirmed objective response. In Cohort 1 (0.1 mg/kg nivolumab + 3 mg/kg ipilimumab), 3 out of 14 evaluable participants had an objective response by mWHO (21%); 1 CR and 2 PRs with an additional PR by immune-related mWHO criteria (irPR) (33). In Cohort 2 (1 mg/kg nivolumab + 3 mg/kg ipilimumab), 9 out of 17 evaluable participants had an objective response by mWHO (53%; 3 CRs (18%), 6 PRs (35%) with two additional participants experiencing immune-

related SD (irSD). In Cohort 2a (3 mg/kg nivolumab + 1 mg/kg ipilimumab), 6 out of 15 response evaluable participants had an objective response rate by mWHO (40%; 1 CR (7%), 5 PRs (33%) with 2 additional uPRs (13%) and 2 irSDs and 1 irPR). In Cohort 3 (3 mg/kg nivolumab + 3 mg/kg ipilimumab), 3 out of 6 evaluable participants had an objective response by mWHO (50%; 3 PRs (50%) with 1 additional irPR and 1 irSD.

Preliminary analysis revealed 16 of the 52 evaluable participants (31%) had > 80% reduction in the size of target tumour lesions by the week 12 evaluation. This is compared to < 2% for 3 mg/kg ipilimumab monotherapy based on CA184020 (N=540) and < 3% for nivolumab monotherapy based on CA209003 (N=94, 0.1-10 mg/kg).

1.2.4.4 Clinical Pharmacology Summary

Ipilimumab Monotherapy

Ipilimumab has a terminal half life of approximately 15.4 days. The expected in vivo degradation of monoclonal antibodies is to small peptides and amino acids via biochemical pathways that are independent of cytochrome P450 enzymes.

The population PK of ipilimumab was studied with 785 participants and demonstrated that PK of ipilimumab is linear and exposures are dose proportional across the tested dose range of 0.3 to 10 mg/kg, and the model parameters are time invariant. Upon repeated dosing of ipilimumab, administered every three weeks, minimal systemic accumulation was observed by an accumulation index of 1.5-fold or less and ipilimumab steady-state concentrations were achieved by the third dose. The ipilimumab clearance of 16.8 mL/h from population PK analysis is consistent with that determined by PK analysis. The terminal half-life (T-HALF) and Vss of ipilimumab calculated from the model were 15.4 days, and 7.47 L, which are consistent with that determined by non-compartmental analysis (NCA). Volume of central (Vc) and peripheral compartment were found to be 4.35 L and 3.28 L, respectively, suggesting that ipilimumab first distributes into plasma volume and subsequently into extracellular fluid space. Clearance of ipilimumab and Vc were found to increase with increase in body weight. Nevertheless, there was no significant increase in exposure with increase in body weight when dosed on a mg/kg basis, supporting dosing of ipilimumab based on a weight normalized regimen. Additional details are provided in investigator brochure.

Nivolumab Monotherapy

Single-dose pharmacokinetics (PK) of nivolumab was evaluated in participants with multiple tumour types in MD1106-01 whereas multiple dose PK is evaluated in participants in CA209003. In addition, a preliminary population pharmacokinetic (PPK) model has been developed with data from ± 350 participants from MDX1106-01, MDX1106-02 and CA209003.

Single-dose PK of nivolumab was evaluated in 39 participants with multiple tumour types in study MDX1106-01 in the dose range of 0.3 to 10 mg/kg. The median Tmax across single doses ranged from 1.6 to 3 hours with individual values ranging from 0.9 to 7 hours. The PK of nivolumab is linear in the range of 0.3 to 10 mg/kg with dose proportional increase in Cmax and AUC(INF) with low to moderate inter-participant variability observed at each dose level (i.e., CV ranging from 7 to 45%). Geometric mean clearance (CL) after a single intravenous (IV) dose ranged from 0.13 to 0.19 mL/h/kg, while mean volume of distribution (Vz) varied

between 83 to 113 mL/kg across doses. The mean terminal T-HALF of nivolumab is 17 to 25 days, which is consistent with half life of endogenous IgG4, indicating that the elimination mechanism of nivolumab may be similar to IgG4. Both elimination and distribution of nivolumab appear to be independent of dose in the dose range studied. Additional details are provided in the Investigator Brochure.

A preliminary PK model was developed by nonlinear mixed effect modeling using data from 350 participants from MDX1106-01, MDX1106-02 and CA209003. The body weight normalized dosing produces approximately constant trough concentrations over a wide range of body weight, and hence is appropriate for future clinical trials of nivolumab. Clearance of nivolumab is similar in all tumour types studied and is independent of dose range studied (0.1 to 10 mg/kg).

1.2.5 Rationale for the Study Design

1.2.5.1 Rationale for Nivolumab combined with Ipilimumab and the Dose and Schedule

The combination of nivolumab and ipilimumab was chosen because of preclinical and clinical evidence suggesting synergy between nivolumab and ipilimumab. While PD-1 and CTLA-4 are both co-inhibitory molecules, evidence suggests that they use distinct mechanisms to limit T cell activation. Preliminary indirect data from peripheral T cell assessments suggest that a given T-cell checkpoint inhibitor may modulate host immune cell phenotype rendering them more susceptible to alternate checkpoint inhibitors and thereby enhancing anti-tumour activity. Specifically, nivolumab increased peripheral CTLA-4+ and regulatory T cells in participants without clinical response in CA209006. In a preclinical melanoma model, anti-CTLA-4 therapy increased PD-1+, PD-L1+ and CTLA-4+ tumour infiltrating T cells(32). In addition, in the Phase 2 ipilimumab monotherapy study CA184004, increases in tumour infiltrating lymphocytes (TILs) and interferon-γ-inducible genes were observed following treatment with ipilimumab, and PD-L1 positive tumour cells co-localize with both TILs and interferon-γ expression in metastatic melanoma(36, 37).

The preliminary clinical evidence has demonstrated a higher frequency of patients with substantial tumour burden reduction for the combination of nivolumab and ipilimumab. Improved overall survival associated with substantial tumour burden reduction has been noted with immunotherapies. For instance, improved overall survival has been noted in metastatic melanoma participants obtaining a complete response to IL-2(38). If this observation is also applicable to treatment with nivolumab combined with ipilimumab then there could also be the potential for large improvements in overall survival compared to ipilimumab.

Dose and Schedule Rationale

This combination was initially evaluated in melanoma and dose finding was undertaken in protocol CA209004. In that study, the 3 mg/kg nivolumab and 3 mg/kg ipilimumab cohort exceeded the maximum tolerated dose per protocol. In CA209004, while both Cohort 2 (1 mg/kg nivolumab + 3 mg/kg ipilimumab) and Cohort 2a (3 mg/kg nivolumab + 1 mg/kg ipilimumab) had similar clinical activity, a dose of 3 mg/kg of ipilimumab every 3 weeks for a total of four doses and 1 mg/kg nivolumab every 3 weeks for four doses followed by nivolumab 3mg/kg every 2 weeks until progression was chosen. Exposure-response analysis of nivolumab

monotherapy across dose ranges of 1 mg/kg to 10 mg/kg reveals similar clinical activity while exposure-response analysis of 0.3 mg/kg, 3 mg/kg, and 10 mg/kg of ipilimumab monotherapy have demonstrated increasing activity with increase in dose in the phase 2 study CA184022 (37).

Consequently 3 mg/kg of nivolumab and 3 mg/kg of ipilimumab was selected for CA209067, the recently reported randomized study of combination therapy with nivolumab and ipilimumab ve monotherapy with each agent (40). In that study the combination was found to be highly effective and moreso than either monotherapy arm, however therapy was associated with appreciable toxicity, much of which reflects toxicity previously seen with ipilimumab. Consequently we have selected a reduced dose of ipilimumab with the intention of improving the tolerability fo the regimen.

Given the uncertainty of whether the ipilimumab administered past week 12 contributes to the clinical benefit and the fact that the approved schedule for ipilimumab is every 3 weeks for a total of four doses in the FDA and EMA approved label dosing section, ipilimumab will only be administered every 3 weeks for a total of 4 doses. Nivolumab monotherapy treatment every two weeks until progression was studied in CA209003 and is implemented program-wide across the nivolumab monotherapy Phase 3 registrational trials. Thus starting at week 12, which is after the completion of the four doses of combined nivolumab and ipilimumab, nivolumab would continue to be administered every two weeks until progression in order to maintain consistency with the nivolumab monotherapy program.

Following the emergence of the COVID-19 pandemic, an alternative dosing regime for Nivolumab monotherapy during the maintenance period has been proposed. The alternative is Nivolumab 4-weekly at 480mg (regardless of participant weight) until progression. This dosing is the current TGA approved dose in treating Metastatic Melanoma. If this option is to be utilised, prior approval must be sought from the sponsor.

1.2.5.2 Rationale for Permitting Continued Treatment in Select Cases of Progressive Disease

Accumulating clinical evidence indicates some participants treated with immune system stimulating agents may develop progression of disease (by conventional response criteria) before demonstrating clinical objective responses and/or stable disease. This phenomenon was observed in approximately 10% of participants in the Phase 1 study of nivolumab and also with ipilimumab monotherapy(34). Two hypotheses have been put forth to explain this phenomenon. First, enhanced inflammation within tumours could lead to an increase in tumour size which would appear as enlarged index lesions and as newly visible small non-index lesions. Over time, both the malignant and inflammatory portions of the mass may then decrease leading to overt signs of clinical improvement. Alternatively, in some individuals, the kinetics of tumour growth may initially outpace anti-tumour immune activity. With sufficient time, the anti-tumour activity will dominate and become clinically apparent. Therefore, participants will be allowed to continue study therapy after initial investigator-assessed RECIST 1.1 defined progression if they are assessed to be deriving clinical benefit and tolerating study drug (Section 4.3.7). Such participants must discontinue study therapy upon evidence of further progression.

1.2.5.3 Predictive Biomarker analysis: whether a common predictive biomarker or immune signature can be identified in responding patients that can occur irrespective of tumor type.

In non-melanoma cancers, the efficacy of anti PD-1 monotherapy (nivolumab (BMS) or pembrolizumab (Merck) has been investigated in a variety of clinical trials (41-46) and preliminary indications are that a response rate of 20-30% or better can be achieved. Combinations with other checkpoint inhibitors, including anti-CTLA4, are currently underway and will be reported in the near future. Even early in the course of clinical development two observations can already be made with some confidence: (i) blocking PD-1/PD-L1, including the use of nivolumab, is a highly active immunotherapeutic approach across a range of different cancer types, (ii) the addition of anti-CTLA4 increases efficacy in melanoma, and this is likely to be the case for other cancer types as well. In addition, emerging biomarker analyses point to patient & tumour characteristics that are associated with clinical benefit (37, 47). While evaluating PD-L1 expression has some correlation with clinical benefit, it is relatively non-specific, with clear responses documented in patients whose tumours were PD-L1 negative. PD-L1 alone therefore cannot be relied on as a robust predictive marker (41).

Interpatient variability: despite the huge promise shown with immunotherapeutics, it is clear that responses vary between patients from complete objective responses (CR) to no apparent effect (disease progression (PD)). The causes of this variability are somewhat defined and may include: (i) immune ignorance (failure of the immune system to recognise tumour), or (ii) immune tolerance (unresponsiveness of the immune system to tumour despite the capacity to elicit an immune response). This tolerance may, in-turn, be central or peripheral (48, 49). Furthermore the concepts of immune escape, immunoediting and immunesubversion have been developed to describe either the selection of antigen-escape variants or cancer-dependent regulatory mechanisms that interfere with effective cancer elimination (5). The contribution of these various mechanisms can be interrogated, and we propose to do so using a variety of biomarker assays described in detail below. These can identify specific interactions between cells of the immune system and cancer, as well as characterise the inflammatory attributes of the tumour micro-environment (TME)(37, 47). They include the characterisation of cellular and molecular mechanisms that down-regulate effective immune rejection of cancer (50). In addition, there are likely to be contributions from host factors that affect the quality of the immune response such as inherited susceptibilities & polymorphisms (51), acquired immunodeficiency or hyperactivity and environmental factors such as the host microbiome (52).

1.3 Overall Risk/Benefit Assessment

There continues to be a significant unmet need for patients with these rare cancers. Nivolumab monotherapy has demonstrated clinical activity across several tumour types, including advanced prior treated melanoma, with objective response rates of 20 - 41% in 106 melanoma participants treated at various dose levels in CA209003. Nivolumab has also demonstrated a manageable safety profile. The most common AEs included fatigue, rash, pruritus, diarrhea, and nausea.

The combination of nivolumab and ipilimumab has the potential for increased benefit compared to both ipilimumab monotherapy and nivolumab monotherapy. In the CheckMate 067 melanoma trial (ClinicalTrials.gov number, NCT01844505) the median progression-free

survival was 11.5 months (95% confidence interval [CI] 8.9 to 16.7) with nivolumab plus ipilimumab, as compared with 2.9 months (95% CI, 2.8 to 3.4) with ipilimumab and 6.9 months with nivolumab alone. However treatment-related adverse events of grade 3 or 4 occurred in 16.3% of the patients in the nivolumab group, 55.0% of those in the nivolumab-plus-ipilimumab The most common (reported at > 10% incidence) treatment related AEs are fatigue, rash, pruritus, diarrhea, lipase increased, pyrexia, ALT increase, AST increased, amylase increased and vitiligo. Many of the Grade 3-4 adverse events associated with the nivolumab combined with ipilimumab were laboratory in nature and the clinically significant AEs have been manageable and reversible following intervention dose delays or with systemic steroid treatment (40). The promising clinical activity of nivolumab combined with ipilimumab in participants with advanced melanoma in combination with the manageable safety profile and the lack of approved survival-prolonging agents for a many of the rare cancer populations supports the evaluation of nivolumab combined with ipilimumab in participants with these cancer types.

2 ETHICAL CONSIDERATIONS

2.1 Good Clinical Practice

This study will be conducted in accordance with the Declaration of Helsinki and any subsequent amendments, the ICH Guidelines for Good Clinical Practice (CPMP/ICH/153/95) annotated with TGA comments (July 2000), the NHMRC National Statement on Ethical Conduct in Research involving Humans (2007) and any subsequent updates, the policies and procedures of ONJCRI and any applicable local guidelines.

The study will be conducted in compliance with the protocol. The protocol and any amendments and the participant informed consent form will receive Human Research Ethics Committee (HREC) approval/favorable opinion prior to initiation of the study at each site.

All potential serious breaches must be reported to ONJCRI and BMS immediately. A serious breach is a breach of the conditions and principles of GCP in connection with the study or the protocol, which is likely to affect, to a significant degree, the safety or physical or mental integrity of the participants of the study or the scientific value of the study.

Personnel involved in conducting this study will be qualified by education, training, and experience to perform their respective tasks.

This study will not use the services of study personnel where sanctions have been invoked or where there has been scientific misconduct or fraud (e.g., loss of medical licensure, debarment).

2.2 Human Research Ethics Committee

Before study initiation, the investigator must have written and dated approval/favorable opinion from the HREC for the protocol, consent form, participant recruitment materials such as advertising materials, and any other written information that will be provided to participants.

The investigator or BMS should provide the HREC with reports, updates and other information (e.g., expedited safety reports, amendments, and administrative letters) according to regulatory requirements or institution procedures.

2.3 Informed Consent

Investigators must ensure that participants are clearly and fully informed about the purpose, potential risks, and other critical issues regarding clinical studies in which they volunteer to participate.

The site will be provided with an appropriate informed consent form which will include all elements required by ICH, GCP and applicable local regulatory requirements. The informed consent form will adhere to the ethical principles that have their origin in the Declaration of Helsinki.

Investigators must:

1. Allow time necessary for participant or participant's legally acceptable representative to inquire about the details of the study.
2. Obtain an informed consent signed and personally dated by the participant or the participant's legally acceptable representative and by the person who conducted the informed consent discussion.
3. Obtain the HREC written approval/favourable opinion of the written informed consent form and any other information to be provided to the participants, prior to the beginning of the study, and after any revisions are completed for new information.
4. If informed consent is initially given by a participant’s legally acceptable representative or legal guardian, and the participant subsequently becomes capable of making and communicating his or her informed consent during the study, consent must additionally be obtained from the participant.
5. Revise the informed consent whenever important new information becomes available that is relevant to the participant's consent. The investigator, or a person designated by the investigator, should fully inform the participant or the participant's legally acceptable representative or legal guardian, of all pertinent aspects of the study and of any new information relevant to the participant's willingness to continue participation in the study. This communication should be documented.

The consent form must also include a statement that the sponsor and regulatory authorities may require direct access to participant records.

The rights, safety, and well-being of the study participants are the most important considerations and should prevail over interests of science and society.

2.4 Confidentiality and privacy

The participants will be allocated a trial registration number. Information linking identifying participant information and registration number will be maintained in a secure location separate from the participant database. Analysis of trial-related data and all ongoing queries will be via patient trial registration number only. Each institution will maintain a list of its own trial participants. Data will be analysed by registration number and initials. Samples for correlative studies will also be analysed only by the trial registration number.

All information regarding trial participants must be treated in strict confidence. Data that identify any trial participant must not be revealed to anyone not directly involved in the trial or the clinical care of that participant. An exception is where de-identified source data verification must be sent to the ONJCRI for trial management to be included in source document verification. In this instance, the records may be inspected by (a) a representative of ONJCRI for the purposes of source document verification or quality audit as stipulated in the ICH GCP Guidelines, or (b) a representative of a government regulatory authority for the purposes of official inspection. Records must be made available for inspection on the understanding that all information relating to trial participants will be treated in strict professional confidence.

2.5 Ongoing Safety Monitoring

A Trial Management Committee will be put in place for this trial.

The TMC will provide:

- Medical oversight to the clinical trial
- Provide safety oversight
- Review of protocol amendments
- In the event of a significant incidence of SAEs, give consideration to amending the trial
- Assess trial progress
- An independent medical monitor has been appointed by ONJCRI for this study. This person will be consulted to review SAEs for causality, and provide independent medical advice to the study team where required to ensure the best outcome for the patient in line with the protocol and GCP guidelines.

  Lead medical monitor contact details:

  Dr David Pook
  david.pook@monash.edu

3 INVESTIGATIONAL PLAN

3.1 Study Design and Duration

This is a phase 2, open-label trial of nivolumab combined with ipilimumab in adult (≥ 18 years) participants with rare cancers. Participants must have a diagnosis of a rare cancer in the three proposed tumor streams: Upper GI malignancies (Cholangiocarcinoma/ duodenal carcinoma); Neuroendocrine tumours (Inc. pancreatic, bronchial and intestinal carcinoid tumours) and Rare Gynaecological tumors (including but will not be limited to: vaginal or vulval carcinomas, clear cell carcinoma of the ovary, low grade serous ovarian cancer, mixed Mullarian tumours (carcinosarcoma), sarcomas of the female genital tract and granulosa cell tumours) who are not suitable for, or if declining established standard therapies.

Participants will be treated with: Nivolumab: 3 mg/kg and ipilumumab: 1 mg/kg concurrently Q3W for 4 doses followed by nivolumab at 3mg/kg every 2 weeks until progression (up to 48 total doses of nivolumab)

In exceptional circumstances, where participants are being treated with Nivolumab monotherapy, there will be an option to be treated with Q4W Nivolumab 480 mg. If the treating investigator wishes to utilise this option, prior approval must be sort by the sponsor.

One cycle of treatment will be defined as six weeks (or 8 weeks in the case of exceptional circumstances where Nivolumab is administered 4 weekly). Dose reductions will be not be allowed for any of the treatments except in the case of a change in weight ≤ or ≥ 10% of the baseline weight. On-study tumour assessments will begin 12 weeks from registration, at 18 weeks, and every 12 weeks thereafter until disease progression or treatment discontinuation, whichever occurs later. Treatment beyond initial investigator-assessed RECIST 1.1-defined progression is permitted if the participant has investigator-assessed clinical benefit and is tolerating study drug.

The study design schematic is presented in Figure 3.1-1

Figure 3.1-1: Study Design Schematic

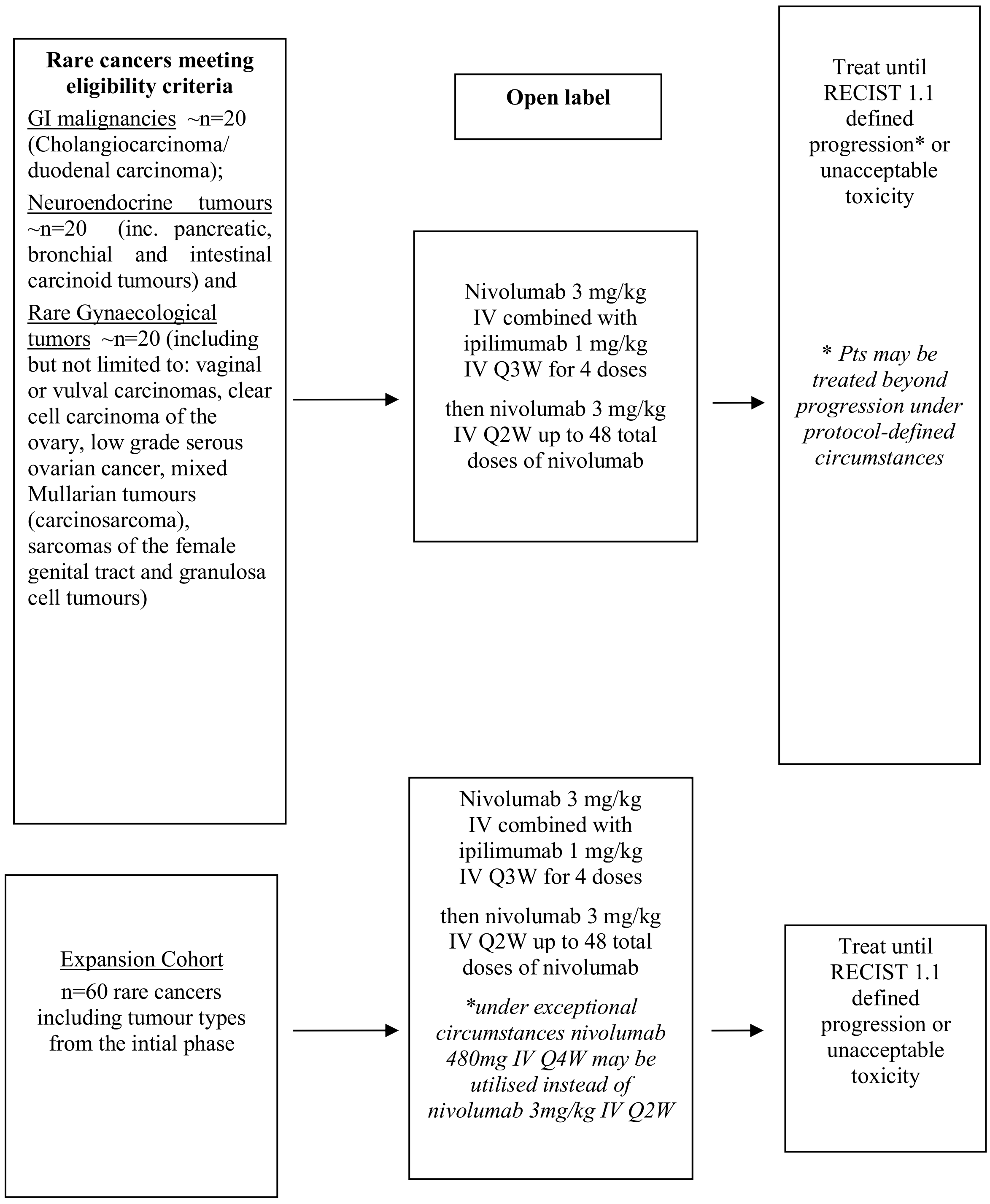

This study will consist of three phases: screening, treatment, and follow-up.

Screening Phase:

- Begins by establishing the participant's initial eligibility and signing of the informed consent form (ICF).
- Tumour tissue from an unresectable or metastatic site of disease must be provided for biomarker analyses. If an insufficient amount of tumour tissue from an unresectable or metastatic site is available prior to the start of the screening phase, participants must consent to allow the acquisition of additional tumour tissue for performance of biomarker analyses

Treatment Phase:

- On-study laboratory assessments should be drawn within 72 hours prior to dosing.
- On-study optional biopsies may be collected as per Table 5.1-2.
- Adverse event assessments should be documented at each clinic visit. WOCBP must have a pregnancy test during week 1 and week 4 for cycles 1-2 and week 1 and week 5 of starting from cycle 3. Table 5.1-2 and Table 5.1-3
- Blood samples will be collected according to the schedule in Table 5.1-1
- Study drug dosing may be delayed for toxicity. See Section 4.3.2

For the first 2 cycles (12 weeks);

- Nivolumab and ipilimumab are administered every 3 weeks for 4 doses Table 4.3-1

Starting cycle 3;

- Nivolumab 3mg/kg is administered every 2 weeks Table 4.3-2 OR under exceptional circumstances, it may be administered every 4 weeks at 480mg (regardless of participant weight)
- Study drug dose may be delayed for toxicity. See Section 4.3.2
- Treated participants will be evaluated for response according to the RECIST 1.1 guidelines beginning 12 weeks (± 1 week) after registration, at 18 weeks (± 1 week) and continuing and then every 12 weeks (± 1 week) until disease progression or treatment discontinuation, whichever occurs later.
- This phase ends when the participant is discontinued from study therapy. For a complete list of reasons for treatment discontinuation, see Section 3.5.

Follow-Up Phase

- Begins when the decision to discontinue a participant from study therapy is made (no further treatment with study therapy).
- Two follow-up visits include collection of Safety samples (table
- Participants who discontinue treatment for reasons other than tumour progression will continue to have tumour assessments beginning 12 weeks (± 1 week) after at 18 weeks (± 1 week) and continuing and then every 12 weeks (± 1 week) thereafter until documented tumour progression.
- Participants will be followed for drug-related toxicities until these toxicities resolve, return to baseline or are deemed irreversible. All adverse events will be documented for a minimum of 100 days after last dose.
- After completion of the first two safety follow-up visits, participants will be followed every 3 months for survival.

Survival is only an exploratory endpoint of this study because numbers will be insufficient for meaningful PFS & OS estimates based on tumour types. Nonetheless exploratory analysis of survival will be undertaken so post-study follow-up will be important. Consequently participants who discontinue study drug must continue to be followed for collection of outcome and/or survival follow-up data as required and in line with section 3.1 until death or the conclusion of the study.

3.2 Post Study Access to Therapy

At the conclusion of the study, participants who continue to demonstrate clinical benefit will be eligible to receive study drug. Study drug will be provided via an extension of the study, a rollover study requiring approval by responsible health authority and ethics committee or through another mechanism at the discretion of BMS. BMS reserves the right to terminate access to study drug if any of the following occur: a) the marketing application is rejected by responsible health authority; b) the study is terminated due to safety concerns; c) the participant can obtain medication from a government sponsored or private health program; or d) therapeutic alternatives become available in the local market.

3.3 Study Population

For entry into the study, the following criteria MUST be met.

3.3.1 Inclusion Criteria

1. Signed Written Informed Consent

a) Participants must have signed and dated an IRB/IEC approved written informed consent form in accordance with regulatory and institutional guidelines. This must be obtained before the performance of any protocol related procedures that are not part of normal participant care

b) Participants must be willing and able to comply with scheduled visits, treatment schedule, laboratory testing, and other requirements of the study

2. Target Population

a) Histologically confirmed Upper GI malignancies (Cholangiocarcinoma/ duodenal carcinoma – collect MSI status (ie. Known/unknown)); Neuroendocrine tumours (Inc. pancreatic, bronchial and intestinal carcinoid tumours) and Rare Gynaecological tumours (including but will not be limited to: vaginal or vulval carcinomas, clear cell carcinoma of the ovary, low grade serous ovarian cancer, mixed Mullarian tumours (carcinosarcoma), sarcomas of the female genital tract and granulosa cell tumours).

b) Eastern Cooperative Oncology Group (ECOG) performance status of ≤ 1

c) Prior systemic therapy is permitted if it was completed at least 4 weeks prior to enrolment, and all related adverse events have either returned to baseline or stabilized or participants are not suitable for, or if declining established standard therapies.

d) Prior radiotherapy must have been completed at least 2 weeks prior to study drug administration.

e) Measurable disease by CT or MRI per RECIST 1.1 criteria section 5.4.3.1

f) Tumour tissue from an unresectable or metastatic site of disease must be provided for biomarker analyses. If an insufficient amount of tumour tissue from an unresectable or

metastatic site is available prior to the start of the screening phase, participants must consent to allow the acquisition of additional tumour tissue for performance of biomarker analyses.

g) Screening laboratory values must meet the following criteria and should be obtained within 14 days prior to registration:

   i) WBC ≥ 2000/μL

   ii) Neutrophils ≥ 1500/μL

   iii) Platelets ≥ 100 x10^3/μL

   iv) Hemoglobin > 9.0 g/dL

   v) Serum creatinine ≤ 1.5 x ULN or creatinine clearance (CrCl) ≥ 40 mL/min (using the Cockcroft-Gault formula):

   $$\text{Female CrCl} = \frac{(140 - \text{age in years}) \times \text{weight in kg} \times 0.85}{72 \times \text{serum creatinine in mg/dL}}$$

   $$\text{Male CrCl} = \frac{(140 - \text{age in years}) \times \text{weight in kg} \times 1.00}{72 \times \text{serum creatinine in mg/dL}}$$

   vi) AST/ALT ≤ 3 x ULN

   vii) Total Bilirubin ≤ 1.5 x ULN (except participants with Gilbert Syndrome, who can have total bilirubin < 3.0 mg/dL).

h) Participant Re-enrolment: This study permits the re-enrolment of a participant that has discontinued the study as a pre-treatment failure (i.e., participant has not been treated) after obtaining agreement from the medical monitor prior to re-enrolling a participant. If re-enrolled, the participant must be re-consented.

3. Age and Reproductive Status

a) Men and women, ≥ 18 years of age

b) Women of childbearing potential (WOCBP) must use method(s) of contraception as indicated in Appendix 5. For a teratogenic study drug and/or when there is insufficient information to assess teratogenicity (preclinical studies have not been done), a highly effective method(s) of contraception (failure rate of less than 1% per year) is required. The individual methods of contraception and duration should be determined in consultation with the investigator. WOCBP must follow instructions for birth control when the half-life of the investigational drug is greater than 24 hours, contraception should be continued for a period of 30 days plus the time required for the investigational drug to undergo five half-lives. The half-life of BMS- 936558 and ipilimumab is up to 25 days and 18 days, respectively. WOCBP should use an adequate method to avoid pregnancy for 23 weeks (30 days plus the time required for nivolumab to undergo five half-lives) after the last dose of investigational drug.

c) Women must have a negative serum or urine pregnancy test (minimum sensitivity 25 IU/L or equivalent units of HCG) within 24 hours prior to the start of investigational product.

d) Women must not be breastfeeding

e) Men who are sexually active with WOCBP must use any contraceptive method with a failure rate of less than 1% per year The investigator shall review contraception methods and the time period that contraception must be followed. Men that are sexually active with WOCBP must follow instructions for birth control when the half-life of the investigational drug is greater than 24 hours, contraception should be continued for a period of 90 days plus the time required for the investigational drug to undergo five half-lives. The half-life of nivolumab and ipilimumab is up to 25 days and 18 days, respectively. Men who are sexually active with WOCBP must continue contraception for 31 weeks (90 days plus the time required for nivolumab to undergo five half-lives) after the last dose of investigational drug.

f) Women who are not of childbearing potential (i.e., who are postmenopausal or surgically sterile; see Section 3.3.3 for the definition of WOCBP) and azoospermic men do not require contraception.

3.3.2 Exclusion Criteria

1. Target Disease Exceptions

a) Active brain metastases or leptomeningeal metastases. Participants with brain metastases are eligible if these have been treated and there is no magnetic resonance imaging (MRI - except where contraindicated in which CT scan is acceptable) evidence of progression for at least 8 weeks after treatment is complete and within 28 days prior to first dose of study drug administration. Cases should be discussed with the medical monitor. There must also be no requirement for immunosuppressive doses of systemic corticosteroids (> 10 mg/day prednisone equivalents) for at least 2 weeks prior to study drug administration.

2. Medical History and Concurrent Diseases

a) Prior combination treatment directed against the PD-1/PDL1 axis (anti-PD-1, anti-PD-L1, anti-PD-L2), and anti-CTLA-4 antibody. Prior monotherapy with these agents or other immune-stimulating/regulating agents is permitted.

b) Any serious or uncontrolled medical disorder that, in the opinion of the investigator, may increase the risk associated with study participation or study drug administration, impair the ability of the participant to receive protocol therapy, or interfere with the interpretation of study results.

c) Prior malignancy active within the previous 3 years except for locally curable cancers that have been apparently cured, such as basal or squamous cell skin cancer, superficial bladder cancer, or carcinoma in situ of the prostate, cervix, or breast.

d) Participants with active, known or suspected autoimmune disease. Participants with vitiligo, type I diabetes mellitus, residual hypothyroidism due to autoimmune condition only requiring hormone replacement, psoriasis not requiring systemic treatment, or conditions not expected to recur in the absence of an external trigger are permitted to enrol.

e) Participants with a condition requiring systemic treatment with either corticosteroids (> 10 mg daily prednisone equivalents) or other immunosuppressive medications within 14 days of study drug administration. Inhaled or topical steroids, and adrenal replacement doses > 10 mg daily prednisone equivalents are permitted in the absence of active autoimmune disease.

3. Physical and Laboratory Test Findings

a) Any positive test result for hepatitis B virus or hepatitis C virus indicating acute or chronic infection

b) Known history of testing positive for human immunodeficiency virus (HIV) or known acquired immunodeficiency syndrome (AIDS).

4. Allergies and Adverse Drug Reaction

a) History of allergy to study drug components.

b) History of severe hypersensitivity reaction to any monoclonal antibody.

5. Sex and Reproductive Status

a) WOCBP who are pregnant or breastfeeding

b) Women with a positive pregnancy test at enrolment or prior to administration of study medication.

6. Other Exclusion Criteria

a) Prisoners or participants who are involuntarily incarcerated

b) Participants who are compulsorily detained for treatment of either a psychiatric or physical (e.g., infectious disease) illness

Eligibility criteria for this study have been carefully considered to ensure the safety of the study participants and that the results of the study can be used. It is imperative that participants fully meet all eligibility criteria.

3.3.3 Women of Childbearing Potential

Women of childbearing potential (WOCBP) is defined as any female who has experienced menarche and who has not undergone surgical sterilization (hysterectomy or bilateral oophorectomy) or is not postmenopausal. Menopause is defined clinically as 12 months of amenorrhea in a woman over age 45 in the absence of other biological or physiological causes. In additional, women under the age of 62 must have a documented serum follicle stimulating hormone, (FSH) level > 40mIU/mL.

3.4 Concomitant Treatments

3.4.1 Prohibited and/or Restricted Treatments

The following medications are prohibited during the study:

- Immunosuppressive agents (except to treat a drug-related adverse event)
- Systemic corticosteroids > 10 mg daily prednisone equivalent (except as stated in Section 3.4.4 or to treat a drug-related adverse event).
- Any concurrent antineoplastic therapy (i.e., chemotherapy, hormonal therapy, immunotherapy, radiation therapy except for palliative radiation therapy described in Section 3.4.2.1, or standard or investigational agents for treatment of cancer).

Supportive care for disease-related symptoms may be offered to all participants on the trial.

3.4.2 Other Restrictions and Precautions

3.4.2.1 Palliative Therapy

Palliative (limited-field) radiation therapy and palliative surgical resection are permitted if the following criteria are met:

1. The participant is considered to have progressed at the time of palliative therapy and meets criteria to continue with treatment beyond progression (Section 4.3.7).
2. The case is discussed with the Study Physician. Palliative therapy must be clearly documented as such in the study record.

3.4.3 Surgical Resection Following initial Response

Investigators may choose to resect lesions in patients with oligometastatic disease and render the patient free of macroscopic disease. Participants enrolled in this study may have lesions surgically resected only following consultation with the Medical Monitor and following the Week 18 re-staging assessments. If tumour shrinkage of the solitary lesion is noted on the re-staging assessment (e.g., Week 18), it is highly encouraged that surgical resection be delayed until subsequent scans fail to demonstrate further shrinkage. Patients with a PR who go on to have surgical resection of remaining disease will be considered a PR. Tumour tissue of any resected solitary lesion should be submitted for biomarker analysis. Detailed instructions of the obtaining, processing, labelling, handling, storage and shipment of these specimens will be provided in a separate Procedure Manual at the time of study initiation.

3.4.4 Permitted Therapy

Participants are permitted to use topical, ocular, intra-articular, intranasal, and inhalational corticosteroids (with minimal systemic absorption). Physiologic replacement doses of systemic corticosteroids are permitted, even if > 10 mg/day prednisone equivalents. A brief course of corticosteroids for prophylaxis (e.g., contrast dye allergy) or for treatment of non-autoimmune conditions (e.g., delayed-type hypersensitivity reaction caused by contact allergen) is permitted. Prophylactic administration of 100 mg hydrocortisone (or equivalent) by IV may be allowed in the setting of infusion reactions if deemed appropriate by the PI or medical monitor.

Concomitant medications are recorded at baseline and throughout the treatment phase of the study in the appropriate section of the CRF. All medications (prescriptions or over the counter medications) continued at the start of the study or started during the study and different from the study drug must be documented in the concomitant therapy section of the CRF.

3.5 Discontinuation of Participants from Treatment

Participants MUST discontinue investigational product (and non-investigational product at the discretion of the investigator) for any of the following reasons:

- Participant's request to stop study treatment
- Any clinical adverse event (AE), laboratory abnormality or intercurrent illness which, in the opinion of the investigator, indicates that continued participation in the study is not in the best interest of the participant
- Pregnancy
- Termination of the study by the sponsor.

- Loss of ability to freely provide consent through imprisonment or involuntarily incarceration for treatment of either a psychiatric or physical (e.g., infectious disease) illness
- Additional protocol specified reasons for discontinuation (see Section 4.3.5)

All participants who discontinue investigational product should comply with protocol specified follow-up procedures as outlined in Section 5 - Study Assessment and Procedures. The only exception to this requirement is when a participant withdraws consent for all study procedures including post-treatment study follow-up or loses the ability to consent freely (i.e., is imprisoned or involuntarily incarcerated for the treatment of either a psychiatric or physical illness).

If study treatment is discontinued prior to the participant's completion of the study, the reason for the discontinuation must be documented in the participant's medical records and entered on the appropriate case report form (CRF) page.

3.6 Post Treatment Study Follow up

Participants who discontinue study treatment will continue to be followed for collection of outcome and/or survival follow-up data as required and in line with Section 5 Study Assessments and Procedures until death or the conclusion of the study.

3.6.1 Withdrawal of Consent

Participants who request to discontinue study treatment will remain in the study and must continue to be followed for protocol specified follow-up procedures. The only exception to this is when a participant specifically withdraws consent for any further contact with him/her or persons previously authorized by participant to provide this information. Participants should notify the investigator of the decision to withdraw consent from future follow-up **in writing**, whenever possible. The withdrawal of consent should be explained in detail in the medical records by the investigator, as to whether the withdrawal is from further treatment with study drug only or also from study procedures and/or post treatment study follow-up, and entered on the appropriate CRF page. In the event that vital status (whether the participant is alive or dead) is being measured, publicly available information should be used to determine vital status only as appropriately directed in accordance with local law.

3.6.2 Lost to Follow-Up

All reasonable efforts must be made to locate participants to determine and report their ongoing status. This includes follow-up with persons authorized by the participant as noted above. Lost to follow-up is defined by the inability to reach the participant after a minimum of three documented phone calls, faxes, or emails as well as lack of response by participant to one registered mail letter. All attempts should be documented in the participant's medical records. If it is determined that the participant has died, the site will use permissible local methods to obtain the date and cause of death.

If investigator's use of third-party representative to assist in the follow-up portion of the study has been included in the participant's informed consent, then the investigator may use a Sponsor-retained third-party representative to assist site staff with obtaining participant's contact information or other public vital status data necessary to complete the follow-up portion of the study. If after all attempts, the participant remains lost to follow-up, then the last known

alive date as determined by the investigator should be reported and documented in the participant's medical records.

4 TREATMENTS

Study drugs include both Non-investigational (NIMP) and Investigational Medicinal Products (IMP) and can consist of the following:

- All products, active or placebo, being tested or used as a comparator in a clinical trial.
- Study required premedication, and
- Other drugs administered as part of the study that are critical to claims of efficacy (e.g., backbone therapy, rescue medications)
- Diagnostic agents: (such as glucose for glucose challenge) given as part of the protocol requirements must also be included in the dosing data collection.

4.1 Study Treatments

Table 4.1-1: Product Description: Treatment Period					
**Product Description and Dosage Form**	**Potency**	**Primary Packaging (Volume)/ Label Type**	**Secondary Packaging (Qty) /Label Type**	**Appearance**	**Storage Conditions (per label)**
BMS-936558-01 Solution for Injection[a]	100 mg (10 mg/mL)	10 mL vial/ Open-label	5-10 vials per carton/ Open-label	Clear to opalescent colorless to pale yellow liquid. May contain particles	2 to 8°C. Protect form light and freezing
Ipilimumab Solution for Injection	200 mg (5 mg/mL)	40 mL vial/Open-label	4 vials per carton/Open-label	Clear, colorless to pale yellow liquid. May contain particles	2 to 8°C. Protect from light and freezing.

[a] Nivolumab is labelled as BMS-936558-01 Solution for Injection

Premedications or medications used to treat infusion-related reactions should be sourced by the investigative sites if available and permitted by local regulations.

If stored in a glass front refrigerator, vials should be stored in the carton. Recommended safety measures for preparation and handling of nivolumab and ipilimumab include laboratory coats and gloves.

For additional details on prepared drug storage and use time of nivolumab or ipilimumab under room temperature/light and refrigeration, please refer to the BMS-936558 (nivolumab) and Ipilimuab Investigator Brochure section for “Recommended Storage and Use Conditions”

.

4.1.1 Investigational Product

An investigational product, also known as investigational medicinal product in some regions, is defined a pharmaceutical form of an active substance or placebo being tested or used as a reference in a clinical study, including products already with a marketing authorization but used or assembled (formulated or packaged) differently than the authorized form, or used for an unauthorized indication, or when used to gain further information about the authorized form.

The investigational product should be stored in a secure area according to local regulations. It is the responsibility of the investigator to ensure that investigational product is only dispensed to study participants. The investigational product must be dispensed only from official study sites by authorized personnel according to local regulations.

In this protocol, investigational product(s) is/are: BMS-936558 (nivolumab) and ipilimumab-solution for injection.

4.1.2 Non-investigational Product

Other medications used as support or escape medication for preventative, diagnostic, or therapeutic reasons, as components of the standard of care for a given diagnosis, may be considered as non-investigational products.

In this protocol, non-investigational product(s) is/are: not applicable

4.1.3 Handling and Dispensing

The product storage manager should ensure that the study drug is stored in accordance with the environmental conditions (temperature, light, and humidity) as determined by BMS. If concerns regarding the quality or appearance of the study drug arise, the study drug should not be dispensed and contact BMS immediately.

Investigational product documentation must be maintained that includes all processes required to ensure drug is accurately administered. This includes documentation of drug storage, administration and, as applicable, storage temperatures, reconstitution, and use of required processes (e.g., required diluents, administration sets).

Infusion-related supplies (e.g., IV bags, in-line filters, 0.9% NaCl solution) will not be supplied by the sponsor and should be purchased locally if permitted by local regulations.

For non-investigational product, if marketed product is utilized, it should be stored in accordance with the package insert, summary of product characteristics (SmPC), or similar.

Please refer to the current version of the Investigator Brochure and/or pharmacy reference sheets for complete storage, handling, dispensing, and infusion information for BMS-936558 (nivolumab) and ipilimumab.

<u>Nivolumab (BMS-936558)</u>

Nivolumab (BMS-936558) vials must be stored at a temperature of 2°C to 8°C and should be protected from light and freezing. If stored in a glass front refrigerator, vials should be stored in the carton. Recommended safety measures for preparation and handling of nivolumab include laboratory coats and gloves.

For details on prepared drug storage and use time of nivolumab under room temperature/light and refrigeration, please refer to the BMS-936558 (nivolumab) Investigator Brochure section for “Recommended Storage and Use Conditions” and/or pharmacy reference sheets.

Care must be taken to assure sterility of the prepared solution as the product does not contain any anti-microbial preservative or bacteriostatic agent. No incompatibilities between nivolumab and polyolefin bags have been observed.

Nivolumab or Nivolumab-Placebo is to be administered as a 30-minute IV infusion, using a volumetric pump with a 0.2/0.22 micron in-line filter at the protocol-specified dose. The drug can be diluted with 0.9% normal saline for delivery but the total drug concentration of the solution cannot be below 0.35 mg/ml. It is not to be administered as an IV push or bolus injection. At the end of the infusion, flush the line with a sufficient quantity of normal saline.

Ipilimumab

Ipilimumab injection can be used for IV administration without dilution after transferring to a PVC (polyvinyl chloride), non-PVC/non-DEHP (di-(2-ethylhexyl)phthalate) or glass containers and is stable for 24 hours at 2-8ºC or room temperature/room light (RT/RL). For ipilimumab storage instructions, refer to ipilimumab IB and/or pharmacy reference sheets.

Recommended safety measures for preparation and handling include protective clothing, gloves, and safety cabinets.

The same storage and use conditions recommended for product also apply to the placebo for Ipilimumab Injection.

Ipilimumab is to be administered as a 90-minute IV infusion, using a volumetric pump with a 0.2 to 1.2 micron in-line filter at the protocol-specified dose. The drug can be diluted with 0.9% normal saline or 5% Dextrose Injection to concentrations between 1 mg/mL and 4 mg/mL. It is not to be administered as an IV push or bolus injection. Care must be taken to assure sterility of the prepared solutions, since the drug product does not contain any antimicrobial preservatives or bacteriostatic agents. At the end of the infusion, flush the line with a sufficient quantity of normal saline or 5% dextrose solution.

When both study drugs are to be administered on the same day, separate infusion bags and filters must be used for each infusion. Nivolumab is to be administered first. The nivolumab infusion must be promptly followed by a saline flush to clear the line of nivolumab before starting the ipilimumab infusion.

4.2 Method of Assigning Participant Identification

Every participant that signs the informed consent form must be assigned a participant number. The investigator or designee will register the participant for enrolment by following the enrolment procedures established by the sponsor. The following information is required for enrolment:

- Date that informed consent was obtained
- Date of birth
- Gender at birth

Participants meeting all eligibility criteria will be enrolled

4.3 Selection and Timing of Dose for Each Participant

Dosing schedule is detailed in Table 4.3-1 and Table 4.3-2.

Table 4.3-1: Dosing Schedule for Cycle 1 and Cycle 2

1 Cycle = 6 weeks						
	**Day 1<br>Week 1**	**Day 1<br>Week 2**	**Day 1<br>Week 3**	**Day 1<br>Week 4**	**Day 1<br>Week 5**	**Day 1<br>Week 6**
Nivolumab 3mg/kg + Ipilimumab 1 mg/kg)	3 mg/kg Nivolumab<br><br>1 mg/kg Ipilimumab			3 mg/kg Nivolumab<br><br>1 mg/kg Ipilimumab		

Table 4.3-2: Dosing Schedule Cycle 3 and Beyond

1 Cycle = 6 weeks						
	**Day 1<br>Week 1**	**Day 1<br>Week 2**	**Day 1<br>Week 3**	**Day 1<br>Week 4**	**Day 1<br>Week 5**	**Day 1<br>Week 6**
Nivolumab	3 mg/kg Nivolumab		3 mg/kg Nivolumab		3 mg/kg Nivolumab	
*Under exceptional circumstances- requires prior sponsor approval						
**1 Cycle = 9 weeks**						
	**Day 1<br>Week 1**	**Day 1<br>Week 5**	**Day 1<br>Week 9**			
Nivolumab	480mg	480mg	480mg			

First dose to be administered within 7 days following enrolment. When study drugs (ipilimumab or nivolumab) are administered on the same day, separate infusion bags and filters must be used for each infusion. Nivolumab is to be administered first. The second infusion will always be the ipilimumab and will start no sooner than 30 minutes after completion of the nivolumab or nivo-placebo infusion.

Ipilimumab may be diluted in 0.9% Sodium Chloride Solution or 5% Dextrose solution. Nivolumab or nivolumab-placebo may be diluted in 0.9% Sodium Chloride Solution.

The dosing calculations should be based on the body weight. If the participant's weight on the day of dosing differs by > 10% from the weight used to calculate the dose, the dose must be recalculated. All doses should be rounded up or to the nearest milligram per institutional standard. There will be no dose modifications allowed.

During cycles 1 and 2

- participants may be dosed no less than 12 days during the combination treatment period, therefore between
  - C1W1 and C1W3
  - C1W5 and C2W1
  - C2W1 and C2W3
- participants may be dosed no less than 5 days during the combination treatment period, therefore between
  - C1W3 and C1W4
  - C1W4 and C1W5
  - C2W3 and C2W4
  - C2W4 and C2W5

Starting from cycle 3 (monotherapy treatment period), participants may be dosed no less than 12 days from the previous dose of drug.

Participants may be dosed up to 3 days after the scheduled date if necessary. Subsequent dosing should be based on the actual date of administration of the previous dose of drug.

Treatment compliance will be monitored by drug accountability as well as the participant's medical record and eCRF.

4.3.1 Antiemetic Premedications

Antiemetic premedications should not be routinely administered prior to dosing of drugs. See section 4.3.6 for premedication recommendations following a nivolumab or ipilimumab related infusion reaction.

4.3.2 Dose Delay Criteria

Because of the potential for clinically meaningful nivolumab-related AEs requiring early recognition and prompt intervention, management algorithms have been developed for suspected AEs of selected categories. [see current Investigator Brochure and Appendix for citation examples]

Dose delay criteria apply for all drug-related adverse events (regardless of whether or not the event is attributed to nivolumab, ipilimumab, or both). All study drugs must be delayed until treatment can resume (see Section 4.3.4.)

Nivolumab and ipilimumab administration should be delayed for the following:

- Any Grade ≥ 2 non-skin, drug-related adverse event, with the following exceptions:
  - Grade 2 drug-related fatigue or laboratory abnormalities do not require a treatment delay
- Any Grade 3 skin, drug-related adverse event
- Any Grade 3 drug-related laboratory abnormality, with the following exceptions for asymptomatic amylase or lipase, AST, ALT, or total bilirubin:
  - Grade 3 amylase or lipase abnormalities that are not associated with symptoms or clinical manifestations of pancreatitis do not require a dose delay. It is recommended to consult with the Principal Investigator for Grade 3 amylase or lipase abnormalities.
  - If a participant has a baseline AST, ALT, or total bilirubin that is within normal limits, delay dosing for drug-related Grade ≥ 2 toxicity
  - If a participant has baseline AST, ALT, or total bilirubin within the Grade 1 toxicity range, delay dosing for drug-related Grade ≥ 3 toxicity
- Any adverse event, laboratory abnormality, or intercurrent illness which, in the judgment of the investigator, warrants delaying the dose of study medication.

4.3.2.1 Management Algorithms for Immuno-Oncology Agents

Immuno-oncology (I-O) agents are associated with adverse events that can differ in severity and duration than adverse events caused by other therapeutic classes. Nivolumab and ipilimumab are considered immuno-oncology agents in this protocol. Early recognition and management of adverse events associated with immuno-oncology agents may mitigate severe toxicity. Management algorithms have been developed to assist investigators in assessing and managing the following groups of adverse events:

- Gastrointestinal
- Renal
- Pulmonary
- Hepatic
- Endocrinopathies
- Skin
- Neurological

While the ipilimumab investigator brochure contains safety management algorithms for similar adverse events, <u>the recommendations are to follow the nivolumab algorithms for immune-oncology agents (I-O).</u>

The recommendations are to follow the algorithms in the nivolumab investigator brochure for immune related events; while the ipilimumab investigator brochure contains similar algorithms, the algorithms in the nivolumab brochure have been aligned to accommodate combinations as well as nivolumab monotherapy

Additional details on the safety of nivolumab and ipilimumab, including results from clinical studies, are available in the IB.

For participants expected to require more than 4 weeks of corticosteroids or other immunosuppressants to manage an adverse event, consider recommendations provided in Section 1.2.4.2 Summary of Safety - Adverse Event Management Algorithms.

4.3.3 Dose Modifications

Dose reductions or dose escalations are not permitted.

4.3.4 Criteria to Resume Treatment

Participants may resume treatment with study drug when the drug-related AE(s) resolve to Grade ≤ 1 or baseline value, with the following exceptions:

- Participants may resume treatment in the presence of Grade 2 fatigue
- Participants who have not experienced a Grade 3 drug-related skin AE may resume treatment in the presence of Grade 2 skin toxicity
- Participants with baseline Grade 1 AST/ALT or total bilirubin who require dose delays for reasons other than a 2-grade shift in AST/ALT or total bilirubin may resume treatment in the presence of Grade 2 AST/ALT OR total bilirubin
- Participants with combined Grade 2 AST/ALT AND total bilirubin values meeting discontinuation parameters (Section 4.3.5) should have treatment permanently discontinued
- Drug-related pulmonary toxicity, diarrhea, or colitis, must have resolved to baseline before treatment is resumed
- Drug-related endocrinopathies adequately controlled with only physiologic hormone replacement may resume treatment

If the criteria to resume treatment is met, the participant should restart treatment at the next scheduled time point per protocol. However, if the treatment is delayed past the next scheduled time point per protocol, the next scheduled time point will be delayed until dosing resumes.

If treatment is delayed > 6 weeks, the participant must be permanently discontinued from study therapy, except as specified in Section 4.3.5.

4.3.5 Discontinuation Criteria.

Treatment should be permanently discontinued for the following:

- Any Grade 2 drug-related uveitis or eye pain or blurred vision that does not respond to topical therapy and does not improve to Grade 1 severity within the re-treatment period OR requires systemic treatment
- Any Grade 3 non-skin, drug-related adverse event lasting > 7 days, with the following exceptions for drug-related laboratory abnormalities, uveitis, pneumonitis, bronchospasm, diarrhea, colitis, neurologic toxicity, hypersensitivity reactions, and infusion reactions:
  - Grade 3 drug-related uveitis, pneumonitis, bronchospasm, diarrhea, colitis, neurologic toxicity, hypersensitivity reaction, or infusion reaction of any duration requires discontinuation

  - Grade 3 drug-related laboratory abnormalities do not require treatment discontinuation except:
    - ♦ Grade 3 drug-related thrombocytopenia > 7 days or associated with bleeding requires discontinuation
    - ♦ Any drug-related liver function test (LFT) abnormality that meets the following criteria require discontinuation:
      - AST or ALT > 8 x ULN
      - Total bilirubin > 5 x ULN
      - Concurrent AST or ALT > 3 x ULN and total bilirubin > 2 x ULN
- Any Grade 4 drug-related adverse event or laboratory abnormality, except for the following events which do not require discontinuation:
  - Grade 4 amylase or lipase abnormalities that are not associated with symptoms or clinical manifestations of pancreatitis. It is recommended to consult with the BMS Medical Monitor for Grade 4 amylase or lipase abnormalities.
  - Isolated Grade 4 electrolyte imbalances/abnormalities that are not associated with clinical sequelae and are corrected with supplementation/appropriate management within 72 hours of their onset
- Any dosing interruption lasting > 6 weeks with the following exceptions:
  - Dosing interruptions to allow for prolonged steroid tapers to manage drug-related adverse events are allowed. Prior to re-initiating treatment in a participant with a dosing interruption lasting > 6 weeks, the PI must be consulted. Tumour assessments should continue as per protocol even if dosing is interrupted.
  - Dosing interruptions > 6 weeks that occur for non-drug-related reasons may be allowed if approved by the BMS medical monitor. Prior to re-initiating treatment in a participant with a dosing interruption lasting > 6 weeks, the PI must be consulted. Tumour assessments should continue as per protocol even if dosing is interrupted.
- Any adverse event, laboratory abnormality, or intercurrent illness which, in the judgment of the Investigator, presents a substantial clinical risk to the participant with continued nivolumab or ipilimumab dosing.

4.3.6 Treatment of Nivolumab or Ipilimumab Related Infusion Reactions

Since nivolumab and ipilimumab contains only human immunoglobulin protein sequences, it is unlikely to be immunogenic and induce infusion or hypersensitivity reactions. However, if such a reaction were to occur, it might manifest with fever, chills, rigors, headache, rash, pruritus, arthralgias, hypo- or hypertension, bronchospasm, or other symptoms. All Grade 3 or 4 infusion reactions should be reported within 24 hours to the BMS Medical Monitor and reported as an SAE if criteria are met. Infusion reactions should be graded according to NCI CTCAE (version 4.0) guidelines.

Treatment recommendations are provided below and may be modified based on local treatment standards and guidelines as appropriate:

For Grade 1 symptoms: (Mild reaction; infusion interruption not indicated; intervention not indicated)

Remain at bedside and monitor participant until recovery from symptoms. The following prophylactic premedications are recommended for future infusions: diphenhydramine 50 mg

(or equivalent) and/or paracetamol 325 to 1000 mg at least 30 minutes before additional nivolumab administrations.

For Grade 2 symptoms: (Moderate reaction requires therapy or infusion interruption but responds promptly to symptomatic treatment [e.g., antihistamines, non-steroidal anti-inflammatory drugs, narcotics, corticosteroids, bronchodilators, IV fluids]; prophylactic medications indicated for ≤ 24 hours).

Stop the nivolumab or ipilimumab infusion, begin an IV infusion of normal saline, and treat the participant with diphenhydramine 50 mg IV (or equivalent) and/or paracetamol 325 to 1000 mg; remain at bedside and monitor participant until resolution of symptoms. Corticosteroid or bronchodilator therapy may also be administered as appropriate. If the infusion is interrupted, then restart the infusion at 50% of the original infusion rate when symptoms resolve; if no further complications ensue after 30 minutes, the rate may be increased to 100% of the original infusion rate. Monitor participant closely. If symptoms recur then no further nivolumab or ipilimumab will be administered at that visit. Administer diphenhydramine 50 mg IV, and remain at bedside and monitor the participant until resolution of symptoms. The amount of study drug infused must be recorded on the electronic case report form (eCRF). The following prophylactic premedications are recommended for future infusions: diphenhydramine 50 mg (or equivalent) and/or paracetamol 325 to 1000 mg should be administered at least 30 minutes before additional nivolumab or ipilimumab administrations. If necessary, corticosteroids (recommended dose: up to 25 mg of IV hydrocortisone or equivalent) may be used.

For Grade 3 or Grade 4 symptoms: (Severe reaction, Grade 3: prolonged [i.e., not rapidly responsive to symptomatic medication and/or brief interruption of infusion]; recurrence of symptoms following initial improvement; hospitalization indicated for other clinical sequelae [e.g., renal impairment, pulmonary infiltrates]). Grade 4: (life-threatening; pressor or ventilatory support indicated).

Immediately discontinue infusion of nivolumab or ipilimumab. Begin an IV infusion of normal saline, and treat the participant as follows. Recommend bronchodilators, epinephrine 0.2 to 1 mg of a 1:1,000 solution for subcutaneous administration or 0.1 to 0.25 mg of a 1:10,000 solution injected slowly for IV administration, and/or diphenhydramine 50 mg IV with methylprednisolone 100 mg IV (or equivalent), as needed. Participant should be monitored until the investigator is comfortable that the symptoms will not recur. Nivolumab or ipilimumab will be permanently discontinued. Investigators should follow their institutional guidelines for the treatment of anaphylaxis. Remain at bedside and monitor participant until recovery from symptoms. In the case of late-occurring hypersensitivity symptoms (e.g., appearance of a localized or generalized pruritus within 1 week after treatment), symptomatic treatment may be given (e.g., oral antihistamine, or corticosteroids).

4.3.7 Treatment Beyond Disease Progression

As described in Section 1.3 accumulating evidence indicates a minority of participants treated with immunotherapy may derive clinical benefit despite initial evidence of PD (33).

Participants (from Cohort A, B, or C, and the expansion cohort) may be permitted to continue treatment beyond initial RECIST 1.1 defined PD as long as they meet the following criteria:

- Investigator-assessed clinical benefit
  and
- Participant is tolerating study drug.

The assessment of clinical benefit should take into account whether the participant is clinically deteriorating and unlikely to receive further benefit from continued treatment.

In order to make informed subsequent treatment decisions, an optional tumour biopsy at time of progression can be considered when assessing whether to treat beyond progression. As discussed in section 5.5 the optional tumour biopsy may also be utilized to investigate potential mechanisms of resistance to immunotherapeutic agents and the impact of treatment on relevant melanoma biomarkers.

Detailed instructions of the labelling, handling, storage and shipment of these specimens will be provided in a separate procedure manual.

All decisions to continue treatment beyond initial progression must be discussed with the Principal Investigator and documented in the study records

Participants should discontinue study therapy upon further evidence of further progression, defined as an additional 10% or greater increase in tumour burden volume from time of initial progression (including all target lesions and new measurable lesions).

New lesions are considered measurable at the time of initial progression if the longest diameter is at least 10 mm (except for pathological lymph nodes, which must have a short axis of at least 15 mm). Any new lesion considered non-measurable at the time of initial progression may become measurable and therefore included in the tumour burden measurement if the longest diameter increases to at least 10 mm (except for pathological lymph nodes, which must have an increase in short axis to at least 15 mm).

For statistical analyses that include the investigator-assessed progression date, participants who continue treatment beyond initial investigator-assessed, RECIST 1.1-defined progression will be considered to have investigator-assessed progressive disease at the time of the initial progression event.

4.4 Treatment Compliance

Treatment compliance will be monitored by drug accountability as well as the participant's medical record and CRF

4.5 Destruction and Return of Study Drug

4.5.1 Destruction of Study Drug

For this study, study drugs (those supplied by BMS or sourced by the investigator) such as partially used study drug containers, vials and syringes may be destroyed on site.

Any unused study drugs can only be destroyed after being inspected and reconciled by the responsible Study Monitor unless study drug containers must be immediately destroyed as required for safety, or to meet local regulations (e.g., cytotoxics or biologics).

On-site destruction is allowed provided the following minimal standards are met:

- On-site disposal practices must not expose humans to risks from the drug.

- On-site disposal practices and procedures are in agreement with applicable laws and regulations, including any special requirements for controlled or hazardous substances.
- Written procedures for on-site disposal are available and followed. The procedures must be filed with the site's SOPs and a copy provided to BMS upon request.
- Records are maintained that allow for traceability of each container, including the date disposed of, quantity disposed, and identification of the person disposing the containers. The method of disposal, i.e., incinerator, licensed sanitary landfill, or licensed waste disposal vendor must be documented.
- Accountability and disposal records are complete, up-to-date, and available for the Monitor to review throughout the clinical trial period.

If conditions for destruction cannot be met the responsible Study Monitor will make arrangements for return of study drug.

It is the investigator's responsibility to arrange for disposal of all empty containers, provided that procedures for proper disposal have been established according to applicable federal, state, local, and institutional guidelines and procedures, and provided that appropriate records of disposal are kept.

4.5.2 Return of Study Drug

All unused and/or partially used study drug that was supplied by BMS should be discussed with BMS. Following approval by BMS, any unused study drug may be destroyed at sites following final pharmacy source data review by the CPM and local SOPs. BMS will be provided with copies of relevant destruction documentation. This information will be retained in the pharmacy site file and archived at the end of study for the nominated period. .

It is the investigator's responsibility to arrange for disposal of all empty containers, provided that procedures for proper disposal have been established according to applicable federal, state, local, and institutional guidelines and procedures, and provided that appropriate records of disposal are kept.

5 STUDY ASSESSMENTS AND PROCEDURES

5.1 Flow Chart/Time and Events Schedule

Table 5.1-1: Screening Assessments

Procedure	Screening Visit	Notes
**Eligibility Assessments**		
Informed Consent	X	
Inclusion/Exclusion Criteria	X	All inclusion/exclusion criteria should be assessed at screening and confirmed prior to registration
Medical History	X	
Tumor Tissue Samples	X	Sufficient tumor tissue from an unresectable or metastatic site (block or minimum of 10 slides; obtained from core biopsy, punch biopsy, excisional biopsy or surgical specimen).
**Safety Assessments**		
Physical Examination	X	
Vital Signs and oxygen saturation	X	Including BP, HR, temperature and oxygen saturation by pulse oximetry. Pulse oximetry at rest and after exertion. Obtain vital signs at the screening visit and within 72 hours prior to first dose
Physical Measurements (including performance status)	X	Height and weight
ECG	X	Within 14 days prior to registration
Assessment of Signs and Symptoms	X	Within 14 days prior to registration
Concomitant Medication Collection	X	Within 14 days prior to registration
Laboratory Tests	X	CBC w/differential, Chemistry panel including: LDH, AST, ALT, ALP, T.Bili, BUN or serum urea level, creatinine, Ca, Mg, Na, K, Cl, Glucose, amylase, lipase, TSH, Free T4, Free T3, Hep B/C(HBV sAG, HCV antibody or HCV RNA), within 14 days prior to registration.
Fecal Sample	X	Within 14 days prior to registration
Pregnancy Test (WOCBP Only)	X	

Table 5.1-1: Screening Assessments

Procedure	Screening Visit	Notes
**<u>Efficacy Assessment</u>**		
Screening/Baseline Tumor Assessments	X	CT/MRI imaging-Chest, Abdomen, Pelvis and Brain and all other known sites of disease within 28 days prior to registration.

Table 5.1-2: On-study Assessments Cycles 1 and 2 Only[a]

Procedure	Cycle 1 and Cycle 2 (Cycle = 6 weeks)						Notes
	Day 1 Week 1	Day 1 Week 2	Day 1 Week 3	Day 1 Week 4	Day 1 Week 5	Day 1 Week 6	
**Safety Assessments**							
Targeted Physical Examination	X			X			To be performed only as clinically indicated within 72 hours prior to dosing.
Vital Signs and Oxygen Saturation	X			X			Including BP, HR, temperature and oxygen saturation by pulse oximetry. Pulse oximetry at rest and after exertion prior to dosing.
Physical Measurements (including performance status)	X			X			Weight and ECOG status within 72 hours prior to dosing
Adverse Events Assessment	Continuously						
Review of Concomitant Medications	X			X			
Laboratory Tests	X			X			Within 72 hrs. prior to dosing to include CBC w/ differential, LFTs, BUN or serum urea level, creatinine, Ca, Mg, Na, K, Cl, LDH, Glucose, amylase, lipase, TSH (with reflexive Free T4 and Free T3)
Pregnancy Test (WOCBP Only)	X			X			Within 24 hours prior to administration of study drug. Serum or Urine
**Exploratory Biomarker Testing**							
Exploratory Serum Biomarkers	X						To be collected pre-dose;
Peripheral Blood RNA	X			Y			To be collected pre-dose; Y= only for Cycle 1

Table 5.1-2: On-study Assessments Cycles 1 and 2 Only[a]

Procedure	Cycle 1 and Cycle 2 (Cycle = 6 weeks)						Notes
	Day 1 Week 1	Day 1 Week 2	Day 1 Week 3	Day 1 Week 4	Day 1 Week 5	Day 1 Week 6	
Peripheral Blood Mononuclear Cells (PBMCs)	X			X			To be collected pre-dose;
Whole Blood Sample (DNA)	Y			Y			EDTA Tubes for DNA. Must be obtained prior to dosing. Y= only for Cycle 1.
Tumour Biopsy (optional)	x			X			Tumour Biopsies will be collected pre-dose from accessible lesions.
**Efficacy Assessments**							
Tumor Assessments						Y	FIRST tumor assessment should first be performed at 12 weeks (± 1 wk) following registration. SUBSEQUENT tumor assessments should occur at week 18 and thereafter every 12 weeks (1 ± wk) until disease progression. CT/MRI imaging; Chest, abdomen, pelvis and all known sites of disease. Use same imaging method as was used at screening/baseline. Y=Cycle 2 only
Administer Study Treatment	X			X			First dose to be administered within 3 days following registration. See section 4.3 Subsequent doses may be administered within 3 days after the scheduled date if necessary.

[a] If a dose is delayed, the procedures scheduled for that same time point should also be delayed to coincide with when that time point's dosing actually occurs.

Table 5.1-3a: On-study Assessments Cycle 3 and 4- Nivolumab 3mg/kg maintence phase

Procedure	Cycle 3 & 4 (Cycle = 6 weeks)						Notes
	Day 1 Week 1	Day 1 Week 2	Day 1 Week 3	Day 1 Week 4	Day 1 Week 5	Day 1 Week 6*	
**<u>Safety Assessments</u>**							
Targeted Physical Examination	X		X		X		To be performed only if clinically indicated within 72 hours prior to dosing
Vital Signs and Oxygen Saturation	X		X		X		Including BP, HR, temperature and oxygen saturation by pulse oximetry. Pulse oximetry at rest and after exertion prior to dosing.
Physical Measurements (including performance status)	X		X		X		Weight and ECOG status within 72 hours prior to dosing.
Adverse Events Assessment	Continuously						
Review of Concomitant Medications	X		X		X		
Laboratory Tests	X				X		Within 72 hrs. prior to re-dosing to include CBC w/ differential, LFTs, BUN or serum urea level, creatinine. Ca, Mg, Na, K, Cl, LDH (or tumour relevant marker[1]), Glucose, amylase, lipase, TSH (with reflexive Free T4 and Free T3)
Pregnancy Test (WOCBP Only)	X				X		Within 24 hours prior to administration of study drug. Serum or Urine

[1] Cancer specific markers or anylates of interest may be tracked during the course of the trial at the discrection of the treating clinician.

Table 5.1-3a: On-study Assessments Cycle 3 and 4- Nivolumab 3mg/kg maintence phase							
**Procedure**	**Cycle 3 & 4 (Cycle = 6 weeks)**						**Notes**
	**Day 1 Week 1**	**Day 1 Week 2**	**Day 1 Week 3**	**Day 1 Week 4**	**Day 1 Week 5**	**Day 1 Week 6***	
**<u>Efficacy Assessments</u>**							
Tumor Assessments						X 12 weekly Cycle 4 onwards	*FIRST tumor assessment should first be performed at 12 weeks (± 1 wk) following enrollment. SUBSEQUENT tumor assessments should occur Cycle 3 week 6 (18 weeks following enrollment) then every 12 weeks (1± wk) until disease progression. CT/MRI: Chest, abdomen, pelvis and all known sites of disease. Use same imaging method as was used at screening/baseline.
**<u>Clinical Drug Supplies</u>**							
Administer Study Treatment	X		X		X		Subsequent doses may be administered within 3 days after the scheduled date if necessary. See section 4.3

If a dose is delayed, the procedures scheduled for that same time point should also be delayed to coincide with when that time point's dosing actually occurs

Table 5.1-3b: On-study Assessments Cycle 3 and 4 – Only for Exceptional Circumstances where Nivolumab is administered Q4W during the maintenance phase

Procedure	Cycle 3 & 4 (Cycle = 9 weeks)			Notes
	Day 1 Week 1	Day 1 Week 5	Day 1 Week 9	
**Safety Assessments**				
Targeted Physical Examination	X	X	X	To be performed only if clinically indicated within 72 hours prior to dosing
Vital Signs and Oxygen Saturation	X	X	X	Including BP, HR, temperature and oxygen saturation by pulse oximetry. Pulse oximetry at rest and after exertion prior to dosing.
Physical Measurements (including performance status)	X	X	X	Weight and ECOG status within 72 hours prior to dosing.
Adverse Events Assessment	Continuously			
Review of Concomitant Medications	X	X	X	
Laboratory Tests	X		X	Within 72 hrs. prior to re-dosing to include CBC w/ differential, LFTs, BUN or serum urea level, creatinine. Ca, Mg, Na, K, Cl, LDH (or tumour relevant marker[2]), Glucose, amylase, lipase, TSH (with reflexive Free T4 and Free T3)
Pregnancy Test (WOCBP Only)	X		X	Within 24 hours prior to administration of study drug. Serum or Urine

[2] Cancer specific markers or anylates of interest may be tracked during the course of the trial at the discrection of the treating clinician.

Table 5.1-3b: On-study Assessments Cycle 3 and 4 – Only for Exceptional Circumstances where Nivolumab is administered Q4W during the maintenance phase

Procedure	Cycle 3 & 4 (Cycle = 9 weeks)			Notes
	Day 1 Week 1	Day 1 Week 5	Day 1 Week 9	
**Efficacy Assessments**				
Tumor Assessments				SUBSEQUENT tumor assessments should occur 18 weeks following enrollment then every 12 weeks (1± wk) until disease progression (when treated with Nivolumab Q4W, this is at C4W2, C7W2 etc).<br>CT/MRI: Chest, abdomen, pelvis and all known sites of disease. Use same imaging method as was used at screening/baseline.
**Clinical Drug Supplies**				
Administer Study Treatment	X	X	X	Subsequent doses may be administered within 3 days after the scheduled date if necessary.<br>See section 4.3

If a dose is delayed, the procedures scheduled for that same time point should also be delayed to coincide with the dosing for that timepoint actually occurs

Table 5.1-3.1a: On-study Assessments Cycle 5 onward

Procedure	Cycle 5 onward (Cycle = 6 weeks)						Notes
	Day 1 Week 1	Day 1 Week 2	Day 1 Week 3	Day 1 Week 4	Day 1 Week 5	Day 1 week 6+	
**Safety Assessments**							
Targeted Physical Examination	X						To be performed if clinically indicated within 72 hours prior to dosing
Vital Signs and Oxygen Saturation	X		X		X		Including BP, HR, temperature and oxygen saturation by pulse oximetry. Pulse oximetry at rest and after exertion prior to dosing.
Physical Measurements (including performance status)	X						To be performed if clinically indicated within 72 hours prior to dosing<br>Weight and ECOG status within 72 hours prior to dosing.
Adverse Events Assessment	Continuously						
Review of Concomitant Medications	X		X		X		
Laboratory Tests	X						Within 72 hrs. prior to re-dosing at start of each cycle to include CBC w/ differential, LFTs, UECs, tumour relevant marker and TSH.
Pregnancy Test (WOCBP Only)	X				X		Within 24 hours prior to administration of study drug. Serum or Urine

Table 5.1-3.1a: On-study Assessments Cycle 5 onward							
**Procedure**	**Cycle 5 onward (Cycle = 6 weeks)**						**Notes**
	**Day 1 Week 1**	**Day 1 Week 2**	**Day 1 Week 3**	**Day 1 Week 4**	**Day 1 Week 5**	**Day 1 week 6+**	
**Efficacy Assessments**							
Tumor Assessments						X	+FIRST tumor assessment should first be performed at 12 weeks (± 1 wk) following enrollment. SUBSEQUENT tumor assessments should occur Cycle 3 week 6 (18 weeks following enrollment) then every 12 weeks (1± wk) until disease progression. CT/MRI: Chest, abdomen, pelvis and all known sites of disease. Use same imaging method as was used at screening/baseline.
**Clinical Drug Supplies**							
Administer Study Treatment	X		X		X		Subsequent doses may be administered within 3 days after the scheduled date if necessary. See section 4.3

Table 5.1-3.1b: On-study Assessments Cycle 5 onward- Only for exceptional cases where Nivolumab is administerd Q4W during the maintenance phase

Procedure	Cycle 5 onward (Cycle = 9 weeks)			Notes
	Day 1 Week 1	Day 1 Week 5	Day 1 Week 9	
**Safety Assessments**				
Targeted Physical Examination	X			To be performed if clinically indicated within 72 hours prior to dosing
Vital Signs and Oxygen Saturation	X	X	X	Including BP, HR, temperature and oxygen saturation by pulse oximetry. Pulse oximetry at rest and after exertion prior to dosing.
Physical Measurements (including performance status)	X			To be performed if clinically indicated within 72 hours prior to dosing<br>Weight and ECOG status within 72 hours prior to dosing.
Adverse Events Assessment	Continuously			
Review of Concomitant Medications	X	X	X	
Laboratory Tests	X			Within 72 hrs. prior to re-dosing at start of each cycle to include CBC w/ differential, LFTs, UECs, tumour relevant marker and TSH.
Pregnancy Test (WOCBP Only)	X		X	Within 24 hours prior to administration of study drug. Serum or Urine
**Efficacy Assessments**				
Tumor Assessments				SUBSEQUENT tumor assessments should occur 18 weeks following enrollment then every 12 weeks (1± wk) until disease progression (when treated with Nivolumab Q4W, this is at C4W2, C7W2 etc).<br>CT/MRI: Chest, abdomen, pelvis and all known sites of disease. Use same imaging method as was used at screening/baseline.
**Clinical Drug Supplies**				
Administer Study Treatment	X	X	X	Subsequent doses may be administered within 3 days after the scheduled date if necessary. See section 4.3

**Table 5.1-4: Follow-up Assessments - *All Participants***			
**Procedure**	**Follow-Up[a], Visits 1 and 2**	**Survival[b], Follow-up Visits**	**Notes**
**Safety Assessments**			
Targeted Physical Examination	X		To assess for potential late emergent study drug related issues
Adverse Events Assessment	X	X	
Laboratory Tests	X		On site/local CBC w/differential, LFTs, BUN, creatinine and TSH for X01, repeat at X02 if study drug related toxicity persists.
Pregnancy Test	X		Serum or urine
Review of Concomitant Medication	X		
**Survival Status**			
Participant Status	X	X	Every 3 months, may be accomplished by visit or phone contact, to include subsequent anti-cancer therapy
**Efficacy Assessments**			
Tumor Assessments	X		Only for participants **without progression** on study therapy. FIRST tumor assessment should first be performed at 12 weeks (± 1 wk) following registration SUBSEQUENT tumor assessments should occur every 12 wks. (± 1 wk) until disease progression CT/MRI: Chest, abdomen, pelvis and all known sites of disease. Use same imaging method as was used at screening/baseline.

[a] Follow-up visit 1 (FU1) = 30 days from the last dose +/- 7 days or coincide with the date of discontinuation (+/- 7 days) if date of discontinuation is greater than 37 days after last dose, Follow-up visit 2 (FU2) = 84 days (+/- 7 days) from follow-up visit 1

[b] Survival visits = every 3 months from FU2 +/- 7 days. PI may request that survival data be collected on all enrolled participants outside of the protocol defined window

5.2 Study Materials

- NCI CTCAE version 4.0
- Nivolumab Investigator Brochure
- Ipilimumab Investigator Brochure
- Pharmacy Binder
- Laboratory manuals for collection and handling of blood (for biomarker assessment), tissue specimens and stool samples.
- Manual for entry of local laboratory data
- Pregnancy Surveillance Forms
- RECIST 1.1 pocket guide

5.3 Safety Assessments

At baseline, a medical history will be obtained to capture relevant underlying conditions. The baseline examinations should include weight, height, ECOG Performance Status, BP, HR, temperature and oxygen saturation by pulse oximetry at rest and after exertion and should be performed as noted in Table 5.1-1 Notes Baseline signs and symptoms are those that are assessed within 14 days prior to registration. Concomitant medications will be collected from within 14 days prior to registration through the study treatment period (see Section 5.1).

Baseline local laboratory assessments should be done within 14 days prior to registration to include: CBC w/differential, LFTs (ALT, AST, total bilirubin, alkaline phosphatase), BUN or serum urea level, creatinine, Ca, Mg, Na, K, Cl, LDH, glucose, amylase, lipase, TSH, Free T4, Free T3, and Hep B and C testing (HBV sAg, HCV Ab or HCV RNA) (see Table 5.1-1). Pregnancy testing for WOCBP (done locally) must be performed within 24 hours prior to the initial administration of study drug at baseline and then within 24 hours of dosing at Week 1 and Week 4 of cycle 1 and 2, and Week 1 and Week 5 starting from cycle 3 and at the safety follow up visits. After discontinuation from nivolumab these should be repeated at approximately 30 days and approximately 70 days [or more frequently if required by local standard]. If participants are receiving Nivolumab 480mg Q4W under exceptional circumstances, then pregnancy testing should be performed at Weeks 1 and 8 starting from cycle 3, at the safety follow up visits and after discontinuation from Nivolumab as specified above.

Participants will be evaluated for safety if they have received any study drug. Toxicity assessments will be continuous during the treatment phase. During the safety follow-up phase Table 5.1-4, toxicity assessments should be done in person. Once participants reach the survival follow-up phase either in person or documented telephone calls to assess the participant's status are acceptable.

Adverse events and laboratory values will be graded according to the NCI-CTCAE version 4.0.

On-study weight and ECOG Performance status and vital signs should be assessed prior to administration of study drug. Vital signs should also be taken as per institutional standard of care prior to, during and after dosing. Oxygen saturation by pulse oximetry at rest and after exertion should be assessed at each on-study visit prior to dosing. The start and stop time of the nivolumab infusion should be documented. Physical examinations are to be performed as

clinically indicated. If there are any new or worsening clinically significant changes since the last exam, report changes on the appropriate non-serious or serious adverse event page.

On study local laboratory assessments should be done within 72 hours of dosing to include; CBC w/differential, LFTs (ALT, AST, total bilirubin, alkaline phosphatase), BUN or serum urea level, creatinine, Ca, Mg, Na, K, Cl, LDH, glucose, amylase, lipase, TSH with reflexive Free T4, Free T3 on Day 1 of Weeks 1 and 4 for Cycles 1 and 2 and on Day 1 of Weeks 1 and 5 starting from Cycle 3 (or on Day 1 of Weeks 1 and 8 if the participant is receiving Nivolumab 480mg Q4W under exceptional circumstances.. Additional measures including non-study required laboratory tests should be performed as clinically indicated or to comply with local regulations. Laboratory toxicities (e.g., suspected drug inducted liver enzyme elevations) will be monitored during the follow-up phase via on site/local labs until all study drug related toxicities resolve, return to baseline or are deemed irreversible.

Oxygen saturation by pulse oximetry should be obtained prior to each dosing and at any time a participant has any new or worsening respiratory symptoms. A reading at rest and on exertion should be obtained at each time point. The extent of the exertion should be based on the judgment of the investigator, but should remain consistent for each individual participant throughout the study. If the patient's participant's status changes, the investigator can alter the extent of exertion based on their medical judgment. If a participant shows changes on pulse oximetry or other pulmonary related signs (hypoxia, fever) or symptoms (e.g., dyspnea, cough, and fever) consistent with possible pulmonary adverse events, the patient participant should be immediately evaluated to rule out pulmonary toxicity. An algorithm for the management of suspected pulmonary toxicity can be found in the nivolumab Investigator's Brochure.

Some of the previously referred to assessments may not be captured as data in the eCRF. They are intended to be used as safety monitoring by the treating physician. Additional testing or assessments may be performed as clinically necessary or where required by institutional or local regulations.

5.3.1 Imaging Assessment for the Study

Any incidental findings of potential clinical relevance that are not directly associated with the objectives of the protocol should be evaluated and handled by the Study Investigator as per standard medical/clinical judgment.

5.4 Efficacy Assessments

Study evaluations will take place in accordance with the flow charts in Section 5.1. Baseline assessments should be performed within 28 days prior to enrolment utilizing CT or MRI. In addition to chest, abdomen, pelvis, and brain, all known sites of disease should be assessed at baseline. Subsequent assessments should include chest, abdomen, and pelvis, and all known sites of disease and should use the same imaging method as was used at baseline. Participants will be evaluated for tumour response beginning 12 weeks (± 1 week) from enrolment, at 18 weeks (± 1 week) and continuing every 12 weeks (± 1 week) thereafter, until disease progression is documented or treatment is discontinued (whichever occurs later). Tumour assessments for ongoing study treatment decisions will be completed by the investigator using RECIST (Response Evaluation Criteria in Solid Tumors) 1.1 criteria. Radiographic images will be collected for independent radiological review committee tumour assessment.

5.4.1 Primary Efficacy Assessment

Clinical benefit rate for whole population (CR+PR+SD>3months)

5.4.2 Secondary Efficacy Assessment

To identify whether a common predictive biomarker or immune signature can be identified in responding patients that can occur irrespective of tumor type.

5.4.3 Assessment of Overall Tumor Burden and Measurable Disease

To serially evaluate tumour response to therapy, it is necessary to estimate the overall tumour burden at baseline and use this as a comparator for subsequent measurements. Measurable disease is defined by the presence of at least one measurable tumour lesion. When CT scans have slice thickness greater than 5 mm, the minimum size for a measurable lesion should be twice the slice thickness.

At baseline, tumour lesions/lymph nodes will be categorized as measurable or non-measurable as follows in Sections 5.4.3.1, 5.4.3.2, and 5.4.3.3

5.4.3.1 Measurable Lesions

Measurable lesions must be accurately measured in at least one dimension (longest diameter in the plane of the measurement to be recorded) with a minimum size of:

- 10 mm by CT/MRI scan - (CT/MRI scan slice thickness no greater than 5 mm)
- 10 mm calliper measurement by clinical exam (lesions which cannot be accurately measured with callipers should be recorded as non-measurable)
- 20 mm by chest x-ray.

Malignant lymph nodes: To be considered pathologically enlarged and measurable, a lymph node must be ≥ 15 mm in short axis when assessed by CT scan (CT scan slice thickness recommended to be no greater than 5 mm). At baseline and in follow up, only the short axis will be measured and followed.

5.4.3.2 Non-Measurable Lesions

- All other lesions, including small lesions (longest diameter < 10 mm or pathological lymph nodes with ≥ 10 to < 15 mm short axis), as well as truly non-measurable lesions.
- Lesions considered truly non-measurable include: leptomeningeal disease, ascites, pleural or pericardial effusion, inflammatory breast disease, lymphangitic involvement of skin or lung, abdominal masses/abdominal organomegaly identified by physical exam that is not measurable by reproducible imaging techniques.

5.4.3.3 Special Considerations Regarding Lesion Measurability

Bone Lesions

- Bone scan, PET scan, or plain films are not considered adequate imaging techniques to measure bone lesions. However, these techniques can be used to confirm the presence or disappearance of bone lesions.
- Lytic bone lesions or mixed lytic-blastic lesions, with identifiable soft tissue components, that can be evaluated by cross sectional imaging techniques such as CT or MRI can be considered as measurable lesions if the soft tissue component meets the definition of measurability described above.

- Blastic bone lesions are non-measurable.

Cystic Lesions

Lesions that meet the criteria for radiographically defined simple cysts should not be considered as malignant lesions (neither measurable nor non-measurable) since they are by definition, simple cysts.

'Cystic lesions' thought to represent cystic metastases can be considered as measurable lesions, if they meet the definition of measurability described above. However, if noncystic lesions are present in the same participant, these are preferred for selection as target lesions.

Non-measurable Lesions

Tumour lesions situated in a previously irradiated area, or in an area subjected to locoregional therapy, are usually not considered measurable unless there has been demonstrated progression in the lesion.

5.4.4 Specifications by Method of Measurement

5.4.4.1 Measurement of Lesions

All measurements should be recorded in metric notation (mm). All baseline evaluations should be performed as close as possible to the treatment start and never more than 28 days before the beginning of treatment.

5.4.4.2 Method of Assessment

The same method of assessment and the same technique should be used to characterize each identified and reported lesion at baseline and during follow-up. Imaging based evaluation should always be done rather than clinical examination unless the lesion(s) being followed cannot be imaged but are assessable by clinical exam.

5.4.4.3 CT/MRI Scan

CT/MRI is the best currently available and reproducible method to measure lesions selected for response assessment. Measurability of lesions on CT/MRI scan is based on the assumption that CT/MRI slice thickness is 5 mm or less. When CT scans have slice thickness greater than 5 mm, the minimum size for a measurable lesion should be twice the slice thickness.

5.4.4.4 Chest X-Ray

Chest CT is preferred over chest x-ray, particularly when progression is an important endpoint, since CT is more sensitive than x-ray, particularly in identifying new lesions. However, lesions on chest x-ray may be considered measurable if they are clearly defined and surrounded by aerated lung.

5.4.4.5 Clinical Lesions

Clinical lesions will only be considered measurable when they are superficial and ≥ 10 mm diameter as assessed using callipers. For the case of skin lesions, documentation by colour photography including a ruler to estimate the size of the lesion is suggested. As previously noted, when lesions can be evaluated both by clinical exam and imaging, imaging evaluation should be undertaken since it is more objective and may also be reviewed at the end of the study.

5.4.4.6 Ultrasound

Ultrasound is ***not*** useful in the assessment of lesion size and should not be used as a method of measurement. If new lesions are identified by ultrasound in the course of the study, confirmation by CT or MRI is advised.

5.4.4.7 Endoscopy, Laparoscopy

The utilization of these techniques for objective tumour evaluation is ***not*** advised.

5.4.4.8 Tumor Markers

Tumour markers such as, but not limited to, LDH may be used for clinical management, but will not be included in the assessment of BOR.

5.4.5 Baseline Documentation of "Target" and "Non-target Lesions"

5.4.5.1 Target Lesions

When more than one measurable lesion is present at baseline all lesions up to a maximum of five lesions total (and a maximum of two lesions per organ) representative of all involved organs should be identified as target lesions and will be recorded and measured at baseline.

Target lesions should be selected on the basis of their size (lesions with the longest diameter), be representative of all involved organs, and should lend themselves to reproducible repeated measurements.

A sum of the diameters (longest for non-nodal lesions, short axis for nodal lesions) for all target lesions will be calculated and reported as the baseline sum diameters. If lymph nodes are to be included in the sum, then as noted below, only the short axis is added into the sum. The baseline sum diameters will be used as reference to further characterize any objective tumour regression in the measurable dimension of the disease.

5.4.5.2 Lymph Nodes

Lymph nodes merit special mention since they are normal anatomical structures which may be visible by imaging even if not involved by tumour. Pathological nodes which are defined as measurable and may be identified as target lesions must meet the criterion of a short axis of ≥ 15 mm by CT scan. Only the short axis of these nodes will contribute to the baseline sum. Nodes that have a short axis < 10 mm are considered non-pathological and should not be recorded or followed.

5.4.5.3 Non-target Lesions

All other lesions (or sites of disease) including pathological lymph nodes should be identified as non-target lesions and should also be recorded at baseline. Measurements are not required and these lesions should be followed as 'present', 'absent', or in rare cases 'unequivocal progression'. In addition, it is possible to record multiple non-target lesions involving the same organ as a single item on the case report form (e.g., 'multiple enlarged pelvic lymph nodes' or 'multiple liver metastases').

5.4.6 Tumor Response Evaluation

Evaluation of Target Lesions

Complete Response (CR): Disappearance of all target lesions. Any pathological lymph nodes (whether target or non-target) must have reduction in short axis to < 10 mm.

Partial Response (PR): At least a 30% decrease in the sum of diameters of target lesions, taking as reference the baseline sum diameters.

Progressive Disease (PD): At least a 20% increase in the sum of diameters of target lesions, taking as reference the smallest sum on study (this includes the baseline sum if that is the smallest on study). In addition to the relative increase of 20%, the sum must also demonstrate an absolute increase of at least 5 mm. (Note: the appearance of one or more new lesions is also considered progression).

Stable Disease (SD): Neither sufficient shrinkage to qualify for PR, nor sufficient increase to qualify for PD, taking as reference the smallest sum diameters while on study.

5.4.6.1 Target Lesions that Become "Too Small to Measure"

All lesions (nodal and non-nodal) recorded at baseline should have their actual measurements recorded at each subsequent evaluation, even when very small (e.g., 2 mm). If the radiologist is able to provide an actual measurement, that should be recorded, even if it is below 5 mm.

5.4.6.2 Target Lesions that Split or Coalesce on Treatment

When non-nodal lesions 'fragment', the longest diameters of the fragmented portions should be added together to calculate the target lesion sum.

As lesions coalesce, a plane between them maybe maintained that would aid in obtaining maximal diameter measurements of each individual lesion. If the lesions have truly coalesced such that they are no longer separable, the vector of the longest diameter in this instance should be the maximal longest diameter for the 'coalesced lesion'.

5.4.6.3 Evaluation of Non-target Lesions

While some non-target lesions may actually be measurable, they need not be measured and instead should be assessed only qualitatively at the time points specified in the protocol.

Complete Response (CR): Disappearance of all non-target lesions. All lymph nodes must be non-pathological in size (< 10 mm short axis).

Non-CR/Non-PD: Persistence of one or more non-target lesion(s) above the normal limits.

Progressive Disease (PD): Unequivocal progression of existing non-target lesions (Note: the appearance of one or more new lesions is also considered progression).

5.4.6.4 Unequivocal Progression in Non-target Disease

To achieve 'unequivocal progression' on the basis of the non-target disease, there must be an overall level of substantial worsening in non-target disease such that, even in presence of SD or PR in target disease, the overall tumour burden has increased sufficiently to merit discontinuation of therapy.

A modest 'increase' in the size of one or more non-target lesions is usually not sufficient to qualify for unequivocal progression status.

5.4.7 New Lesions

The appearance of new malignant lesions denotes disease progression. The finding of a new lesion should be unequivocal: i.e., not attributable to differences in scanning technique, change in imaging modality or findings thought to represent something other than tumour (for example, some 'new' bone lesions may be simply healing or flare of pre-existing lesions. This is particularly important when the participant's baseline lesions show partial or complete response. For example, necrosis of a liver lesion may be reported on a CT scan reported as a 'new' cystic lesion, which it is not.

A lesion identified on a follow-up study in an anatomical location that was not scanned at baseline is considered a new lesion and will indicate disease progression. An example of this is the participant who has visceral disease at baseline and while on study has a CT or MRI brain scan ordered which reveals metastases. The participant's brain metastases are considered to be evidence of PD even if he/she did not have brain imaging at baseline.

If a new lesion is equivocal, for example because of its small size, continued therapy and follow-up evaluation will clarify if it represents truly new disease. If repeat scans confirm there is definitely a new lesion, then progression should be declared using the date of the initial scan.

5.4.8 Response Criteria (RECIST 1.1)

For participants who have measurable disease at baseline, Table 5.4.8-1 provides a summary of the overall response status calculation at each time point.

Table 5.4.8-1: Time Point Response - Participants With Target (± Non-target) Disease

Target Lesions	Non-Target Lesions	New Lesions	Overall Response
CR	CR	No	CR
CR	Non-CR/non-PD	No	PR
CR	Not evaluated	No	PR
PR	Non-PD or not all evaluated	No	PR
SD	Non-PD or not all evaluated	No	SD
Not all evaluated	Non-PD	No	NE
PD	Any	Yes or No	PD
Any	PD	Yes or No	PD
Any	Any	Yes	PD

CR = Complete Response, PR = Partial Response, SD = Stable Disease, PD = Progressive Disease, NE = Not Evaluable

5.4.8.1 Missing Assessments and Not Evaluable Designation

When no imaging/measurement is done at all at a particular time point, the participant is not evaluable (NE) at that time point. If only a subset of lesion measurements are made at an assessment, the case is also considered NE at that time point, unless a convincing argument can be made that the contribution of the individual missing lesion(s) would not have changed the assigned time-point response.

5.4.8.2 Confirmation of Scans

Verification of Response: Confirmation of response is not required since it will not add value to the interpretation of study results per RECIST 1.1.

Verification of Progression: Progression of disease should be verified in cases where progression is equivocal. If repeat scans confirm PD, then progression should be declared using the date of the initial scan. If repeat scans do not confirm PD, then the participant is considered not to have progressive disease per RECIST 1.1.

5.4.9 Best Overall Response

The best overall response is determined once all the data for the participant is known. It is defined as the best response designation, as determined by the investigator, recorded between the date of registration and the date of objectively documented progression per RECIST1.1 or the date of subsequent therapy, whichever occurs first. For participants without documented progression or subsequent therapy, all available response designations will contribute to the BOR assessment. The participant's best overall response assignment will depend on the findings of both target and non-target disease and will also take into consideration the appearance of new lesions.

For purposes of this study, the minimum scan time from baseline for determination of SD will be 9 weeks.

5.4.10 Duration of Objective Response

The duration of objective response is measured from the time measurement criteria are first met for CR/PR (whichever is first recorded) until the first date that progressive disease is objectively documented (taking as reference for progressive disease the smallest measurements recorded on study).

Blood samples should be drawn from a site other than the infusion site (i.e., contralateral arm) on days of infusion. All samples collected pre-dose should be taken just prior to the administration and end-of-infusion (EOI) samples should be taken as close to EOI as possible (preferably within 2 minutes prior to EOI) from the contralateral arm (i.e., the arm not used for the infusion). If the infusion was interrupted, the interruption details will also be documented on the CRF. Blood samples will be processed to collect serum and stored preferably at -70°C (samples may be stored at -20°C up to 2 months).

5.5 Biomarker Assessments

A variety of factors that could potentially predict clinical response to nivolumab in combination with ipilimumab will be investigated in peripheral blood and in tumour specimens taken from all participants prior to treatment and as outlined in Section 5.1. Data from these investigations will be evaluated for associations with response, survival (OS, PFS) and/or safety (adverse

event) data. In addition, analyses of markers between the three cohorts will provide the necessary data to identify and validate biomarkers with predictive vs. prognostic value. All samples collected may also be used for future exploratory analyses (unless restricted by local requirements) to assess biomarkers associated with immunotherapy treatment. The patient is able to opt out of future use of their samples by selecting the future use of sample for cancer research opt out option tick box on the patient and information consent form. Complete instructions on the collection, processing, handling and shipment of all samples described herein will be provided in a separate procedure manual.

5.5.1 Tumor Tissue Specimens

Pre-treatment tumour tissue specimens in the form of a paraffin embedded block or a minimum of 10 unstained slides will be submitted for central immunohistochemistry (IHC) assessment prior to enrolment. These biopsy samples should be excisional, incisional, punch or core needle. Fine needle aspirates or other cytology specimens are insufficient for downstream biomarker analyses. Biomarkers in stained tissue sections will be assessed by a pathologist and scored as PD-L1 positive if membrane staining is observed in ≥ 5% tumour cells among a minimum of a hundred (100) evaluable tumour cells. Samples with < 5% tumour cell membrane staining in a minimum of a hundred (100) evaluable tumour cells will be scored as PD-L1 negative and samples where membrane staining is obscured by high cytoplasmic staining or melanin content, but contain the minimum number of evaluable tumour cells will be deemed PD-L1 indeterminate. Evaluation of other biomarkers will also be undertaken according to local protocols.

These tumour samples, as well as any solitary lesions that may have been surgically resected from participants following an initial response (as described in section 3.4.3), accessible lesions amenable to on-study punch or core needle biopsy (optional) or biopsy samples collected upon progression may also be assessed for the expression of other immune or melanoma related genes, RNAs and/or proteins, as well as, the presence of immune cell populations using a variety of methodologies inclusive of, but not limited to immunohistochemistry (IHC), qRT-PCR, genetic mutation detection and fluorescent in-situ hybridization (FISH). Various molecular markers with potential predictive value for the treatment of melanoma with nivolumab, ipilimumab and other immunotherapies are currently under investigation and may be assessed in this study. These tumour tissue biomarkers include, but are not limited to PD-1, PD-L2, tumour infiltrating lymphocytes (TILs) or subpopulations of TILs and a Th1 immune mRNA expression signature. In addition, other methods of measuring tumour immune marker expression may also be assessed. Tissue from the resected solitary lesions may be assessed for residual tumour cells and for markers expected to accompany tumour shrinkage in this study, including, but not limited to TILs and subsets thereof.

Optional biopsy samples collected upon progression may be used for the assessment of markers implicated in resistance to immunotherapeutic agents, including but not limited to other T cell checkpoint receptors and ligands (e.g., Lag-3, Tim-3) and intratumoral immune cell subsets, including but not limited to, T regulatory cells and myeloid derived suppressor cells. These samples may also be used to investigate the effect of the combination treatment on the expression of potentially relevant predictive and/or prognostic biomarkers, including, but not limited to tumour-relevant mutations and PD-L1.

5.5.2 Exploratory Serum Biomarkers

Blood samples for exploratory serum biomarker analyses will be drawn at the time points indicated in Section 5.1. Blood samples will be processed to collect serum and then put in frozen storage for future use. Samples may be assessed by ELISA, seromics and/or other relevant multiplex-based protein assay methods for immune or melanoma-related factors that will predict for nivolumab or ipilimumab benefit or correlate with nivolumab or ipilimumab efficacy. Numerous potential serum-based biomarkers are currently under investigation for their potential to predict or correlate with efficacy to nivolumab, ipilimumab or other

immunotherapy, including but not limited to levels of soluble PD-L1, anti-tumour antibodies, cytokines, chemokines, inflammatory factors and microRNAs (such as, but not limited to, miR-513 and miR19b).

5.5.3 Peripheral Blood Mononuclear Cells (PBMCs)

Peripheral blood samples will be taken prior to initiation of study therapy and at designated time points on-treatment (see Section 5.1 for additional details on the blood sample collection schedule) for PBMC preparation. Samples must be shipped within 48 hours to the-designated laboratory (Cancer ImmunoBiology Laboratory, Olivia Newton John Cancer Wellness & Research centre, Studley Road, Heidelberg, VIC 3084) for processing.

These PBMC samples may be used for immunophenotyping or characterization of the immune cell subsets in the periphery, including, but not limited to, T cells, B cells, NK cells, or subpopulations of the aforementioned immune cell types. These samples may also be used to assess immune cell function or antigen specific T cell proliferation or activation pending emerging information from other nivolumab or ipilimumab studies.

5.5.4 Peripheral Blood RNA

While immunophenotyping of peripheral blood will provide valuable information on the modulation of the composition of immune cells in the periphery, gene expression analyses of RNA derived from whole blood may provide information on the broad effects of nivolumab and ipilimumab on immune modulation. Thus, genomic expression patterns of whole blood collected at baseline and during on-study treatment as specified in Table 5.1-2 may be assessed by Affymetrix microarray profiling, qRT-PCR or other gene expression profiling technology, with a particular emphasis on genes with relevant immune function. In addition, RNA or DNA derived from this peripheral blood sample may be assessed for rearrangements in the T cell receptor (TCR) in T cells within the peripheral blood. An assessment of somatic TCR rearrangements by PCR, sequencing or NextGen sequencing approach will provide information regarding the clonality of a T cell repertoire, which may change with nivolumab and/or ipilimumab treatment. In addition, baseline T cell repertoire may be predictive of nivolumab and/or ipilimumab benefit.

5.5.5 Whole Blood for SNP Assessment

Whole blood samples for exploratory pharmacogenetic assessment will be collected from all participants and put in frozen storage. Genomic DNA will be extracted and subsequently assessed for single nucleotide polymorphisms (SNPs) and other genetic variations in candidate genes that may predispose participants to nivolumab or ipilimumab benefit or adverse events (unless restricted by local requirements.) Such genes include, but are not limited to PD-1, PD-L1, PD-L2, and CTLA-4. Additional use of these data may include correlative analyses aimed at identifying genotypic associations with clinically-relevant biomarkers identified by other methodologies described in this section.

5.5.6 Collection of fecal samples

A Fecal sample will be collected at screening from all participants and put in frozen storage. Total DNA is extracted from fecal samples and 16S rRNA sequencing will be performed to determine the gut bacterial composition.

5.6 Results of Central Assessments

Not applicable.

6 ADVERSE EVENTS

An ***Adverse Event (AE)*** is defined as any new untoward medical occurrence or worsening of a pre-existing medical condition in a clinical investigation participant administered an investigational (medicinal) product and that does not necessarily have a causal relationship with this treatment. An AE can therefore be any unfavourable and unintended sign (such as an abnormal laboratory finding), symptom, or disease temporally associated with the use of investigational product, whether or not considered related to the investigational product.

The causal relationship to study drug is determined by a physician and should be used to assess all adverse events (AE). The casual relationship can be one of the following:

> Related: There is a reasonable causal relationship between study drug administration and the AE.
>
> Not related: There is not a reasonable causal relationship between study drug administration and the AE.

The term "reasonable causal relationship" means there is evidence to suggest a causal relationship.

Adverse events can be spontaneously reported or elicited during open-ended questioning, examination, or evaluation of a participant. (In order to prevent reporting bias, participants should not be questioned regarding the specific occurrence of one or more AEs.)

6.1 Serious Adverse Events

A S***erious Adverse Event (SAE)*** is any untoward medical occurrence that at any dose:

- results in death
- is life-threatening (defined as an event in which the participant was at risk of death at the time of the event; it does not refer to an event which hypothetically might have caused death if it were more severe)
- requires inpatient hospitalization or causes prolongation of existing hospitalization (see **NOTE** below)
- results in persistent or significant disability/incapacity
- is a congenital anomaly/birth defect
- is an important medical event (defined as a medical event(s) that may not be immediately life-threatening or result in death or hospitalization but, based upon appropriate medical and scientific judgment, may jeopardize the participant or may require intervention [e.g., medical, surgical] to prevent one of the other serious outcomes listed in the definition above.) Examples of such events include, but are not limited to, intensive treatment in an emergency room or at home for allergic bronchospasm; blood dyscrasias or convulsions that do not result in hospitalization.) Potential drug induced liver injury (DILI) is also considered an important medical event. (See Section 6.6 for the definition of potential DILI.)

Suspected transmission of an infectious agent (e.g., pathogenic or non-pathogenic) via the study drug is an SAE.

Although pregnancy, overdose, and potential drug induced liver injury (DILI) are not always serious by regulatory definition, these events must be handled as SAEs. (See Section 6.1.1for reporting pregnancies).

For the purposes of this study, the progression of disease under study will not be classed as an SAE.

Any component of a study endpoint that is considered related to study therapy (e.g., death is an endpoint, if death occurred due to anaphylaxis, anaphylaxis must be reported) should be reported as SAE (see Section 6.1.1for reporting details.

NOTE:

The following hospitalizations will not be considered SAEs:

- a visit to the emergency room or other hospital department < 24 hours, that does not result in admission (unless considered an important medical or life-threatening event)
- elective surgery, planned prior to signing consent
- admissions as per protocol for a planned medical/surgical procedure
- routine health assessment requiring admission for baseline/trending of health status (e.g., routine colonoscopy)
- medical/surgical admission other than to remedy ill health and planned prior to entry into the study. Appropriate documentation is required in these cases
- admission encountered for another life circumstance that carries no bearing on health status and requires no medical/surgical intervention (e.g., lack of housing, economic inadequacy, caregiver respite, family circumstances, administrative reason).
- Admission for administration of anticancer therapy in the absence of any other SAEs (applies to oncology protocols)

6.1.1 Serious Adverse Event Collection and Reporting

Following the participant's written consent to participate in the study, all SAEs, whether related or not related to study drug, must be collected, including those thought to be associated with protocol-specified procedures. All SAEs must be collected that occur from registration and within 100 days of discontinuation of dosing. If applicable, SAEs must be collected that relate to any later protocol-specified procedure (e.g., a follow-up skin biopsy).

The investigator should report any SAE that occurs after these time periods and that is believed to be related to study drug or protocol-specified procedure.

An SAE report should be completed for any event where doubt exists regarding its seriousness.

If the investigator believes that an SAE is not related to study drug, but is potentially related to the conditions of the study (such as withdrawal of previous therapy or a complication of a study procedure), the relationship should be specified in the narrative section of the SAE Report Form.

SAEs, whether related or not related to study drug, and pregnancies must be reported to ONJCRI and BMS within 24 hours of awareness of the event. SAEs must be recorded on the SAE Report Form; pregnancies on a Pregnancy Surveillance Form (electronic or paper forms). Notify ONJCRI and BMS via email:

Report to:	Email:
ONJCRI	trials@onjcri.org.au
BMS Worldwide Safety	worldwide.safety@bms.com

All Serious Adverse Events must be reported to ONJCRI, BMS Worldwide Safety and the lead Human Research Ethics Committee.

Site specific forms will be requested for review by the sponsor or sponsors representative.

The sponsor/investigator will reconcile SAEs reported in the clinical database (SAE line listings) with SAE cases transmitted to ONJCRI/BMS Global Pharmacovigilance (GPV&E); worldwide.safety@bms.com. This will be done quarterly and prior to the database lock or final data summary.
If only limited information is initially available, follow-up reports are required. (Note: Follow-up SAE reports should include the same investigator term(s) initially reported.)

If an ongoing SAE changes in its intensity or relationship to study drug or if new information becomes available, a follow-up SAE report should be sent within 24 hours to ONJCRI and BMS using the same procedure used for transmitting the initial SAE report above (via email).

All SAEs should be followed to resolution or stabilization.

6.2 Non-serious Adverse Events

A ***non-serious adverse event*** is an AE not classified as serious.

6.2.1 Non-serious Adverse Event Collection and Reporting

The collection of non-serious AE information should begin at initiation of study drug. Non-serious AE information should also be collected from the start of a placebo lead-in period or other observational period intended to establish a baseline status for the participants.

Non-serious AEs should be followed to resolution or stabilization, or reported as SAEs if they become serious (Section 6.1.1). Follow-up is also required for non-serious AEs that cause interruption or discontinuation of study drug and for those present at the end of study treatment as appropriate. All identified non-serious AEs must be recorded and described on the non-serious AE page of the CRF (paper or electronic).

All non-serious adverse events (not only those deemed to be treatment-related) should be collected continuously during the treatment period and for a minimum of 100 days following the last dose of study treatment.

Completion of supplemental CRFs may be requested for AEs and/or laboratory abnormalities that are reported/identified during the course of the study.

6.3 Laboratory Test Result Abnormalities

The following laboratory test result abnormalities should be captured on the non-serious AE CRF page or SAE Report Form (paper or electronic) as appropriate:

- Any laboratory test result that is clinically significant or meets the definition of an SAE

- Any laboratory test result abnormality that required the participant to have study drug discontinued or interrupted
- Any laboratory test result abnormality that required the participant to receive specific corrective therapy.

It is expected that wherever possible, the clinical rather than laboratory term would be used by the reporting investigator (e.g., anaemia versus low haemoglobin value).

6.4 Pregnancy

If, following initiation of the investigational product, it is subsequently discovered that a study participant is pregnant or may have been pregnant at the time of investigational product exposure, including during at least 6 half-lives after product administration, the investigational product will be permanently discontinued in an appropriate manner (e.g., dose tapering if necessary for participant safety).

Protocol-required procedures for study discontinuation and follow-up must be performed on the participant unless contraindicated by pregnancy (e.g., x-ray studies). Other appropriate pregnancy follow-up procedures should be considered if indicated.

The investigator must immediately notify the BMS (or designee) Medical Monitor of this event and complete and forward a Pregnancy Surveillance Form to BMS (or designee) within 24 hours of awareness of the event and in accordance with SAE reporting procedures described in Section 6.1.1.

Follow-up information regarding the course of the pregnancy, including perinatal and neonatal outcome and, where applicable, offspring information must be reported on the Pregnancy Surveillance Form.

Any pregnancy that occurs in a female partner of a male study participant should be reported to BMS. Information on this pregnancy will be collected on the Pregnancy Surveillance Form.

6.5 Overdose

All occurrences of overdose must be reported as SAEs (see Section 6.1.1 for reporting details).

6.6 Potential Drug Induced Liver Injury (DILI)

Wherever possible, timely confirmation of initial liver-related laboratory abnormalities should occur prior to the reporting of a potential DILI event. All occurrences of potential DILIs, meeting the defined criteria, must be reported as SAEs (see Section 6.1.1 for reporting details).

Potential drug induced liver injury is defined as:

1) ALT or AST elevation > 3 times upper limit of normal (ULN)

   AND

2) Total bilirubin > 2 times ULN, without initial findings of cholestasis (elevated serum alkaline phosphatase),

   AND

3) No other immediately apparent possible causes of AT elevation and hyperbilirubinemia, including, but not limited to, viral hepatitis, pre-existing chronic or acute liver disease, or the administration of other drug(s) known to be hepatotoxic.

6.7 Other Safety Considerations

Any significant worsening noted during interim or final physical examinations, electrocardiogram, x-ray filming, any other potential safety assessment required or not required by protocol should also be recorded as a non-serious or serious AE, as appropriate, and reported accordingly.

7 STATISTICAL CONSIDERATIONS

7.1 Sample Size Determination

7.1.1 Initial cohort

A total of 60 patients will be enrolled across the three tumour streams. Patients will not be randomized; all patients will receive open label treatment.

Due to the fact that rare cancers are underrepresented, we aim to assess approximately 20 patients per cohort in order to determine the efficacy of these immunotherapies in rare cancers.

Statistics will be descriptive only in view of sample size and heterogeneity.

Efficacy will be examined as a function of tumor response and will determined by both RECIST 1.1 and irRC (33), change in immune cell infiltrate and/or volume will be quantitated and analysed by cohort. Multivariate analysis will be applied. Overall survival will be estimated using the Kaplan-Meier estimation of survival. Since numbers will be insufficient for meaningful PFS & OS estimates based on tumor type, these analyses will be exploratory and patients will be classified based on non-standard criteria such as biomarker status. It is anticipated that biomarkers can be identified that can predict improved outcomes.
Documentation of PFS and OS will be conducted at different time points.

- The PFS analysis is targeted to occur after all participants have 9 months follow-up However, the required minimum follow-up for analysis of PFS is 6 months.
- The OS analysis is targeted to occur after all participants have 28 months follow-up. However, the required minimum follow-up for analysis of OS is 22 months.

Clinical activity observed in this study will be used to justify further study in relevant cancer populations.

7.1.2 Expansion cohort

A total of 60 further patients may be enrolled in this study. Patient selection will be from the rare cancer tumour streams described in the initial cohort, however cohorts will be filled based on eligible patients rather than limiting numbers to 20 per stream. Clinical activity and predictive biomarker analysis will match that undertaken for the initial cohort as described in section 7.1.1.

7.2 Populations for Analyses

- All Enrolled Participants: All participants who signed an informed consent form and were registered into the trial.
- All Treated Participants: All participants who received at least one dose of any study medication.
- Biomarker Participants: All participants with available biomarker data.

7.3 Endpoints

7.3.1 Primary Endpoint

To determine the clinical efficacy of the combination treatment of ipilimumab with nivolumab in rare cancers

7.3.1.1 Clinical Benefit Rate

Clinical benefit rate for whole population (CR+PR+SD>3months) >20%

The primary objective will be measured by the endpoint of Clinical Benefit Rate (CBR). This is defined as the number of participants with a CR, PR or stable disease for the whole population and for each patient cohort. The CBR is defined as the response designation, as determined by the investigator, recorded between the date of registration and the date of objectively documented progression per RECIST 1.1 or the date of subsequent anti-cancer therapy, whichever occurs first. For participants without documented progression or subsequent therapy, all available response designations will contribute to the cbr assessment. Tumour assessments are scheduled to be performed at week 12, every 6 weeks up to week 49 and then every 12 weeks until disease progression.

7.3.2 Secondary Endpoint Exploratory Endpoint(s)

Secondary objective of this study is to identify whether a common predictive biomarker or immune signature can be identified in responding patients that can occur irrespective of tumour type.

Exploratory endpoints:

Characterisation of immune responses to nivolumab/ipilimumab and predictive biomarker analysis unrestricted by tumour histological type.

Characterisation of inflammatory changes in tumours including the trafficking of lymphocytes using functional imaging.

Descriptive endpoint: Analyses of PFS and OS will be conducted at different time points with PFS being analyzed first (PFS analysis time point) followed by analysis of OS (OS analysis time point). Except where otherwise noted, analyses will be conducted at both time points.

Duration of and time to response will be measured by the endpoints duration of objective response (DOOR) and time to objective response (TTOR). DOOR is defined as the time between the date of first response to the date of first documented tumour progression (per RECIST 1.1) or death due to any cause. Participants who neither progress nor die will be censored on the date of their last tumour assessment. TTOR is defined as the time from enrolment to the date of the first documented CR or PR. DOOR and TTOR will be evaluated for responders (CR or PR) only.

Safety and tolerability will be measured by the incidence of adverse events, serious adverse events, deaths, and laboratory abnormalities.

Other exploratory endpoints for biomarkers are described in Section 5.5.

7.4 Analyses

7.4.1 Demographics and Baseline Characteristics

Demographic and baseline characteristics will be summarized in all participants by diagnostic group using descriptive statistics.

7.4.2 Safety Analyses

Safety analyses will be performed in all treated participants. Descriptive statistics of safety will be presented using National Cancer Institute (NCI) Common Terminology Criteria for Adverse Events (CTCAE) version 4.0 by treatment group. All on-study AEs, treatment-related AEs, SAEs, and treatment-related SAEs will be tabulated using worst grade per NCI CTCAE v 4.0 criteria by system organ class and preferred term. On-study lab parameters including haematology, chemistry, liver function, and renal function will be summarized using worst grade NCI CTCAE v 4.0 criteria.

7.4.3 Biomarker Analyses

Exploratory biomarker analyses will be descriptive only.

7.5 Interim Analyses

Not applicable.

8 STUDY MANAGEMENT

8.1 Compliance

8.1.1 Compliance with the Protocol and Protocol Revisions

The study shall be conducted as described in this approved protocol. All revisions to the protocol must be discussed with BMS. The investigator should not implement any deviation or change to the protocol without prior review and documented approval/favourable opinion from the IRB/IEC of an amendment, except where necessary to eliminate an immediate hazard(s) to study participants.

If a deviation or change to a protocol is implemented to eliminate an immediate hazard(s) prior to obtaining IRB/IEC approval/favourable opinion, as soon as possible the deviation or change will be submitted to:

- IRB/IEC for review and approval/favourable opinion
- BMS
- Regulatory Authority(ies), if required by local regulations

Documentation of approval signed by the chairperson or designee of the IRB(s)/IEC(s) must be sent to BMS.

If an amendment substantially alters the study design or increases the potential risk to the participant: (1) the consent form must be revised and submitted to the IRB(s)/IEC(s) for review and approval/favourable opinion; (2) the revised form must be used to obtain consent from participants currently enrolled in the study if they are affected by the amendment; and (3) the new form must be used to obtain consent from new participants prior to enrolment.

If the revision is done via an administrative letter, investigators must inform their IRB(s)/IEC(s).

8.1.2 Monitoring

Monitors may visit study site locations periodically to assess the data quality and study integrity. On site they will review study records and directly compare them with source documents, discuss the conduct of the study with the investigator, and verify that the facilities remain acceptable.

8.1.3 Investigational Site Training

The sponsor will provide quality investigational staff training prior to study initiation. Training topics will include but are not limited to: GCP, AE reporting, study details and procedure, electronic CRFs, study documentation, informed consent, and enrolment of WOCBP.

8.2 Records

8.2.1 Records Retention

The investigator must retain all study records and source documents for the maximum period required by applicable regulations and guidelines, or institution procedures, or for the period specified by the sponsor, whichever is longer. The investigator must contact the sponsor prior to destroying any records associated with the study.

The sponsor will notify the investigator when the study records are no longer needed.

If the investigator withdraws from the study (e.g., relocation, retirement), the records shall be transferred to a mutually agreed upon designee (e.g., another investigator, IRB). Notice of such transfer will be given in writing to the sponsor.

8.2.2 Study Drug Records

It is the responsibility of the investigator to ensure that a current disposition record of investigational product (those supplied by BMS) is maintained at each study site where study drug are inventoried and dispensed. Records or logs must comply with applicable regulations and guidelines and should include:

- amount received and placed in storage area
- amount currently in storage area
- label identification number or batch number
- amount dispensed to and returned by each participant, including unique participant identifiers
- amount transferred to another area/site for dispensing or storage
- non-study disposition (e.g., lost, wasted)
- amount destroyed at study site, if applicable
- amount returned to BMS
- retain samples for bioavailability/bioequivalence, if applicable
- dates and initials of person responsible for Investigational Product dispensing/accountability, as per the Delegation of Authority Form.

BMS will provide forms to facilitate inventory control if the investigational site does not have an established system that meets these requirements.

8.2.3 Case Report Forms

An investigator is required to prepare and maintain adequate and accurate case histories designed to record all observations and other data pertinent to the investigation on each individual treated or entered as a control in the investigation. Data that are derived from source documents and reported on the CRF must be consistent with the source documents or the discrepancies must be explained. Additional clinical information may be collected and analyzed in an effort to enhance understanding of product safety. CRFs may be requested for AEs and/or laboratory abnormalities that are reported or identified during the course of the study.

CRFs will be prepared for all data collection fields.

The confidentiality of records that could identify participants must be protected, respecting the privacy and confidentiality rules in accordance with the applicable regulatory requirement(s).

The investigator will maintain a signature sheet to document signatures and initials of all persons authorized to make entries and/or corrections on CRFs.

The completed CRF, including any paper or electronic SAE/pregnancy CRFs, must be promptly reviewed, signed, and dated by the investigator or qualified physician who is a sub investigator and who is delegated this task on the Delegation of Authority Form. The investigator must retain a copy of the CRFs including records of the changes and corrections.

8.3 Clinical Study Report and Publications

The Principal Investigator will sign the clinical study report.

The data collected during this study are confidential. Any publications or abstracts arising from this study require approval of the sponsor prior to publication or presentation and must adhere to the sponsor’s publication requirements as set forth in the approved clinical trial agreement (CTA). All draft publications, including abstracts or detailed summaries of any proposed presentations, must be submitted to the sponsor and BMS at the earliest practicable time for review, but at any event not less than 30 days before submission or presentation unless otherwise set forth in the CTA. BMS shall have the right to delete any confidential or proprietary information contained in any proposed presentation or abstract and may delay publication for up to 60 days for purposes of filing a patent application.

9 GLOSSARY OF TERMS

Term	Definition
Adverse Reaction	An adverse event that is considered by either the investigator or BMS as related to the investigational product
Unexpected Adverse Reaction	An adverse reaction, the nature or severity of which is not consistent with the applicable product information (e.g., Investigator Brochure for an unapproved investigational product)
Serious Adverse Event	Serious adverse event defined as any untoward medical occurrence that at any dose: results in death; is life threatening (defined as an event in which the participant was at risk of death at the time of the event; it does not refer to an event which hypothetically might have caused death if it were more severe), requires inpatient hospitalization or causes prolongation of existing hospitalization; results in persistent or significant disability/incapacity, is a congenital anomaly/birth defect; is an important medical event (defined as a medical event(s) that may not be immediately life threatening or result in death or hospitalization but, based upon appropriate medical and scientific judgment, may jeopardize the participant or may require intervention [e.g., medical, surgical] to prevent one of the other serious outcomes listed in the definition above). Examples of such events include, but are not limited to, intensive treatment in an emergency room or at home for allergic bronchospasm; blood dyscrasias or convulsions that do not result in hospitalization.). For reporting purposes only, BMS also considers the occurrence of pregnancy, overdose (regardless of association with an AE), and cancer as important medical events.

10 LIST OF ABBREVIATIONS

Term	Definition
AE	adverse event
ACLS	advanced cardiac life support
ADL	Activities of daily living
AI	accumulation index
AI_AUC	AUC Accumulation Index; ratio of AUC(TAU) at steady state to AUC(TAU) after the first dose
AI_Cmax	Cmax Accumulation Index; ratio of Cmax at steady state to Cmax after the first dose
AI_Ctau	Ctau Accumulation Index; ratio of Ctau at steady state to Ctau after the first dose
AJCC	American Joint Committee on Cancer
ALT	alanine aminotransferase
ANC	absolute neutrophil count
ANOVA	analysis of variance
aPTT	activated partial thromboplastin time
AST	aspartate aminotransferase
AT	aminotransaminases
AUC	area under the concentration-time curve
AUC(INF)	area under the concentration-time curve from time zero extrapolated to infinite time
AUC(0-T)	area under the concentration-time curve from time zero to the time of the last quantifiable concentration
AUC(TAU)	area under the concentration-time curve in one dosing interval
A-V	atrioventricular
β-HCG	beta-human chorionic gonadotrophin
BA/BE	bioavailability/bioequivalence
%BE	percent biliary excretion
BID, bid	bis in die, twice daily
BLQ	below limit of quantification
BMI	body mass index
BMS	Bristol-Myers Squibb
BP	blood pressure
BRt	Total amount recovered in bile

Term	Definition
%BRt	Total percent of administered dose recovered in bile
BSA	Body surface area
BUN	blood urea nitrogen
C	Celsius
C12	concentration at 12 hours
C24	concentration at 24 hours
Ca^{++}	calcium
Cavg	average concentration
CBC	complete blood count
Cexpected-tau	expected concentration in a dosing interval
CFR	Code of Federal Regulations
CI	confidence interval
$C1^{-}$	chloride
CLcr	creatinine clearance
CLD	Dialysate clearance of drug from plasma/serum
CLNR	nonrenal clearance
CLR	renal clearance
CLT	total body clearance
CLT/F (or CLT)	apparent total body clearance
CLT/F/fu or CLT/fu	Apparent total body clearance of free drug or Total body clearance of free drug (if IV)
cm	centimetre
Cmax, CMAX	maximum observed concentration
Cmin, CMIN	trough observed concentration
CNS	Central nervous system
CRC	Clinical Research Centre
CRF	Case Report Form, paper or electronic
Ct	Expected concentration at a certain time, usually at the end of an expected future dosing interval (e.g., concentration at 24 hours, concentration at 12 hours, etc.)
Ctau	Concentration in a dosing interval (e.g., concentration at 24 hours, concentration at 12 hours, etc.)
Ctrough	Trough observed plasma concentration
CTCAE	Common Terminology Criteria for Adverse Events

Term	Definition
CV	coefficient of variation
CYP	cytochrome p-450
D/C	discontinue
dL	decilitre
DRt	Total amount recovered in dialysate
%DRt	Total percent of administered dose recovered in dialysate
DSM IV	Diagnostic and Statistical Manual of Mental Disorders (4th Edition)
EA	extent of absorption
ECG	electrocardiogram
eCRF	Electronic Case Report Form
EDC	Electronic Data Capture
EEG	electroencephalogram
e.g.	exempli gratia (for example)
ESR	Expedited Safety Report
F	bioavailability
Fb	fraction of bound drug
FDA	Food and Drug Administration
FI	fluctuation Index ([Cmax-Ctau)/Cavg])
FRt	total amount recovered in feces
%FRt	total percent of administered dose recovered in feces
FSH	follicle stimulating hormone
fT4	Free thyroxine
%FE	percent fecal excretion
fu	fraction of unbound drug
g	gram
G	Grade
GC	gas chromatography
GCP	Good Clinical Practice
G criteria	adjusted R^2 value of terminal elimination phase
GGT	gamma-glutamyl transferase
GFR	glomerular filtration rate
h(hrs)	Hour (hours)
HBsAg	hepatitis B surface antigen

Term	Definition
HBV	hepatitis B virus
HCV	hepatitis C virus
HCO_3^-	bicarbonate
HIV	Human Immunodeficiency Virus
HR	heart rate
HRT	hormone replacement therapy
ICD	International Classification of Diseases
ICH	International Conference on Harmonisation
ID	Infectious Disease
i.e.	id est (that is)
IEC	Independent Ethics Committee
IMP	investigational medicinal products
IND	Investigational New Drug Exemption
I/O	Immuno-oncology
IRB	Institutional Review Board
IU	International Unit
IV	intravenous
IVIG	Intravenous immunoglobulin
K	slope of the terminal phase of the log concentration-time curve
K_3EDTA	potassium ethylenediaminetetraacetic acid
K^+	potassium
kg	kilogram
λz	terminal disposition rate constant
L	litre
LC	liquid chromatography
LDH	lactate dehydrogenase
ln	natural logarithm
LFT	Liver function test
Lz_Start	The time point starting the log-linear elimination phase defining the terminal half life
Lz_End	The time point ending the log-linear elimination phase defining the terminal half life

Term	Definition
Lz_N	Number of time points in the log-linear elimination phase defining the terminal half life
mg	milligram
Mg/kg	Milligram per kilogram
Mg^{++}	magnesium
MIC	minimum inhibitory concentration
min	minute
mL	millilitre
mmHg	millimetres of mercury
MR_AUC(0-T)	Ratio of metabolite AUC(0-T) to parent AUC(0-T), corrected for molecular weight
MR_AUC(INF)	Ratio of metabolite AUC(INF) to parent AUC(INF), corrected for molecular weight
MR_AUC(TAU)	Ratio of metabolite AUC(TAU) to parent AUC(TAU), corrected for molecular weight
MR_Cmax	Ratio of metabolite Cmax to parent Cmax, corrected for molecular weight
MR_Ctau	Ratio of metabolite Ctau to parent Ctau, corrected for molecular weight
MRI	Magnetic resonance imaging
MRT	mean residence time
MS	mass spectrometry
MTD	maximum tolerated dose
µg	microgram
N	number of participants or observations
Na^{+}	sodium
N/A	not applicable
NCI	National Cancer Institute
ng	nanogram
NIMP	non-investigational medicinal products
NSAID	nonsteroidal anti-inflammatory drug
pAUCe	Extrapolated partial AUC from last quantifiable concentration to infinity
ORR	Objective Response Rate
OS	Overall Survival
Pb	percent of bound drug
PD	pharmacodynamics

Term	Definition
PFS	Progression Free Survival
PK	pharmacokinetics
PO	per os (by mouth route of administration)
PT	prothrombin time
PTT	partial thromboplastin time
Pu	percent of unbound drug
QC	quality control
QD, qd	quaque die, once daily
R^2	coefficient of determination
RBC	red blood cell
SAE	serious adverse event
SD	standard deviation
SOP	Standard Operating Procedures
sp.	species
t	temperature
T	time
TAO	Trial Access Online, the BMS implementation of an EDC capability
T.bili	Total bilirubin
T-HALF	Half life
T-HALFeff_AUC	Effective elimination half-life that explains the degree of AUC accumulation observed
T-HALFeff_Cmax	Effective elimination half-life that explains the degree of Cmax accumulation observed)
TID, tid	ter in die, three times a day
Tmax, TMAX	time of maximum observed concentration
TR_AUC(0-T)	AUC(0-T) treatment ratio
TR_AUC(INF)	AUC(INF) treatment ratio
TR_Cmax	Cmax treatment ratio
TSH	Thyroid stimulating hormone
UEC	Urea, Electrolytes and Creatinine
ULN	Upper limit of normal
UR	urinary recovery
%UR	percent urinary recovery
URt	total amount recovered in urine
%URt	total percent of administered dose recovered in urine

Term	Definition
UV	ultraviolet
Vss/F (or Vss)	apparent volume of distribution at steady state
Vz	Volume of distribution of terminal phase (if IV and if multi-exponential decline)
W	washout
WBC	white blood cell
WHO	World Health Organization
WOCBP	women of childbearing potential
x g	times gravity

11 REFERENCES

1. Gatta G, van der Zwan JM, Casali PG, Siesling S, Dei Tos AP, Kunkler I, et al. Rare cancers are not so rare: the rare cancer burden in Europe. Eur J Cancer. 2011;47(17):2493-511.
2. Gatta G, Ciccolallo L, Kunkler I, Capocaccia R, Berrino F, Coleman MP, et al. Survival from rare cancer in adults: a population-based study. Lancet Oncol. 2006;7(2):132-40.
3. Ascierto PA, Addeo R, Carteni G, Daniele B, De Laurentis M, Ianniello GP, et al. The role of immunotherapy in solid tumors: report from the Campania Society of Oncology Immunotherapy (SCITO) meeting, Naples 2014. J Transl Med. 2014;12:291.
4. Casali PG, Bruzzi P, Bogaerts J, Blay JY, Rare Cancers Europe Consensus P. Rare Cancers Europe (RCE) methodological recommendations for clinical studies in rare cancers: a European consensus position paper. Ann Oncol. 2015;26(2):300-6.
5. Schreiber RD, Old LJ, Smyth MJ. Cancer immunoediting: integrating immunity's roles in cancer suppression and promotion. Science. 2011;331(6024):1565-70.
6. Effect of MDX-1106 on CD4+ T cells during an allogeneic mixed lymphocyte reaction (MLR) (Medarex Study No. MDX-1106-026-R). Bristol-Myers Squibb Pharmaceutical Research Institute; 2006. Document Control No. 930046581
7. Freeman GJ, Long AJ, Iwai Y, Bourque K, Chernova T, Nishimura H, et al. Engagement of the PD-1 immunoinhibitory receptor by a novel B7 family member leads to negative regulation of lymphocyte activation. J Exp Med. 2000;192(7):1027-34.
8. Latchman Y, Wood CR, Chernova T, Chaudhary D, Borde M, Chernova I, et al. PD-L2 is a second ligand for PD-1 and inhibits T cell activation. Nat Immunol. 2001;2(3):261-8.
9. Carter L, Fouser LA, Jussif J, Fitz L, Deng B, Wood CR, et al. PD-1:PD-L inhibitory pathway affects both CD4(+) and CD8(+) T cells and is overcome by IL-2. Eur J Immunol. 2002;32(3):634-43.
10. Chemnitz JM, Parry RV, Nichols KE, June CH, Riley JL. SHP-1 and SHP-2 associate with immunoreceptor tyrosine-based switch motif of programmed death 1 upon primary human T cell stimulation, but only receptor ligation prevents T cell activation. J Immunol. 2004;173(2):945-54.
11. Sheppard KA, Fitz LJ, Lee JM, Benander C, George JA, Wooters J, et al. PD-1 inhibits T-cell receptor induced phosphorylation of the ZAP70/CD3zeta signalosome and downstream signaling to PKCtheta. FEBS Lett. 2004;574(1-3):37-41.
12. Nishimura H, Honjo T. PD-1: an inhibitory immunoreceptor involved in peripheral tolerance. Trends Immunol. 2001;22(5):265-8.
13. Nishimura H, Nose M, Hiai H, Minato N, Honjo T. Development of lupus-like autoimmune diseases by disruption of the PD-1 gene encoding an ITIM motif-carrying immunoreceptor. Immunity. 1999;11(2):141-51.
14. Nishimura H, Okazaki T, Tanaka Y, Nakatani K, Hara M, Matsumori A, et al. Autoimmune dilated cardiomyopathy in PD-1 receptor-deficient mice. Science. 2001;291(5502):319-22.
15. Okazaki T, Tanaka Y, Nishio R, Mitsuiye T, Mizoguchi A, Wang J, et al. Autoantibodies against cardiac troponin I are responsible for dilated cardiomyopathy in PD-1-deficient mice. Nat Med. 2003;9(12):1477-83.
16. Salama AD, Chitnis T, Imitola J, Ansari MJ, Akiba H, Tushima F, et al. Critical role of the programmed death-1 (PD-1) pathway in regulation of experimental autoimmune encephalomyelitis. J Exp Med. 2003;198(1):71-8.
17. Blazar BR, Carreno BM, Panoskaltsis-Mortari A, Carter L, Iwai Y, Yagita H, et al. Blockade of programmed death-1 engagement accelerates graft-versus-host disease lethality by an IFN-gamma-dependent mechanism. J Immunol. 2003;171(3):1272-7.
18. Iwai Y, Terawaki S, Honjo T. PD-1 blockade inhibits hematogenous spread of poorly immunogenic tumor cells by enhanced recruitment of effector T cells. Int Immunol. 2005;17(2):133-44.
19. Iwai Y, Ishida M, Tanaka Y, Okazaki T, Honjo T, Minato N. Involvement of PD-L1 on tumor cells in the escape from host immune system and tumor immunotherapy by PD-L1 blockade. Proc Natl Acad Sci U S A. 2002;99(19):12293-7.
20. Strome SE, Dong H, Tamura H, Voss SG, Flies DB, Tamada K, et al. B7-H1 blockade augments adoptive T-cell immunotherapy for squamous cell carcinoma. Cancer Res. 2003;63(19):6501-5.
21. Dong H, Strome SE, Salomao DR, Tamura H, Hirano F, Flies DB, et al. Tumor-associated B7-H1 promotes T-cell apoptosis: a potential mechanism of immune evasion. Nat Med. 2002;8(8):793-800.
22. Hirano F, Kaneko K, Tamura H, Dong H, Wang S, Ichikawa M, et al. Blockade of B7-H1 and PD-1 by monoclonal antibodies potentiates cancer therapeutic immunity. Cancer Res. 2005;65(3):1089-96.
23. Blank C, Brown I, Peterson AC, Spiotto M, Iwai Y, Honjo T, et al. PD-L1/B7H-1 inhibits the effector phase of tumor rejection by T cell receptor (TCR) transgenic CD8+ T cells. Cancer Res. 2004;64(3):1140-5.

24. Konishi J, Yamazaki K, Azuma M, Kinoshita I, Dosaka-Akita H, Nishimura M. B7-H1 expression on non-small cell lung cancer cells and its relationship with tumor-infiltrating lymphocytes and their PD-1 expression. Clin Cancer Res. 2004;10(15):5094-100.
25. Thompson RH, Gillett MD, Cheville JC, Lohse CM, Dong H, Webster WS, et al. Costimulatory B7-H1 in renal cell carcinoma patients: Indicator of tumor aggressiveness and potential therapeutic target. Proc Natl Acad Sci U S A. 2004;101(49):17174-9.
26. Ohigashi Y, Sho M, Yamada Y, Tsurui Y, Hamada K, Ikeda N, et al. Clinical significance of programmed death-1 ligand-1 and programmed death-1 ligand-2 expression in human esophageal cancer. Clin Cancer Res. 2005;11(8):2947-53.
27. Tsushima F, Tanaka K, Otsuki N, Youngnak P, Iwai H, Omura K, et al. Predominant expression of B7-H1 and its immunoregulatory roles in oral squamous cell carcinoma. Oral Oncol. 2006;42(3):268-74.
28. Thompson RH, Webster WS, Cheville JC, Lohse CM, Dong H, Leibovich BC, et al. B7-H1 glycoprotein blockade: a novel strategy to enhance immunotherapy in patients with renal cell carcinoma. Urology. 2005;66(5 Suppl):10-4.
29. Dong H, Chen L. B7-H1 pathway and its role in the evasion of tumor immunity. J Mol Med (Berl). 2003;81(5):281-7.
30. Azuma T, Yao S, Zhu G, Flies AS, Flies SJ, Chen L. B7-H1 is a ubiquitous antiapoptotic receptor on cancer cells. Blood. 2008;111(7):3635-43.
31. Thompson RH, Gillett MD, Cheville JC, Lohse CM, Dong H, Webster WS, et al. Costimulatory molecule B7-H1 in primary and metastatic clear cell renal cell carcinoma. Cancer. 2005;104(10):2084-91.
32. Hino R, Kabashima K, Kato Y, Yagi H, Nakamura M, Honjo T, et al. Tumor cell expression of programmed cell death-1 ligand 1 is a prognostic factor for malignant melanoma. Cancer. 2010;116(7):1757-66.
33. Curran MA, Montalvo W, Yagita H, Allison JP. PD-1 and CTLA-4 combination blockade expands infiltrating T cells and reduces regulatory T and myeloid cells within B16 melanoma tumors. Proc Natl Acad Sci U S A. 2010;107(9):4275-80.
34. Wolchok JD, Hoos A, O'Day S, Weber JS, Hamid O, Lebbe C, et al. Guidelines for the evaluation of immune therapy activity in solid tumors: immune-related response criteria. Clin Cancer Res. 2009;15(23):7412-20.
35. Non-Clinical Expedited Safety Report for Nivolumab (Ad Hoc CARES #54344.1); BMS DCN 930067627 1.0
36. Ji RR, Chasalow SD, Wang L, Hamid O, Schmidt H, Cogswell J, et al. An immune-active tumor microenvironment favors clinical response to ipilimumab. Cancer Immunol Immunother. 2012;61(7):1019-31.
37. Taube JM, Anders RA, Young GD, Xu H, Sharma R, McMiller TL, et al. Colocalization of inflammatory response with B7-h1 expression in human melanocytic lesions supports an adaptive resistance mechanism of immune escape. Sci Transl Med. 2012;4(127):127ra37. Epub 2012/03/31.
38. Atkins MB, Lotze MT, Dutcher JP, Fisher RI, Weiss G, Margolin K, et al. High-dose recombinant interleukin 2 therapy for patients with metastatic melanoma: analysis of 270 patients treated between 1985 and 1993. J Clin Oncol. 1999;17(7):2105-16.
39. Wolchok JD, Neyns B, Linette G, Negrier S, Lutzky J, Thomas L, et al. Ipilimumab monotherapy in patients with pretreated advanced melanoma: a randomised, double-blind, multicentre, phase 2, dose-ranging study. Lancet Oncol. 2010;11(2):155-64. Epub 2009/12/17.
40. Larkin J, Chiarion-Sileni V, Gonzalez R, Grob JJ, Cowey CL, Lao CD, et al. Combined Nivolumab and Ipilimumab or Monotherapy in Untreated Melanoma. N Engl J Med. 2015;373(1):23-34.
41. Brahmer J, Reckamp KL, Baas P, Crino L, Eberhardt WE, Poddubskaya E, et al. Nivolumab versus Docetaxel in Advanced Squamous-Cell Non-Small-Cell Lung Cancer. N Engl J Med. 2015;373(2):123-35.
42. Borghaei H, Paz-Ares L, Horn L, Spigel DR, Steins M, Ready NE, et al. Nivolumab versus Docetaxel in Advanced Nonsquamous Non-Small-Cell Lung Cancer. N Engl J Med. 2015;373(17):1627-39.
43. Hamanishi J, Mandai M, Ikeda T, Minami M, Kawaguchi A, Murayama T, et al. Safety and Antitumor Activity of Anti-PD-1 Antibody, Nivolumab, in Patients With Platinum-Resistant Ovarian Cancer. J Clin Oncol. 2015.
44. Powles T, Eder JP, Fine GD, Braiteh FS, Loriot Y, Cruz C, et al. MPDL3280A (anti-PD-L1) treatment leads to clinical activity in metastatic bladder cancer. Nature. 2014;515(7528):558-62. Epub 2014/11/28.
45. McDermott DF, Drake CG, Sznol M, Choueiri TK, Powderly JD, Smith DC, et al. Survival, Durable Response, and Long-Term Safety in Patients With Previously Treated Advanced Renal Cell Carcinoma Receiving Nivolumab. J Clin Oncol. 2015;33(18):2013-20.

46. Le DT, Uram JN, Wang H, Bartlett BR, Kemberling H, Eyring AD, et al. PD-1 Blockade in Tumors with Mismatch-Repair Deficiency. N Engl J Med. 2015;372(26):2509-20. Epub 2015/06/02.
47. Tumeh PC, Harview CL, Yearley JH, Shintaku IP, Taylor EJ, Robert L, et al. PD-1 blockade induces responses by inhibiting adaptive immune resistance. Nature. 2014;515(7528):568-71. Epub 2014/11/28.
48. Cebon J, Gedye C, John T, Davis ID. Immunotherapy of advanced or metastatic melanoma. Clin Adv Hematol Oncol. 2007;5(12):994-1006.
49. Andrews MC, Woods K, Cebon J, Behren A. Evolving role of tumor antigens for future melanoma therapies. Future oncology. 2014;10(8):1457-68. Epub 2014/07/24.
50. Taube JM, Klein A, Brahmer JR, Xu H, Pan X, Kim JH, et al. Association of PD-1, PD-1 ligands, and other features of the tumor immune microenvironment with response to anti-PD-1 therapy. Clin Cancer Res. 2014;20(19):5064-74. Epub 2014/04/10.
51. Trapani JA, Thia KY, Andrews M, Davis ID, Gedye C, Parente P, et al. Human perforin mutations and susceptibility to multiple primary cancers. Oncoimmunology. 2013;2(4):e24185.
52. Viaud S, Daillere R, Boneca IG, Lepage P, Pittet MJ, Ghiringhelli F, et al. Harnessing the intestinal microbiome for optimal therapeutic immunomodulation. Cancer Res. 2014;74(16):4217-21. Epub 2014/07/31.
