# Supplementary material for: A gut microbial signature for combination immune checkpoint blockade across cancer types
Source: Nat Med. 2024 Mar 1;30(3):797–809. doi: 10.1038/s41591-024-02823-z (PMC10957475; doi:10.1038/s41591-024-02823-z)
Supplement: Supplementary file 2 — Reporting Summary [file 41591_2024_2823_MOESM2_ESM.pdf]

nature portfolio

Corresponding author(s): Dr Ashray Gunjur
Dr Trevor D. Lawley

Last updated by author(s): 05 Jan 2024

Reporting Summary

Nature Portfolio wishes to improve the reproducibility of the work that we publish. This form provides structure for consistency and transparency in reporting. For further information on Nature Portfolio policies, see our Editorial Policies and the Editorial Policy Checklist.

Statistics

For all statistical analyses, confirm that the following items are present in the figure legend, table legend, main text, or Methods section.

n/a	Confirmed	
☐	☒	The exact sample size (*n*) for each experimental group/condition, given as a discrete number and unit of measurement
☐	☒	A statement on whether measurements were taken from distinct samples or whether the same sample was measured repeatedly
☐	☒	The statistical test(s) used AND whether they are one- or two-sided<br>*Only common tests should be described solely by name; describe more complex techniques in the Methods section.*
☐	☒	A description of all covariates tested
☐	☒	A description of any assumptions or corrections, such as tests of normality and adjustment for multiple comparisons
☐	☒	A full description of the statistical parameters including central tendency (e.g. means) or other basic estimates (e.g. regression coefficient) AND variation (e.g. standard deviation) or associated estimates of uncertainty (e.g. confidence intervals)
☐	☒	For null hypothesis testing, the test statistic (e.g. *F*, *t*, *r*) with confidence intervals, effect sizes, degrees of freedom and *P* value noted<br>*Give P values as exact values whenever suitable.*
☒	☐	For Bayesian analysis, information on the choice of priors and Markov chain Monte Carlo settings
☒	☐	For hierarchical and complex designs, identification of the appropriate level for tests and full reporting of outcomes
☐	☒	Estimates of effect sizes (e.g. Cohen's *d*, Pearson's *r*), indicating how they were calculated

Our web collection on statistics for biologists contains articles on many of the points above.

Software and code

Policy information about availability of computer code

Data collection	No specific software was used for data collection.
Data analysis	All software used for data analyses is publicly available, and is cited in the Methods section where possible. These software are:<br>command-line tools: bwa v0.7.17, trim-galore v0.6.7, BMTagger v3.101, SPAdes v3.14, MEGAHIT v1.24, MetaBAT 2 v2.13, MaxBin2 v2.2.4, CONCOCT v0.4, samtools v1.5, CheckM v1.1.2, MetaWRAP v1.2, CheckM2 v0.1.3, GUNC v1.0.5, GTDB-tk v2.1, pplacer v1.1, fastANI v1.3, bowtie2 v2.3.5, inStrain v1.3.0, dRep v2.0.0, Gapseq v1.2, HMMER v3.2.1, FastTree v2.1.0, rapidNJ v2.3.3, abricate v1.0.1.<br>Python 3: scikit-learn v1.1.1, imblearn v0.9.1, shap v0.41.0, numpy v1.23.3, pandas v1.4.3, matplotlib v3.5.1.<br>R v4.1.0: decontam v1.16.0, tidyverse v2.0.0, ggpubr v0.6.0, survival v3.5.5, survminer v0.4.9, table1 v1.4.3, phyloseq v1.12.0, microbiome v1.12.0, vegan v2.6.4, ggtree v3.2.1.<br>Code to replicate our supervised machine learning analysis of CA209-538 cohort data is deposited at https://github.com/agunjur/cancer_microbiome_CICB .

For manuscripts utilizing custom algorithms or software that are central to the research but not yet described in published literature, software must be made available to editors and reviewers. We strongly encourage code deposition in a community repository (e.g. GitHub). See the Nature Portfolio guidelines for submitting code & software for further information.

Data

Policy information about availability of data

All manuscripts must include a data availability statement. This statement should provide the following information, where applicable:

- Accession codes, unique identifiers, or web links for publicly available datasets
- A description of any restrictions on data availability
- For clinical datasets or third party data, please ensure that the statement adheres to our policy

All CA209-538 faecal shotgun metagenomic sequencing data (after first-pass human decontamination) has been deposited to the European Nucleotide Archive (study accession number ERP134027). The 1397 quality-controlled (near-complete) study-specific genomes used as the custom reference database have been deposited to zenodo (https://doi.org/10.5281/zenodo.10450122). CA209-538 clinical metadata and strain abundance data necessary to replicate our analyses is provided as the supplementary tables. The six publicly available shotgun metagenomics datasets were downloaded using the following accession numbers: 2022_SIMPSON: EGAS00001006982, 2022_LEE: PRJEB43119, 2022_MCCULLOCH: PRJNA762360, 2021_ANDREWS: EGAD00001006734, 2018_MATSON: PRJNA399742, 2017_FRANKEL: PRJNA397906. Permission to access the 2021_ANDREWS raw sequencing dataset for academic use was kindly provided by Dr Jennifer Wargo and The University of Texas M.D. Anderson Cancer Center. Permission to access the 2022_SIMPSON raw sequencing data was kindly provided by Professor Georgina Long and the Melanoma Institute of Australia. Associated clinical metadata for external datasets was collected from their relevant publications, the relevant sequencing repository or an associated github repository.

Research involving human participants, their data, or biological material

Policy information about studies with human participants or human data. See also policy information about sex, gender (identity/presentation), and sexual orientation and race, ethnicity and racism.

Reporting on sex and gender	CA209-538 clinical trial participant's self-reported sex was assessed by CA209-538 clinical investigators and recorded into the electronic case report form (eCRF). Sex is reported as a clinical variable in Table 1, Ext Table 1 and Ext Table 2, included as a metadata variable in the CA209-538 PERMANOVA analyses (Ext Fig 1d), and as a clinical feature in the supervised machine learning analyses (Fig 2b, Ext Fig 2b).
Reporting on race, ethnicity, or other socially relevant groupings	Race / ethnicity was not recorded or analysed.
Population characteristics	All CA209-538 participants were adults with advanced rare cancers falling into 3 histological cohorts: upper gastrointestinal / biliary tract (UGB), rare gynaecological (GYN) or neuro-endocrine neoplasms (NEN). All patients were adults (median age (years) 60 [range 20-82] and n=81 (68%) were female sex by self-report. Faecal samples were collected from most patients (n=106 'microbiome evaluable'). Patient-level metadata for microbiome-evaluable patients, including age, sex, body-mass index, ECOG performance status and study site is available in Supplementary table 3.<br>More details on trial inclusion/exclusion criteria are available at https://classic.clinicaltrials.gov/ct2/show/NCT02923934.
Recruitment	CA209-538 participants were screened for eligibility based on protocol inclusion criteria at 5 clinical sites across two states in Australia (3 sites in Victoria: Monash Health, Austin Health, Peter MacCallum Cancer Centre; 2 sites in New South Wales: Blacktown Hospital, Border Medical Oncology Unit). This involved referring medical practitioners sending detailed referrals to site principle investigators, who subsequently reviewed patients to confirm eligibility, willingness to participate, and sign trial informed consent. To aid recruitment, the clinical trial was advertised broadly, including via the 'Cancer Council Victoria: Victorian Cancer Trials Link' (https://trials.cancervic.org.au/details.aspx?ID=vctl_nct02923934). Patient geography and knowledge of the study may have biased study participation.
Ethics oversight	CA209-538 was approved across the 5 clinical sites by the Austin Health Human Research Ethics Committee (reference: HREC/16/Austin/152).

Note that full information on the approval of the study protocol must also be provided in the manuscript.

Field-specific reporting

Please select the one below that is the best fit for your research. If you are not sure, read the appropriate sections before making your selection.

☒ Life sciences ☐ Behavioural & social sciences ☐ Ecological, evolutionary & environmental sciences

For a reference copy of the document with all sections, see nature.com/documents/nr-reporting-summary-flat.pdf

Life sciences study design

All studies must disclose on these points even when the disclosure is negative.

Sample size	The primary objective of CA209-538 was to evaluate the clinical efficacy (by RECIST 1.1 response criteria) of ipilimumab and nivolumab in rare cancers. At the time of its design there was limited/no available data to estimate response rates of combination anti-PD-1 plus anti-CTLA-4 blockade in patients with these selected rare cancers, with CA209-538 designed to address this gap. Therefore, no statistical sample size or power calculation could be performed a priori.

Data exclusions	No data were excluded intentionally. A minority (n=14) of trial participants were unable to provide a stool specimen immediately prior to commencement of trial therapy. Statistical analyses of microbiome-evaluable (n=106) vs missing (n=14) patients is presented in Ext. table 1. There was a higher proportion of non-evaluable patients from one site (BLA, n=7), but no other suggestions of bias. All n=106 evaluable samples produced high-quality metagenomic sequencing data and were included in our analysis.
Replication	No technical replicates of metagenomic sequencing was performed, however PERMANOVA analysis suggests technical variables such as DNA plate were little contributors to microbial variance (Ext Fig 1d).
Randomization	Not applicable as CA209-538 was designed as a single-arm study to evaluate the efficacy of combination immune checkpoint blockade across rare cancers (representing novel indications), as above.
Blinding	Not applicable as CA209-538 is a single-arm study.

Reporting for specific materials, systems and methods

We require information from authors about some types of materials, experimental systems and methods used in many studies. Here, indicate whether each material, system or method listed is relevant to your study. If you are not sure if a list item applies to your research, read the appropriate section before selecting a response.

Materials & experimental systems

n/a	Involved in the study
☒	☐ Antibodies
☒	☐ Eukaryotic cell lines
☒	☐ Palaeontology and archaeology
☒	☐ Animals and other organisms
☐	☒ Clinical data
☒	☐ Dual use research of concern
☒	☐ Plants

Methods

n/a	Involved in the study
☒	☐ ChIP-seq
☒	☐ Flow cytometry
☒	☐ MRI-based neuroimaging

Clinical data

Policy information about clinical studies

All manuscripts should comply with the ICMJE guidelines for publication of clinical research and a completed CONSORT checklist must be included with all submissions.

Clinical trial registration	ClinicalTrials.gov Identifier: NCT02923934
Study protocol	The clinical outcomes for CA209-538 histological subgroups have been reported and published previously. Version 8 of the study protocol is included in the Supplementary materials with this submission.
Data collection	CA209-538 participants were recruited between October 2017 and February 2020 across 5 clinical sites in Australia 5 clinical sites across two states in Australia (3 sites in Victoria: Monash Health, Austin Health, Peter MacCallum Cancer Centre; 2 sites in New South Wales: Blacktown Hospital, Border Medical Oncology Unit). Clinical sites were hospital outpatient settings. Site clinical trial investigators recorded de-identified patient information into an eCRF.
Outcomes	The pre-defined primary outcome of CA209-538 was to evaluate the clinical efficacy of ipilimumab and nivolumab in patients with advanced rare cancer types, as determined using RECIST 1.1 'clinical benefit' (complete response + partial response + stable disease). The pre-defined secondary outcome of CA209-538 clinical trial was to identify whether a common predictive biomarker or immune signature can be identified in responding patients that can occur irrespective of tumour type. Samples collected include baseline whole blood, serum, peripheral blood mononuclear cells, archival formalin-fixed paraffin embedded tumour, and faecal samples. Specific methodology to define this 'common predictive biomarker' was not prespecified, and specific performance measures were not pre-defined.
